# Biological effects of a tested commercial fragrance formulation in rats: oxidative and molecular alterations and the protective role of vitamin E

**DOI:** 10.3389/fphar.2026.1882724

**Published:** 2026-08-26

**Authors:** Noora Saleh Al-Sowayan, Daniyah Khaled Al-Shebel

**Affiliations:** 1 Department of Biology, College of Science, Qassim University, Buraydah, Saudi Arabia; 2 Master’s Student and Biology Department, College of Science, Qassim University, Buraydah, Saudi Arabia

**Keywords:** antioxidants, fragrance formulation, fragrance inhalation, gene expression, oxidative stress

## Abstract

**Introduction:**

Fragrance products are widely used in daily life. Nevertheless, the biological effects associated with specific fragrance formulations remain insufficiently characterized due to the complexity of their chemical composition. This study aimed to provide an integrated multi-organ evaluation of the effects of inhaling a tested commercial fragrance formulation on oxidative stress, physiological parameters, neurotoxicity-related biomarkers, and gene expression in rats, and to assess the potential protective role of vitamin E.

**Methods:**

Twenty-five male albino rats were randomly assigned into five groups: control, olive oil, vitamin E, tested fragrance-exposed, and vitamin E plus tested fragrance-exposed. Animals were exposed for 30 consecutive days. Serum and tissue analyses included cardiac and renal markers (troponin, creatinine), arterial blood gases (ABG), blood pressure, heart rate, neurotoxicity-related markers (GFAP and UCH-L1), oxidative stress biomarkers (TAC, MDA, SOD, GSH-Px, CAT), and gene expression of CYP1A1, CYP1A2, Ckmt2, and Klf4.

**Results:**

Exposure to the tested fragrance formulation induced significant physiological and biochemical alterations, including increased serum creatinine (0.99 ± 0.13 mg/dL), troponin (3.41 ± 1.21 ng/mL), blood pressure (130.4 ± 4.4 mmHg), heart rate (375.8 ± 8.0 bpm), and MDA levels, alongside reduced antioxidant defenses (TAC, SOD, CAT, and GSH-Px). These alterations were accompanied by molecular changes reflected by upregulation of CYP1A1, CYP1A2, and Ckmt2, and downregulation of Klf4 expression. Vitamin E supplementation attenuated several of these alterations but did not fully restore normal levels.

**Conclusion:**

Under the present experimental conditions, repeated inhalation exposure to the tested fragrance formulation induced systemic oxidative stress and multi-organ physiological and molecular alterations in rats. Vitamin E provided partial protection, suggesting a limited but relevant antioxidant role against the biological effects associated with this tested fragrance mixture.

## Highlights

- The tested fragrance formulation induced oxidative stress and molecular alterations in rats.
- Increased troponin, creatinine, blood pressure, and heart rate were observed following exposure.
- Antioxidant defenses (SOD, CAT, GSH-Px, TAC) were significantly reduced.
- CYP1A1, CYP1A2, and Ckmt2 were upregulated, whereas Klf4 expression showed a downward trend.
- Vitamin E provided partial protective effects under the present experimental conditions.

## Introduction

Fragrance products contain volatile chemicals that have been used globally for thousands of years and are now widely incorporated into personal care products, food, perfumes, cosmetics, detergents, and agriculture (Kliszcz et al., 2021; Sharmeen et al., 2021; Schilling et al., 2010; Angelucci et al., 2014; Armanino et al., 2020). Volatile monoterpenes present in essential oils contribute to aroma and flavor enhancement in food and pharmaceutical applications (Rizvi et al., 2014). Commercial fragrance products are complex mixtures of natural and synthetic compounds, many of which have not been adequately evaluated for inhalation-related allergic or toxic effects (Opiekun et al., 2003). These products release volatile compounds into indoor environments, contributing to air pollution and generating secondary pollutants such as formaldehyde (Angulo Milhem et al., 2020; Steinemann, 2009).

Exposure to certain fragrance-related volatile compounds has been associated with adverse health effects. Odorants interact with olfactory receptors and rapidly transmit signals to the central nervous system (Sanz et al., 2017). Some fragrance molecules are capable of crossing the blood–brain barrier and influencing brain activity (Kutlu et al., 2008; Touhara and Vosshall, 2009). In addition, their interaction with indoor ozone can generate toxic secondary pollutants that irritate the respiratory tract and central nervous system and may impair immune function (Elberling et al., 2005). Such interactions may also contribute to oxidative imbalance through increased reactive oxygen species generation and disruption of normal cellular homeostasis (Elberling et al., 2005). While some studies have reported that fragrances exacerbate symptoms in asthma and COPD patients (Elberling et al., 2005), others have found no significant effects on respiratory function (Verstraelen et al., 2024; Vethanayagam et al., 2013). More recently, odors have been identified as potent neurological triggers. A cross-sectional study demonstrated that specific fragrances are strongly associated with migraine attacks, highlighting their impact on brain activity (Imai et al., 2023).

Cardiopulmonary effects associated with exposure to volatile fragrance compounds have also been reported. Daily exposure to essential oils for more than 1 h has been associated with increased heart rate, elevated blood pressure, and reduced peak flow rate (Lee et al., 2022). In animal studies, certain fragrances have been shown to increase cardiac stress markers such as creatine kinase and its gene expression (Adefolaju et al., 2016). Rose and lemon aromas affect heart rate differently due to their sedative and stimulatory properties (Anderson and Anderson, 1998; Taylor et al., 2014). Some fragrance products have also been reported to induce acute toxic effects in animal models, including pulmonary irritation (Anderson and Anderson, 1998). Neurotoxic compounds such as musk ambrette were discontinued due to their adverse effects on the nervous system (Taylor et al., 2014). Moreover, even low-level exposure to fragrances has been linked to central nervous system and behavioral effects in humans, including associations with autism in children of chemically sensitive mothers (Sowndhararajan and Kim, 2016; Steinemann, 2018).

Vitamin E, first identified in 1922, is a lipid-soluble antioxidant that plays a key role in cellular defense and reproduction (Verhagen et al., 2006). It scavenges reactive oxygen species, inhibits lipid peroxidation, and maintains membrane integrity (Blaner et al., 2021). Among its various forms, α-tocopherol is the most biologically active (Schneider, 2005; Niki, 2019). Its antioxidant activity is attributed to the hydroxyl group present on the tocochromanol ring (Peh et al., 2016). The biological and antioxidant functions of vitamin E are well established, with recent studies emphasizing its role in maintaining membrane stability and regulating reactive oxygen species generation (Niki, 2019; Sánchez-Vidaña et al., 2019). Despite the widespread use of fragrance products, limited research has addressed their long-term biological effects (Sowndhararajan and Kim, 2016).

Therefore, this study aimed to evaluate the multi-organ effects of inhaling a tested commercial fragrance formulation on oxidative stress, physiological parameters, histological alterations, neurotoxicity-related biomarkers, and gene expression, and to assess the potential protective role of vitamin E.

We hypothesized that repeated inhalation exposure to the tested fragrance formulation would induce oxidative stress, physiological disturbances, neurotoxicity-related changes, and gene expression alterations in the brain, heart, and lungs, and that vitamin E supplementation would provide partial protective effects.

## Materials and methods

### Animal experiment

Twenty-five normal male albino rats (135–145 g) were obtained from the College of Pharmacy, King Saud University. Animals were maintained under standard laboratory conditions (22 °C–28 °C, 12 h light/dark cycle, 50%–60% relative humidity) with free access to commercial pellets and water. Rats were housed in stainless-steel cages (55 × 39 × 28 cm).

The sample size was selected as an exploratory animal study design in accordance with ethical principles for animal research and the 3Rs approach (Replacement, Reduction, and Refinement), aiming to minimize animal use while allowing preliminary toxicological assessment. Similar fragrance-exposure studies in rats have used comparable group sizes and reported measurable biological outcomes (Gabriel-Brisibe et al., 2020; Andrade et al., 2014). The relatively small sample size is acknowledged as a limitation of the present study and should be considered when interpreting the findings.

Animals were randomly allocated into five groups (n = 5 per group).

1. Control group: untreated rats.
2. Olive oil group: received olive oil orally for 30 consecutive days.
3. Vitamin E group: received vitamin E dissolved in olive oil orally for 30 days.
4. Tested fragrance-exposed group: exposed to the tested fragrance formulation for 60 min/day for 30 days.
5. Combined group: received vitamin E in olive oil 1 h before exposure to the tested fragrance formulation daily for 30 days.

The olive oil group was included as a vehicle control for oral vitamin E administration and was not intended to serve as an inhalation control group.

All animal procedures were approved by the Qassim University Committee for Scientific Research Ethics (protocol code: 24-04-02; approval date: 9 September 2024).

**FIGURE 1:**
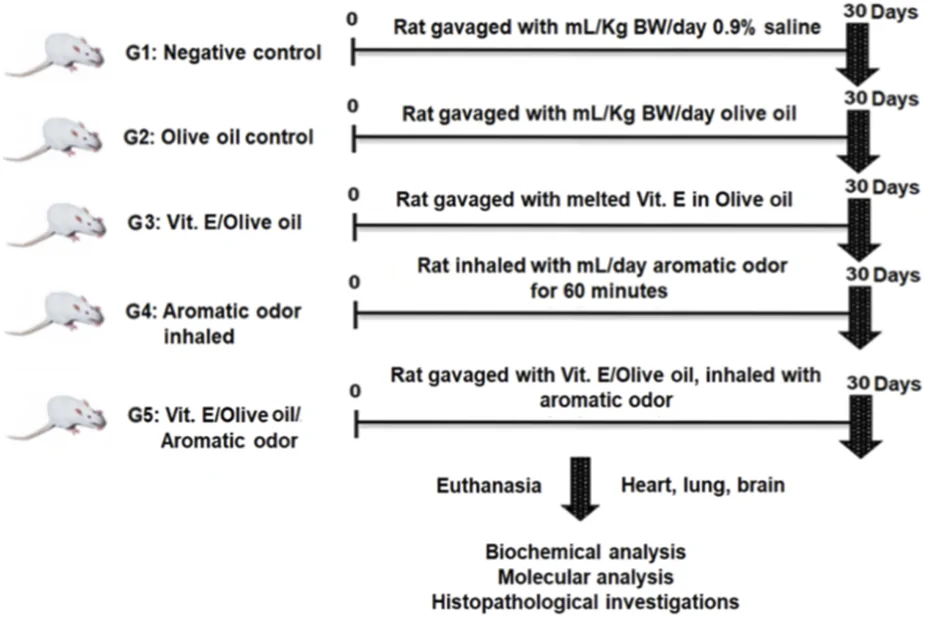
Shows the schematic illustration of the experimental design.

Figure 1 illustrates the experimental design.

### Anesthesia, terminal blood collection, euthanasia, and carcass disposal

Animals were anesthetized with ketamine (90 mg/kg body weight) and xylazine (10 mg/kg body weight), administered by intraperitoneal injection. Before any surgical incision, the depth of anesthesia was assessed using the toe/pedal pinch reflex. The absence of a motor response following firm pressure applied to a hind toe was considered confirmation of deep surgical anesthesia. After deep surgical anesthesia had been confirmed, terminal blood collection was performed by cardiac puncture for the measurement of serum biomarkers, including cardiac troponin T (cTnT), creatinine, glial fibrillary acidic protein (GFAP), and ubiquitin C-terminal hydrolase L1 (UCH-L1). This was followed by exsanguination and the immediate collection of the brain, heart, and lungs while the animals remained under deep anesthesia. Death was confirmed by the absence of heartbeat, spontaneous respiration, and reflex responses.

Following confirmation of death, the animal remains were placed in designated biological-waste bags and stored at −20 °C until transportation to the College of Agriculture and Veterinary Medicine, Qassim University. The remains were subsequently disposed of by incineration in accordance with the institution’s approved procedures for hazardous biological waste.

### Tested fragrance formulation exposure and vitamin E supplementation

A commercially available fragrance product was obtained from the local market in Buraydah, Saudi Arabia. The tested product was commercially obtained and selected as a representative fragrance mixture commonly available in the local market. The product was not prepared in-house. For exposure, 5 mL of the aromatic solution was placed in the exposure chamber for 60 min/day over 30 days (Sánchez-Vidaña et al., 2019). Therefore, the findings of this study apply only to the tested formulation under the present experimental conditions and should not be generalized to all fragrance products.

Vitamin E (D-α-tocopheryl acetate) was administered orally at 40 mg/kg/day to Groups 3 and 5 (Al-Sowayan, 2020).

### Biochemical and physiological measurements

1. Cardiac Troponin T (cTnT): Serum cTnT was measured using a rat ELISA kit (MyBioSource, San Diego, CA, United States; cat. no. MBS039759) according to the manufacturer’s protocol. A 50 μL serum sample was used per well and incubated for 60 min at 37 °C; absorbance was read at 450 nm within 15 min of adding the stop solution, using a Bio-Rad Model 680 microplate reader (Bio-Rad Laboratories, Hercules, CA, United States). Results were expressed in ng/mL.
2. Serum Creatinine: Serum creatinine was determined using the Rat Creatinine Assay Kit (Crystal Chem Inc., Elk Grove Village, IL, United States; cat. no. 80340), an enzymatic colorimetric method that avoids the overestimation associated with the conventional Jaffe reaction in rodent samples. An 8 μL serum sample was used per well and incubated for 5 min at 37 °C; absorbance was read at 550 nm within 15 min of adding the stop solution, using a Bio-Rad Model 680 microplate reader.
3. Ubiquitin C-terminal Hydrolase L1 (UCH-L1): Serum UCH-L1 was measured using a rat ELISA kit (MyBioSource Inc., San Diego, CA, United States; cat. no. MBS764271) according to the manufacturer’s protocol. A 100 μL serum sample was used per well and incubated for 15 min at 37 °C; absorbance was read at 450 nm within 15 min of adding the stop solution, using a Bio-Rad Model 680 microplate reader.
4. Glial Fibrillary Acidic Protein (GFAP): Serum GFAP was assessed using a high-sensitivity rat ELISA kit (Cloud-Clone Corp., Wuhan, China; cat. no. SEA068Ra) according to the manufacturer’s protocol. A 100 μL serum sample was used per well and incubated for 60 min at 37 °C; absorbance was read at 450 nm within 15 min of adding the stop solution, using a Bio-Rad Model 680 microplate reader. GFAP and UCH-L1 were included as neurotoxicity-related biomarkers to assess possible neural responses associated with fragrance exposure.
5. Arterial Blood Gases (ABG): Blood (0.25 mL) was collected from the carotid artery under ketamine anesthesia. Heparin (0.25 mL, 1000 IU/mL) was used during cannulation. PO_2_ and PCO_2_ were measured with the iSTAT EG7 cartridge while carefully avoiding air bubbles.
6. Heart Rate: Recorded in awake rats using ECG contact plates connected to a recorder (Vetterlein et al., 1984).
7. Blood Pressure: Measured by tail-cuff plethysmography based on pulse disappearance and reappearance during cuff inflation and deflation (Fritz and Rinaldi, 2008).

### Chemical analysis and product characterization

The tested fragrance formulation was characterized using Gas Chromatography-Mass Spectrometry (GC/MS; Trace GC 1310–ISQ, Thermo Scientific, Austin, TX, United States) with a TG-5MS capillary column (30 m × 0.25 mm × 0.25 μm). Components were identified by comparison with WILEY 09 and NIST 11 databases. Chemical characterization was performed to document the profile of the tested formulation and enhance reproducibility of the experimental conditions.

The tested fragrance formulation was a commercially available perfume sold under the trade name “Blue de Blue” (Milestone Perfumes, United Arab Emirates). The product was obtained in its original commercial packaging (100 mL; 3.4 FL.OZ) and used without dilution or chemical modification.

For inhalation exposure, 5 mL of the fragrance was applied onto a cotton piece and placed in the corner of an airtight exposure box equipped with a perforated lid. The cotton was positioned to prevent direct contact or licking by the animals. Each rat was individually exposed to the fragrance for 1 h daily and then returned to its home cage, according to a previously described exposure method (Ueno et al., 2019).

### Oxidative stress biomarkers

Heart, lung, and brain tissues were collected immediately after euthanasia, perfused with ice-cold PBS (pH 7.4) containing 0.16 mg/mL heparin to remove residual blood, and kept on ice throughout processing. Tissues were homogenized in cold buffer at a ratio of approximately 1 g tissue to 5–10 mL buffer, with the specific buffer composition following each assay kit’s protocol as detailed below. Homogenates were centrifuged at 4,000 rpm for 15 min at 4 °C, and the resulting supernatant was used for the assay. Samples not analyzed on the same day were stored at −80 °C. The following markers were measured using MyBioSource and Bio-diagnostic (Giza, Egypt) kits:

- TAC (cat. no. MBS2548432)
- MDA (MD2529)
- SOD (SD2521)
- GSH-Px (GP2524)
- CAT (CA2517)

All assays were performed according to manufacturers’ instructions.

### TAC measurement

Total antioxidant capacity (TAC) was determined using a commercial kit (MyBioSource, San Diego, CA, United States; cat. no. MBS2548432) based on the ABTS method. A peroxidase generates the radical cation ABTS•+, and antioxidants present in the sample suppress this reaction in proportion to their concentration; the resulting absorbance change was compared against a Trolox standard curve. Serum TAC was expressed as mmol Trolox equivalent/L, and tissue TAC was expressed as nmol Trolox equivalent/mg protein after normalization to tissue protein content.

### MDA measurement (TBARS method)

Malondialdehyde (MDA) was quantified using a commercial colorimetric kit (Bio-diagnostic, Giza, Egypt; cat. no. MD2529) based on the thiobarbituric acid reactive substances (TBARS) method of Ohkawa et al. Tissue was homogenized in cold 50 mM potassium phosphate buffer (pH 7.5), and the supernatant was reacted with a thiobarbituric acid chromogen in an acidic medium at 95 °C for 30 min. The resulting pink product was measured spectrophotometrically at 534 nm, and MDA levels in tissue were expressed as nmol/mg protein after normalization to tissue protein content; serum MDA was expressed as nmol/L.

### SOD measurement

Superoxide dismutase (SOD) activity was determined using a commercial colorimetric kit (Bio-diagnostic, Giza, Egypt; cat. no. SD2521), based on the method of Nishikimi et al. Tissue was homogenized in cold 100 mM potassium phosphate buffer (pH 7.0) containing 2 mM EDTA, and the supernatant was further treated with the kit’s extraction reagent. The assay relies on the ability of SOD to inhibit the phenazine methosulfate (PMS)-mediated reduction of nitroblue tetrazolium (NBT); absorbance was measured at 560 nm, and the percentage inhibition was used to calculate SOD activity, expressed as U/mg protein after normalization to tissue protein content.

### GSH-Px measurement

Glutathione peroxidase (GSH-Px) activity was determined using a commercial kit (Bio-diagnostic, Giza, Egypt; cat. no. GP2524), based on the coupled enzymatic method of Paglia and Valentine. Tissue was homogenized in cold 50 mM phosphate buffer (pH 7.0) containing 5 mM EDTA and 1 mM 2-mercaptoethanol. GSH-Px catalyzes the reduction of hydrogen peroxide using reduced glutathione (GSH); the resulting oxidized glutathione (GSSG) is recycled to GSH by glutathione reductase at the expense of NADPH. The rate of decrease in absorbance at 340 nm, reflecting NADPH consumption, is proportional to GSH-Px activity, expressed as U/mg protein after normalization to tissue protein content.

### CAT measurement

Catalase (CAT) activity was determined using a commercial colorimetric kit (Bio-diagnostic, Giza, Egypt; cat. no. CA2517), based on the method of Aebi. Tissue was homogenized in cold 50 mM potassium phosphate buffer (pH 7.4) containing 1 mM EDTA and 1 mL/L Triton X-100. Catalase in the sample reacts with a known quantity of hydrogen peroxide for a fixed time; the remaining hydrogen peroxide reacts, in the presence of peroxidase, with a chromogen to form a colored product measured at 510 nm, with color intensity inversely proportional to catalase activity. Results were expressed as U/mg protein after normalization to tissue protein content.

### Expression of results

Antioxidant enzyme activities (SOD, GSH-Px, CAT) in tissue homogenates were normalized to the protein content of each sample and expressed as U/mg protein. Total protein concentration was determined using the Bradford method (Bradford, 1976). Serum values were expressed per unit volume (µmol/mL for SOD and CAT; IU/L for GSH-Px) without protein normalization.

### Gene expression analysis (RT-PCR)

Real-time PCR (RT-PCR) was used to determine the relative expression of CYP1A1 and CYP1A2 in lung tissue, Ckmt2 in heart tissue, and KLF4 in brain tissue, using GAPDH as an internal reference gene, to assess transcriptional changes associated with fragrance exposure and vitamin E supplementation.

Total RNA was extracted from tissues using a Total RNA Purification Kit (Thermo Scientific, Fermentas; cat. no. K0731) according to the manufacturer’s protocol, and RNA concentration and purity were verified using a Q5000 UV-Vis spectrophotometer. Complementary DNA (cDNA) was synthesized using RevertAid H Minus Reverse Transcriptase (Thermo Scientific; cat. no. EP0451). Real-time PCR was performed using 2X Maxima SYBR Green/ROX qPCR Master Mix (Thermo Scientific; cat. no. K0221) and gene-specific primers (Table 1) on a StepOnePlus Real-Time PCR System (Applied Biosystems, Life Technologies, United States). Primers were designed with Primer-3 based on published rat sequences and checked for specificity using BLAST.

**TABLE 1:** Forward and reverse primer sequences, accession numbers, and product sizes (bp) used for real-time PCR.

Gene	Primer sequence (5/---3/)	Accession number	Product size (bp)
CYP1A-1	F/GGGCCAAGAGCTTCTTTGATG R/GTCCCGGATGTGGCCCTTCTCAAA	NM_012540.3	121
CYP1A-2	F/CTGCAGAAAACAGTCCAGGA	NM_012541.3	118
Ckmt2	R/GAGGGATGAGACCACCGTTGT F/CCCCCAAGTGCAGACTATCC R/TGGCATAGATGGTTGGGGTG	NM_001127652.1	79
KLF-4	F/GAGAGGAACTCTCTCACATGAAGC R/AAGGATAAAGTCTAGGTCCAGGAGA	NM_053713.1	185
GAPDH	F/CAACTCCCTCAAGATTGTCAGCAA R/GGCATGGACTGTGGTCATGA	NM_017008.4	117

Amplification was performed under the following cycling conditions: initial denaturation at 95 °C for 10 min, followed by 40 cycles of denaturation at 95 °C for 15 s, annealing at 60 °C for 30 s, and extension at 72 °C for 30 s. A melt curve analysis was performed immediately after amplification, from 60 °C to 95 °C, according to the default StepOnePlus software settings, to verify amplification specificity. Relative gene expression (fold change) was calculated using the 2^−ΔΔCT^ method (Livak and Schmittgen, 2001), with GAPDH as the reference gene. All reactions were performed with five biological replicates per group (n = 5 rats/group).

Table 1 lists the forward and reverse primer sequences, accession numbers, and product sizes (bp) for all genes analyzed.

### Histopathology

Tissues (lung, heart, brain) were fixed in 10% neutral buffered formalin (24–48 h), dehydrated through graded ethanol, cleared in xylene, and embedded in paraffin. Sections (4–5 µm) were cut using a rotary microtome, mounted on slides, deparaffinized, rehydrated, and stained with hematoxylin and eosin (H&E) (Bancro et al., 1996). Sections were examined for histopathological changes.

### Assay validation and data standardization

To ensure reliability and reproducibility, all biochemical and molecular assays were performed according to the manufacturers’ protocols. Commercial ELISA and oxidative stress assay kits included standard curves, blanks, and calibration procedures where applicable. Data standardization procedures were applied to minimize technical variability and improve consistency across experimental measurements.

### Statistical analysis

Data were tested for normality using the Shapiro–Wilk test. Normally distributed data were analyzed using one-way analysis of variance (ANOVA) followed by Tukey’s multiple comparison test. For datasets that did not meet the assumption of normality, the Kruskal–Wallis test was applied, followed by Dunn’s multiple comparison test when appropriate. Results are presented as mean ± standard error (SE). Statistical significance was set at p ≤ 0.05. All analyses were performed using SPSS version 26 (IBM Corp., Armonk, NY, United States).

## Results

All results are expressed as mean ± S.E. (n = 5), with statistical significance defined at p ≤ 0.05.

### Gas chromatography-mass spectroscopy (GC-MS) analysis of the tested fragrance formulation

**FIGURE 2:**
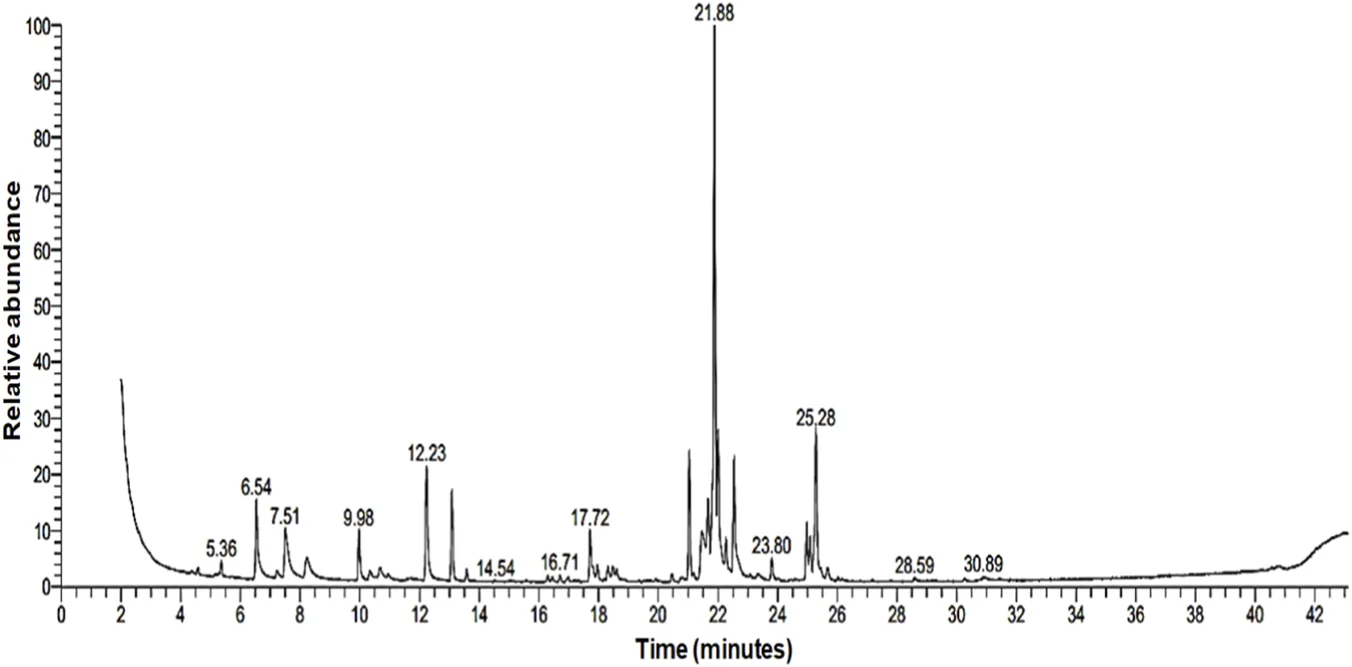
GC-MS chromatogram of the tested fragrance formulation showing the main peaks at specific retention times. The identified compounds corresponding to these peaks are listed in Table 2.

GC–MS analysis of the tested fragrance formulation identified multiple volatile compounds, including monoterpenes, sesquiterpenes, and oxygenated derivatives. The most abundant component accounted for about 26.9% of the total peak area (RT ≈ 21.9 min), followed by other notable compounds at RT 25.3 and 21.0 min (Table 2; Figure 2). These data confirm that the tested product was a chemically complex fragrance mixture rather than a single isolated compound.

**TABLE 2:** GC-MS analysis of the tested fragrance formulation showing retention time, identified compounds, molecular formula, molecular weight, library match score, and peak area percentage.

No.	RT (min)	Name	M. F.	Molecular weight (g/mol)	Library match score	Peak area %
1	6.53	Cyclohexanol, 1 -methyl-4-(1-methylethenyl)-acetate	C_10_H_16_	136.24	912	4.09
2	7.50	7-Octen-2-ol, 2,6-dimethyl	C_10_H_20_O	156.27	897	3.17
3	8.23	1,6-Octadien-3-Ol, 3,7-Dimethyl	C_10_H_18_O	154.25	887	1.66
4	9.97	2-Hexene, 6,6-dimethoxy-2,5,5-trimethyl	C_11_H_22_O_2_	186.29	898	2.59
5	12.23	1,5-Dimethyl-1-vinyl-4-hexenyl 2-aminobenzoate	C_17_H_23_NO_2_	273.38	949	6.11
6	13.08	4-Tert-Butylcyclohexyl acetate	C_12_H_22_O	182.31	198	4.54
7	17.72	3-Methyl-4-(2,6,6-trimethyl-2 -cyclohexen-1-yl)-3-buten-2 one	C_14_H_22_O	206.33	920	2.47
8	21.04	Epicedrol	C_15_H_26_O	222.37	786	6.42
9	21.45	Cyclopentaneacetic acid, 3-oxo-2-pentyl-, methyl ester	C_13_H_22_O_3_	226.32	848	2.94
10	21.67	1-Naphthalenemethanol, 1,4,4a,5,6,7,8,8a-octahydro 2,5,5,8a-tetramethy	C_15_H_26_O	222.37	760	2.81
11	21.88	7a-Isopropenyl-4,5-dimethyloctahyd roinden-4-yl) methanol	C_15_H_22_O_2_	234.34	794	26.87
12	22.00	(1E)-1-(2,6,6-Trimethyl-1-cyclohexen-1-yl)-1-penten-3-one	C_14_H_22_O	206.33	791	4.92
13	22.27	2-(4a,8-Dimethyl-6-oxo-1,2,3,4,4a,5, 6,8a-octahydro naphthalen-2-yl)-propionaldehyde	C_15_H_22_O	218.34	764	1.33
14	22.53	3-Methyl-4-(2,6,6-trimethyl-1 -cyclohexen-1-yl)-3-buten-2 one	C_14_H_22_O	206.33	793	7.43
15	23.80	Ambrox	C_16_H_28_O	236.40	862	1.19
16	24.97	9,12-Octadecadienoic acid (Z,Z)	C_18_H_32_O2	280.45	871	2.92
17	25.08	Oxacycloheptadec-8-en-2-one, (8Z)-	C_16_H_28_O_2_	252.40	856	2.07
18	25.28	Oxacyclotetradecane-2,11-dione, 13-methyl	C_14_H_24_O_3_	240.34	916	8.08

### Serum creatinine and cardiac troponin T (cTnT)

Exposure to the tested fragrance formulation significantly elevated serum creatinine and troponin compared with controls. Vitamin E pretreatment reduced these elevations but did not normalize them completely (Table 3). The increase in troponin suggests cardiac stress, while the increase in creatinine indicates altered renal-related biochemical status under the present experimental conditions.

**TABLE 3:** Effect of exposure to the tested fragrance formulation on serum creatinine and troponin levels.

Groups	Creatinine (mg/dl)	Cardiac troponin T (cTnT) (ng/ml)
G1	0.60 ± 0.095^b^	2.064 ± 0.328^b^
G2	0.58 ± 0.092^b^	1.722 ± 0.277^b^
G3	0.52 ± 0.089^b^	1.682 ± 0.380^b^
G4	0.99 ± 0.130	3.412 ± 1.208
G5	0.79 ± 0.153	2.75 ± 0.861

Values are presented as mean ± SE (n = 5). Different superscript letters indicate statistically significant differences among groups at p ≤ 0.05. Groups sharing at least one letter are not significantly different.

### Arterial blood gases

Exposure to the tested fragrance formulation decreased PO_2_ and PCO_2_ values compared with controls. Vitamin E supplementation partially improved these levels (Table 4). These changes suggest an effect on respiratory function and gas exchange, which is consistent with the lung histological alterations described below.

**TABLE 4:** Effect of exposure to the tested fragrance formulation on arterial blood gases (ABG).

Groups	PO2 (mmHg)	PCo2 (mmHg)
G1	95.58 ± 1.46	47.8 ± 1.35^b^
G2	93.97 ± 1.52^b^	45.7 ± 2.04^b^
G3	96.41 ± 2.45^b^	48.2 ± 1.45^b^
G4	88.39 ± 2.98^a^	39.1 ± 2.50
G5	91.06 ± 1.70^a^	42.9 ± 1.28^a,b^

Values are presented as mean ± SE (n = 5). Different superscript letters indicate statistically significant differences among groups at p ≤ 0.05. Groups sharing at least one letter are not significantly different.

### GFAP and UCH-L1

GFAP and UCH-L1 were assessed as neurotoxicity-related markers. No significant differences were observed in serum GFAP or UCH-L1 levels among groups (Table 5). However, the absence of significant serum changes does not exclude localized histological or molecular alterations in brain tissue, as KLF4 expression and cerebral cortex histology showed exposure-related changes.

**TABLE 5:** Effect of exposure to the tested fragrance formulation on serum GFAP and UCH-L1 levels.

Groups	GFAP (pg/ml)	UCH-L1 (pg/ml)
G1	358.6 ± 20	2,801 ± 761.1
G2	371.8 ± 18.7	2,961 ± 718
G3	361.1 ± 20.1	2,739.6 ± 268.2
G4	389.6 ± 15.1	3,079.8 ± 686.1
G5	373.9 ± 12.9	2,923 ± 498.3

Values are presented as mean ± SE (n = 5). Different superscript letters indicate statistically significant differences among groups at p ≤ 0.05. Groups sharing at least one letter are not significantly different.

### Blood pressure and heart rate

The tested fragrance-exposed group showed significantly higher blood pressure and heart rate compared with controls. Vitamin E supplementation attenuated but did not fully prevent these increases (Table 6). These physiological changes align with the observed increase in troponin and Ckmt2 expression, supporting a cardiovascular stress response in the exposed group.

**TABLE 6:** Effect of exposure to the tested fragrance formulation on blood pressure (BP) and heart rate (HR) in rats.

Groups	BP (mmHg)	HR (bpm)
G1	112 ± 2.91^b^	352.4 ± 7.83^b^
G2	110.4 ± 4.16^b^	331.8 ± 5.63^a,b^
G3	111 ± 3.39b	329 ± 5.24^a,b^
G4	130.4 ± 4.39	375.8 ± 7.98
G5	114.6 ± 4.16^b^	362.8 ± 7.46^b^

Values are presented as mean ± SE (n = 5). Different superscript letters indicate statistically significant differences among groups at p ≤ 0.05. Groups sharing at least one letter are not significantly different.

### Oxidative stress parameters

Exposure to the tested fragrance formulation reduced antioxidant defenses (TAC, SOD, CAT, and GSH-Px) and increased MDA levels, particularly in serum and lung tissues. These biochemical changes indicate oxidative imbalance and lipid peroxidation. The stronger response in serum and lung tissues is consistent with inhalation as the route of exposure and with the lung histopathological findings described below. Heart and brain tissues were less affected biochemically, although molecular and histological changes were still observed. Vitamin E treatment improved several antioxidant markers and reduced MDA levels, indicating partial protection against oxidative injury (Tables 7–11).

**TABLE 7:** Effect of exposure to the tested fragrance formulation on total antioxidant capacity (TAC) in serum, lung, heart, and brain tissues. Units differ by sample type (see column headers).

Groups	Serum (mmol Trolox equiv./L)	Lung Trolox equiv./mg protein)	Heart Trolox equiv./mg protein)	Brain (nmol Trolox equiv./mg protein)
G1	2.66 ± 0.22	27.31 ± 2.73	3.21 ± 0.47	6.36 ± 0.45
G2	2.94 ± 0.42b	32.27 ± 4.48^b^	3.48 ± 0.33	6.52 ± 0.64
G3	3.40 ± 0.50^a,b^	39.55 ± 1.62^b^	3.55 ± 0.46	6.45 ± 0.51
G4	2.00 ± 0.24^a^	20.23 ± 2.88^a^	3.14 ± 0.45	6.22 ± 0.42
G5	2.41 ± 0.30 25	20 ± 2.37^a,b^	3.38 ± 0.45	6.31 ± 0.50

Values are presented as mean ± SE (n = 5). Different superscript letters indicate statistically significant differences among groups at p ≤ 0.05. Groups sharing at least one letter are not significantly different.

**TABLE 8:** Effect of exposure to the tested fragrance formulation on malondialdehyde (MDA) levels in serum, lung, heart, and brain tissues. Units differ by sample type (see column headers).

Groups	Serum (nmol/L)	Lung (nmol/mg protein)	Heart (nmol/mg protein)	Brain (nmol/mg protein)
G1	32.34 ± 3.35	0.57 ± 0.07	0.14 ± 0.03	8.08 ± 0.70
G2	30.85 ± 2.19^b^	0.47 ± 0.09^b^	0.13 ± 0.04	7.90 ± 0.96
G3	30.15 ± 3.87^a,b^	0.41 ± 0.06^a,b^	0.13 ± 0.03	7.80 ± 0.97
G4	48.69 ± 3.29^a^	1.01 ± 0.23^a^	0.19 ± 0.05	8.44 ± 0.96
G5	32.34 ± 3.35	0.73 ± 0.08^a,b^	0.13 ± 0.05	8.07 ± 0.76

Values are presented as mean ± SE (n = 5). Different superscript letters indicate statistically significant differences among groups at p ≤ 0.05. Groups sharing at least one letter are not significantly different.

**TABLE 9:** Effect of exposure to the tested fragrance formulation on superoxide dismutase (SOD) levels in serum, lung, heart, and brain tissues. Units differ by sample type (see column headers).

Groups	Serum (µmol/mL)	Lung (Unit/mg protein)	Heart (Unit/mg protein)	Brain (Unit/mg protein)
G1	3.28 ± 0.51^b^	2.41 ± 0.37	19.05 ± 2.67	3.79 ± 0.82
G2	3.70 ± 0.46^b^	2.98 ± 0.11^a,b^	18.93 ± 1.78	3.59 ± 0.84
G3	3.88 ± 0.95^b^	2.99 ± 0.34b	19.24 ± 2.92	3.66 ± 1.09
G4	2.15 ± 0.23	1.81 ± 0.21	16.46 ± 2.04	3.16 ± 0.44
G5	2.72 ± 0.36	2.00 ± 0.17	18.77 ± 3.39	3.57 ± 0.81

Values are presented as mean ± SE (n = 5). Different superscript letters indicate statistically significant differences among groups at p ≤ 0.05. Groups sharing at least one letter are not significantly different.

**TABLE 10:** Effect of exposure to the tested fragrance formulation on glutathione peroxidase (GSH-Px) levels in serum, lung, heart, and brain tissues. Units differ by sample type (see column headers).

Groups	Serum (IU/L)	Lung (Unit/mg protein)	Heart (Unit/mg protein)	Brain (Unit/mg protein)
G1	54.47 ± 3.82	37.08 ± 4.01	6.68 ± 0.61	0.28 ± 0.05
G2	58.76 ± 5.13^b^	40.29 ± 3.10^b^	6.83 ± 0.59^b^	0.29 ± 0.05
G3	59.43 ± 5.52^b^	41.18 ± 4.90^b^	7.13 ± 0.75^b^	0.30 ± 0.03
G4	48.63 ± 5.25	29.23 ± 3.48^a^	5.93 ± 0.43^b^	0.24 ± 0.03
G5	51.45 ± 3.13	30.02 ± 4.74^a^	6.49 ± 0.47	0.26 ± 0.04^b^

Values are presented as mean ± SE (n = 5). Different superscript letters indicate statistically significant differences among groups at p ≤ 0.05. Groups sharing at least one letter are not significantly different.

**TABLE 11:** Effect of exposure to the tested fragrance formulation on catalase (CAT) levels in serum, lung, heart, and brain tissues. Units differ by sample type (see column headers).

Groups	Serum (µmol/mL)	Lung (Unit/mg protein)	Heart (Unit/mg protein)	Brain (Unit/mg protein)
G1	32.51 ± 4.88	28.56 ± 3.42	5.00 ± 0.76	4.53 ± 0.76
G2	39.62 ± 4.41^b^	34.91 ± 3.39^a,b^	5.37 ± 0.82	4.82 ± 0.74
G3	42.46 ± 5.91^a,b^	39.82 ± 2.39^a,b^	5.81 ± 0.96	4.86 ± 0.35
G4	23.23 ± 4.36^a^	21.87 ± 3.25^a^	4.24 ± 0.57	4.14 ± 0.50
G5	27.06 ± 3.08	26.76 ± 3.18	4.75 ± 0.66	4.59 ± 0.39

Values are presented as mean ± SE (n = 5). Different superscript letters indicate statistically significant differences among groups at p ≤ 0.05. Groups sharing at least one letter are not significantly different.

### Gene expression

**FIGURE 3:**
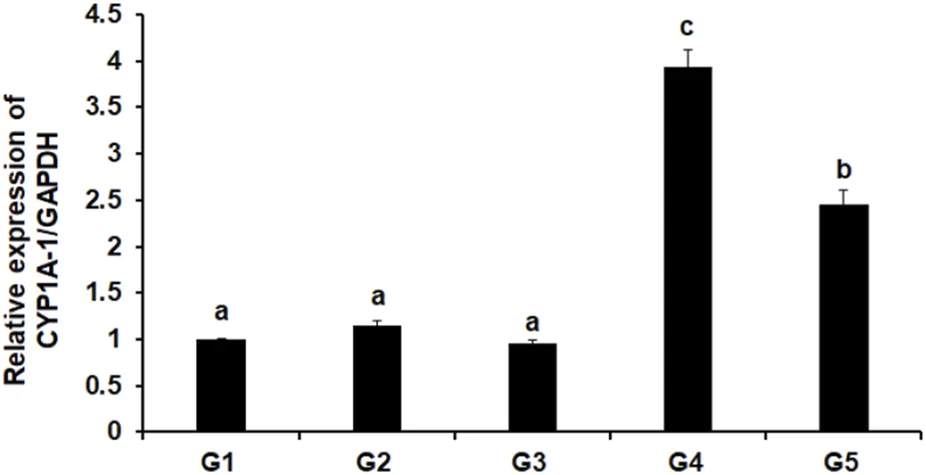
Relative expression of CYP1A1 in lung tissues of rats exposed to the tested fragrance formulation. Values are presented as mean ± SE (n = 5). Different superscript letters indicate statistically significant differences among groups at p ≤ 0.05. Groups sharing at least one letter are not significantly different.

**FIGURE 4:**
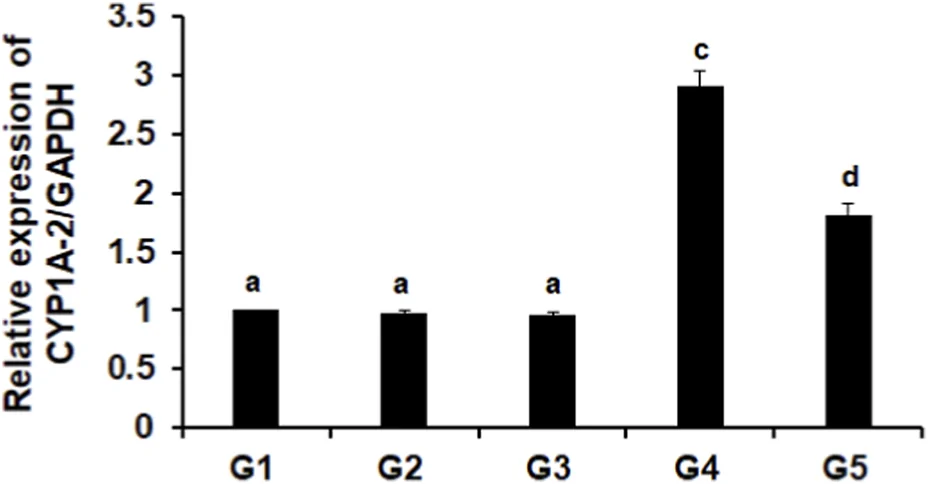
Relative expression of CYP1A2 in lung tissues of rats exposed to the tested fragrance formulation. Values are presented as mean ± SE (n = 5). Different superscript letters indicate statistically significant differences among groups at p ≤ 0.05. Groups sharing at least one letter are not significantly different.

**FIGURE 5:**
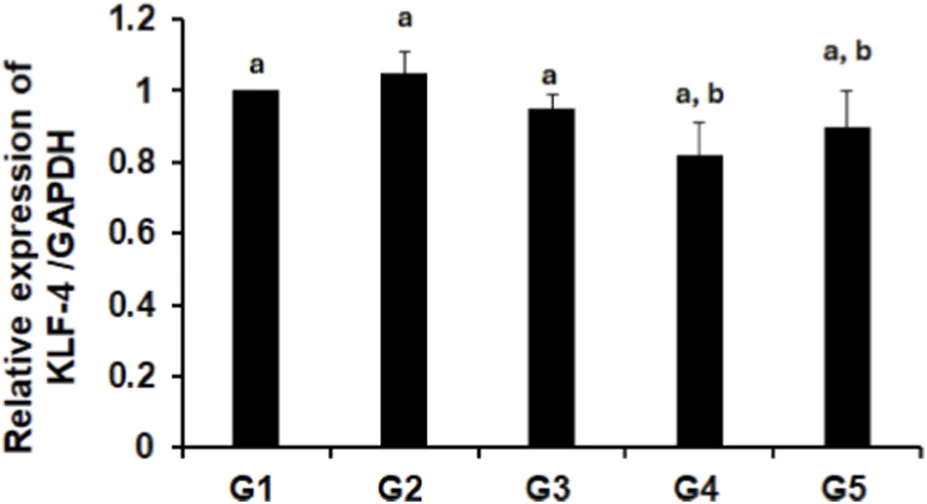
Relative expression of KLF4 in brain tissues of rats exposed to the tested fragrance formulation. Values are presented as mean ± SE (n = 5). Different superscript letters indicate statistically significant differences among groups at p ≤ 0.05. Groups sharing at least one letter are not significantly different.

**FIGURE 6:**
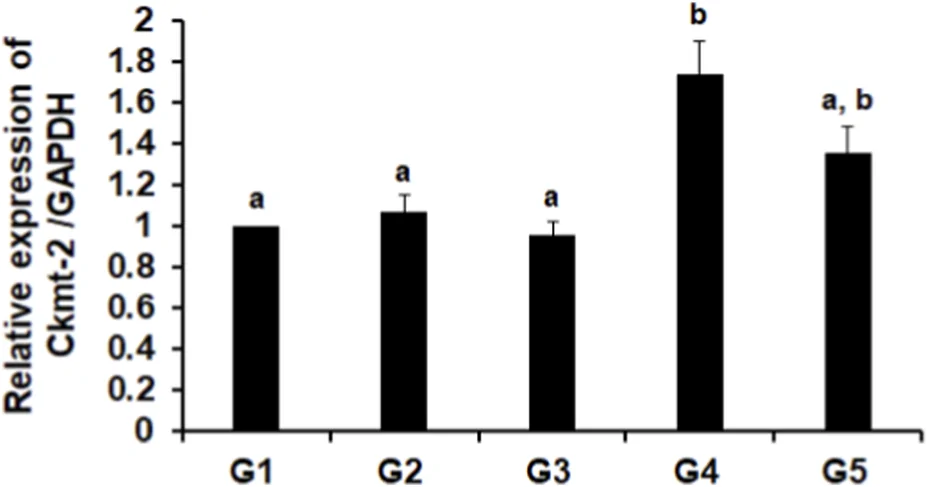
Relative expression of Ckmt2 in heart tissues of rats exposed to the tested fragrance formulation. Values are presented as mean ± SE (n = 5). Different superscript letters indicate statistically significant differences among groups at p ≤ 0.05. Groups sharing at least one letter are not significantly different.

RT-PCR analysis revealed upregulation of CYP1A1 and CYP1A2 in lung tissues and Ckmt2 in heart tissue, while KLF4 expression in brain tissue showed a tendency toward reduction in the tested fragrance-exposed group, although the difference did not reach statistical significance. Vitamin E pretreatment partially normalized these expression patterns. Relative expression was calculated using the 2^−ΔΔCt^ method (Livak and Schmittgen, 2001) (Figures 3–6). These molecular findings support the biochemical and histological observations by indicating xenobiotic-related responses in lung tissue, cardiac stress-related changes in heart tissue, and altered neuroprotective signaling in brain tissue.

#### CYP1A-1

#### CYP1A-2

#### KLF-4

#### Ckmt2

### Histology

**FIGURE 7:**
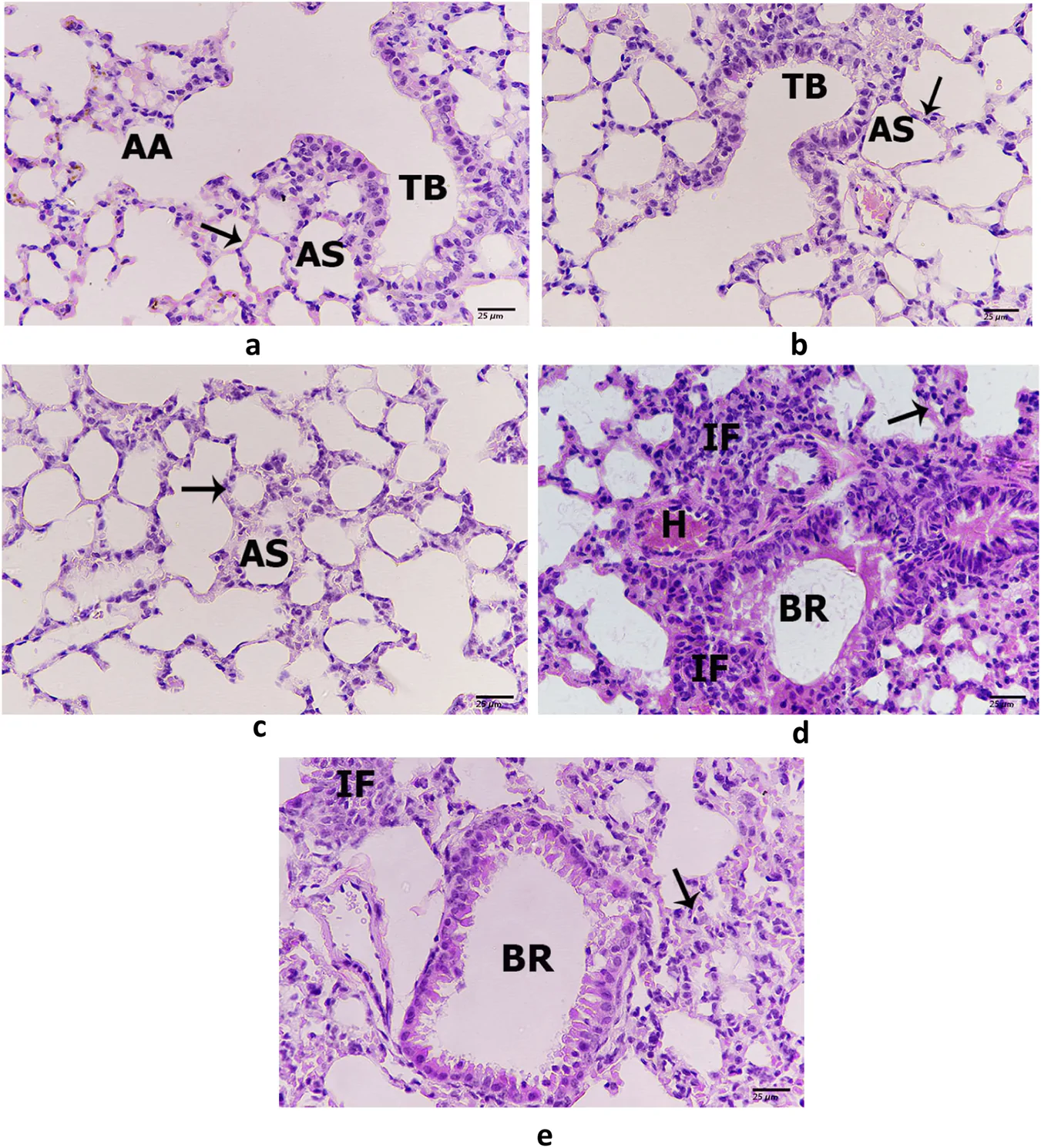
**(a–e)** Histopathological changes in lung tissues among experimental groups. **(a)** Photomicrograph of lung (G1, control) showing normal architecture: terminal bronchiole (TB), alveolar sac (AS), alveolar airway (AA), and interalveolar septum (arrow). (Hematoxylin & Eosin [H&E], ×400) **(b)** Photomicrograph of lung (G2, olive oil) showing normal architecture with terminal bronchiole (TB), alveolar sac (AS), and interalveolar septum (arrow). (H&E, ×400) **(c)** Photomicrograph of lung (G3, vitamin E/olive oil) showing normal architecture with alveolar sac (AS) and interalveolar septum (arrow). (H&E, ×00) **(d)** Photomicrograph of lung (G4, odor exposed) showing severe leukocytic infiltrative exudate (IF), hemorrhage-filled sacs (H), bronchiole filled with matrix (BR), and thickened interalveolar septa (arrow). (H&E, ×400) **(e)** Photomicrograph of lung (G5, odor + vitamin E) showing reduced leukocytic infiltrative exudate (IF), improved bronchiole (BR), and thickened interalveolar septa (arrow). (H&E, ×400).

Histological examination supported the biochemical and molecular findings. In the lung, exposure to the tested fragrance formulation induced leukocytic infiltration, alveolar hemorrhage, bronchiolar changes, and thickened interalveolar septa. These tissue alterations were consistent with the reduction in PO_2_ and the oxidative stress changes observed in lung tissue. Vitamin E treatment reduced the severity of leukocytic infiltration and improved bronchiolar architecture, although mild septal thickening remained (Figures 7a–e).

**FIGURE 8:**
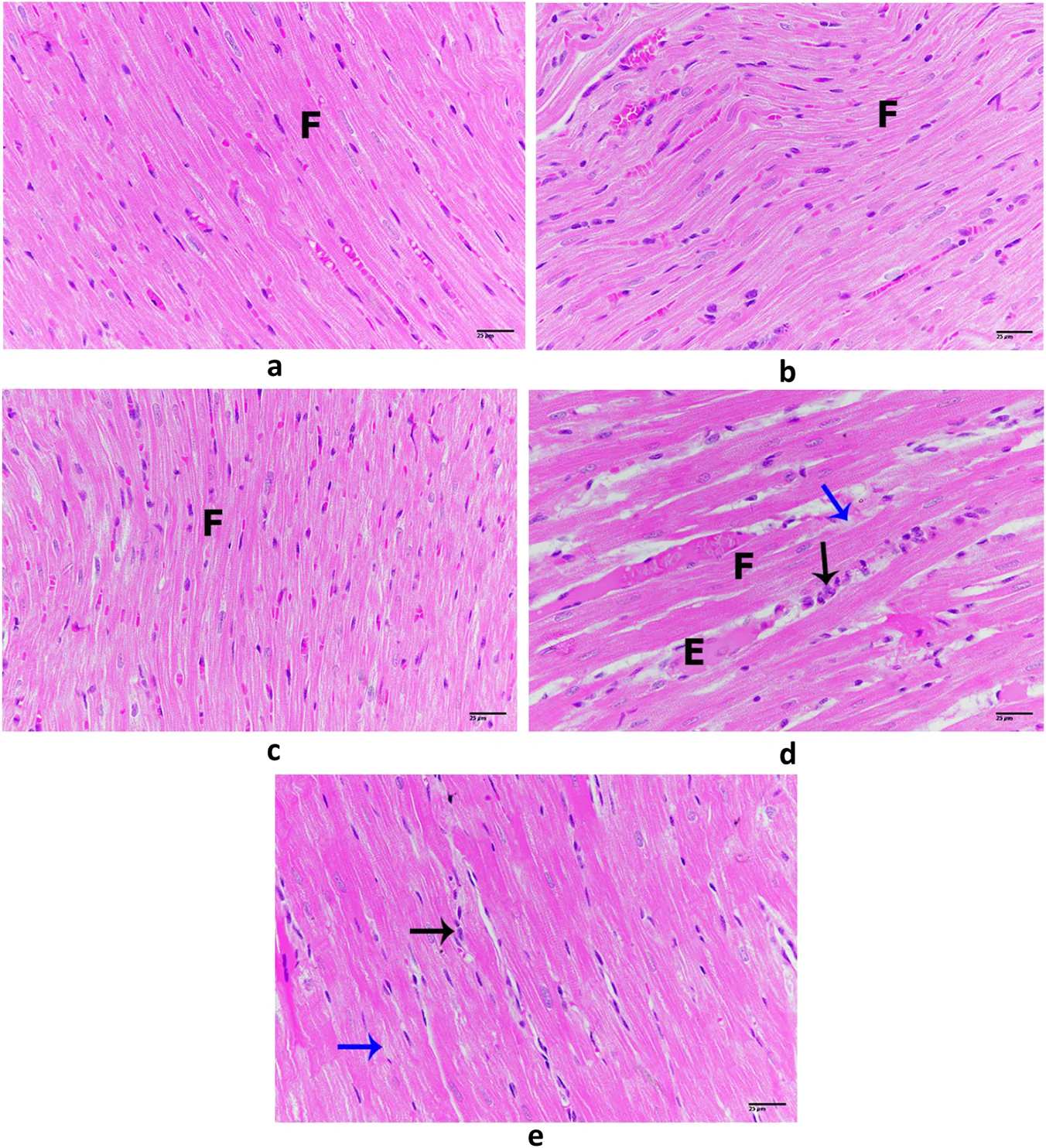
**(a–e)** Histopathological changes in heart tissues among experimental groups. **(a)** Photomicrograph of cardiac muscle (G1, control) showing normal myocardial fibers (F) with central nuclei. (H&E, ×400) **(b)** Photomicrograph of cardiac muscle (G2, olive oil) showing normal myocardial fibers (F) with central nuclei. (H&E, ×400) **(c)** Photomicrograph of cardiac muscle (G3, vitamin E/olive oil) showing normal myocardial fibers (F) with central nuclei. (H&E, ×400) **(d)** Photomicrograph of cardiac muscle (G4, odor-exposed) showing degeneration of myocardial fibers (blue arrow), aggregation of infiltrative cells in the interspaces (black arrow), and interstitial edema (E). (H&E, ×400) **(e)** Photomicrograph of cardiac muscle (G5, odor + vitamin E) showing less degeneration of myocardial fibers (blue arrow) and smaller aggregation of infiltrative cells in the interspaces (black arrow). (H&E, ×400).

In cardiac muscle, exposure to the tested fragrance formulation caused myocardial fiber degeneration, widened interspaces, infiltrative cell aggregation, and interstitial edema. These histological findings were consistent with elevated serum troponin, increased heart rate and blood pressure, and upregulated Ckmt2 expression. Vitamin E treatment reduced myocardial degeneration and inflammatory cell aggregation, indicating partial cardioprotective effects (Figures 8a–e).

**FIGURE 9:**
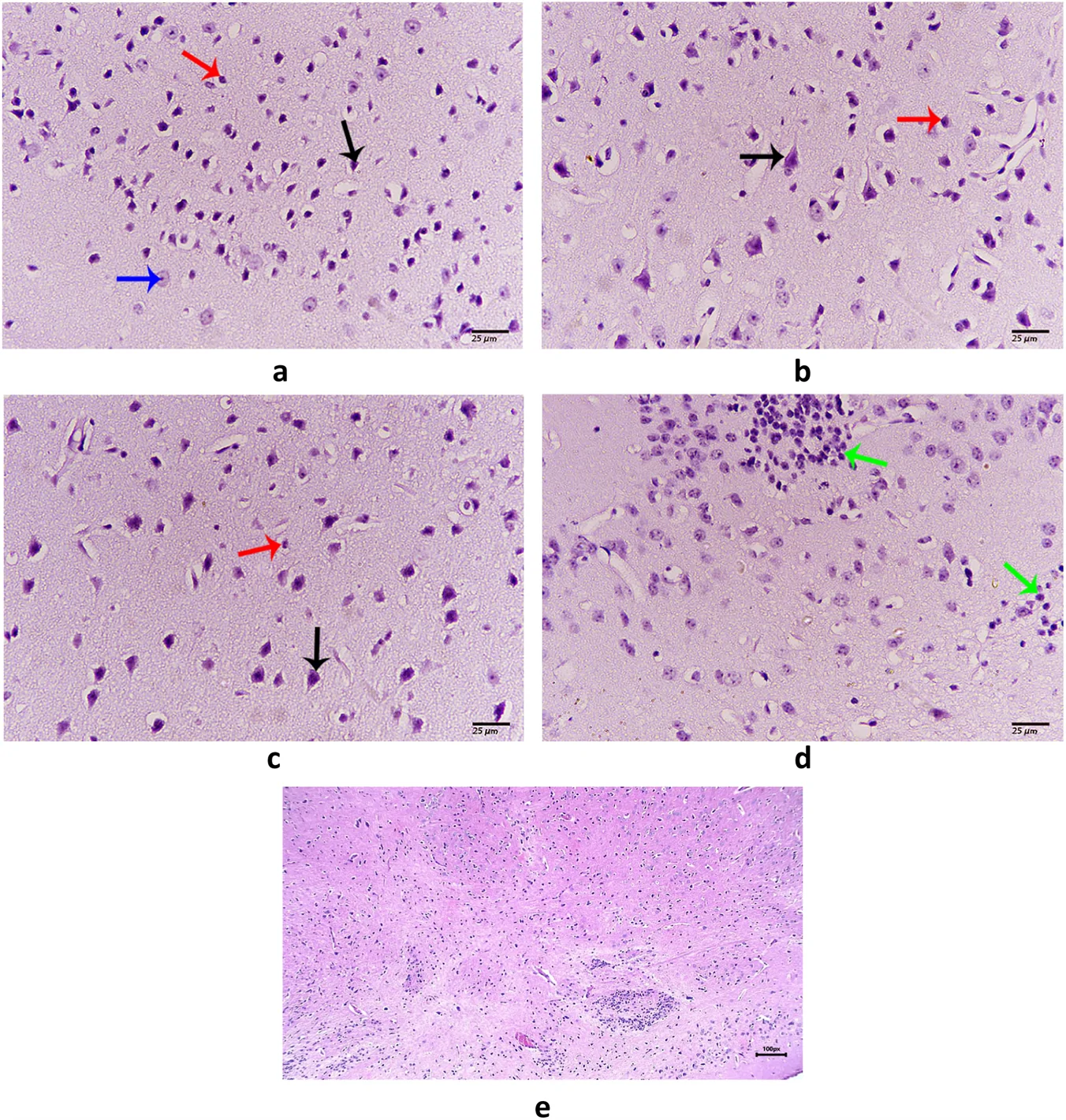
**(a–e)** Histopathological changes in cerebral cortex tissues among experimental groups. **(a)** Photomicrograph of cerebral cortex (G1, control) showing normal structure: pyramidal neuron (black arrow), granular neuron (blue arrow), and glial cell (red arrow). (H&E, ×400) **(b)** Photomicrograph of cerebral cortex (G2, olive oil) showing normal structure with pyramidal neuron (black arrow) and glial cell (red arrow). (H&E, ×400) **(c)** Photomicrograph of cerebral cortex (G3, vitamin E/olive oil) showing normal structure with pyramidal neuron (black arrow) and glial cell (red arrow). (H&E, ×400) **(d)** Photomicrograph of cerebral cortex (G4, odor-exposed) showing severe gliosis (green arrow). (H&E, ×400) **(e)** Photomicrograph of cerebral cortex (G5, odor + vitamin E) showing partial improvement with reduced gliosis (green arrow) and limited focal inflammatory cell infiltration compared with the odor-exposed group (G4). Hematoxylin and eosin stain (H&E). Scale bar = 100 μm.

In the cerebral cortex, exposure to the tested fragrance formulation caused severe gliosis. Although Klf4 expression showed a tendency toward reduction, the difference did not reach statistical significance. Together, these findings suggest localized neural tissue responses under the present experimental conditions. Although serum GFAP and UCH-L1 did not differ significantly among groups, the histological and gene expression findings indicate localized brain tissue responses. Vitamin E treatment reduced gliosis, suggesting partial neuroprotective effects under the present experimental conditions (Figures 9a–e).

## Discussion

### Overall integration of biochemical, molecular, and histological findings

The biochemical, molecular, and histological findings showed a consistent pattern of multi-organ response to the tested fragrance formulation. In the lung, reduced antioxidant defenses, increased lipid peroxidation, induction of CYP1A1 and CYP1A2 expression, reduced PO_2_, and inflammatory histological changes supported respiratory tissue involvement. In the heart, elevated troponin, increased blood pressure and heart rate, upregulated Ckmt2 expression, and myocardial histological damage supported cardiovascular stress. In the brain, cortical gliosis and a non-significant trend toward reduced Klf4 expression suggested localized neural tissue responses despite the absence of significant serum GFAP and UCH-L1 changes. Across these organs, vitamin E partially improved oxidative, molecular, and histological alterations but did not fully restore normal findings.

### Cardiovascular effects

Exposure to the tested fragrance formulation in this study was associated with cardiovascular stress. Elevated troponin levels, together with increased blood pressure and heart rate, suggest myocardial injury and membrane damage associated with oxidative imbalance (Xiao et al., 2021; Lee et al., 2021). Previous studies have shown that volatile essential oils can alter cardiovascular function not only through oxidative stress but also through disturbances in autonomic regulation (Lee et al., 2022; Sadgrove et al., 2021). Increased sympathetic activity and catecholamine release following inhalation exposure may explain the observed elevations in blood pressure and heart rate (Smith et al., 1999). Similar findings have been reported in humans, where inhalation of grapefruit essential oil increased mean arterial pressure and sympathetic nerve activity (Kawai et al., 2025; Kawai et al., 2020).

In addition, increased expression of Ckmt2, a mitochondrial creatine kinase gene associated with cardiac stress, was observed in the present study (Adefolaju et al., 2016). This molecular finding, together with elevated troponin and myocardial histological changes including degeneration and inflammatory cell infiltration, supports the involvement of oxidative and metabolic pathways in cardiac tissue injury. Vitamin E supplementation reduced troponin levels, blood pressure, heart rate, and histological damage, indicating partial preservation of cardiomyocyte integrity (Xiao et al., 2021; Boshtam et al., 2002). However, incomplete recovery suggests that oxidative stress alone may not fully explain the cardiovascular responses observed after exposure.

### Respiratory effects

Exposure to the tested fragrance formulation induced marked histopathological alterations in lung tissue, including leukocytic infiltration, alveolar hemorrhage, and septal thickening, which are indicative of inflammatory injury (Gabriel-Brisibe et al., 2020; Wang et al., 2013). These findings are consistent with previous reports linking volatile aromatic compounds to airway inflammation and oxidative stress (Gabriel-Brisibe et al., 2020; Wang et al., 2013; Andrade et al., 2014). Vitamin E treatment reduced tissue injury and improved several biochemical parameters, indicating partial protective effects (La Torre et al., 2021; Ungurianu et al., 2021).

Arterial blood gas analysis further supported these observations. Reduced PO_2_ values in exposed animals are consistent with impaired pulmonary function (Woodrow, 2004). The observed reduction in PCO_2_ may reflect altered ventilation patterns and/or impaired gas exchange. Because blood pH was not measured, the precise physiological mechanism underlying the decrease in PCO_2_ cannot be definitively determined. Partial recovery after vitamin E treatment suggests improvement in alveolar function, although complete normalization was not achieved (Abdoulhossein et al., 2018).

At the molecular level, exposure to the tested fragrance formulation increased CYP1A1 and CYP1A2 expression in lung tissue (Takemoto et al., 2020; de Sousa et al., 2023). These enzymes are key components of phase I xenobiotic metabolism and are commonly induced following exposure to inhaled environmental chemicals and volatile organic compounds. Their upregulation may reflect an adaptive response to increased metabolic demand for detoxification. However, enhanced CYP activity can also generate reactive intermediates capable of promoting oxidative stress and inflammatory injury (de Sousa et al., 2023). The concurrent increase in CYP1A1 and CYP1A2 expression, together with reduced antioxidant defenses, elevated lipid peroxidation, and histological lung injury, supports the involvement of xenobiotic-induced oxidative mechanisms in the pulmonary response observed in the present study. Vitamin E reduced this upregulation but did not restore baseline expression, indicating partial modulation of metabolic stress pathways rather than complete prevention of the underlying biological response (La Torre et al., 2021).

### Neurological effects

Exposure to the tested fragrance formulation also affected neural tissue. Histological analysis demonstrated gliosis within the cerebral cortex, indicating astrocytic activation and neuroinflammatory responses (Ungurianu et al., 2021). Similar observations have been reported following exposure to volatile organic compounds capable of crossing the blood–brain barrier (Sadgrove et al., 2021; de Sousa et al., 2023). Vitamin E treatment reduced the severity of gliosis but did not completely eliminate these changes, consistent with its antioxidant and anti-inflammatory properties (La Torre et al., 2021).

Brain histology revealed cortical gliosis in the exposed group, whereas KLF4 expression showed a non-significant downward trend. These findings suggest that histological alterations may occur in the absence of significant changes in KLF4 expression under the present experimental conditions. Further studies are needed to clarify the relationship between cortical gliosis and KLF4 regulation.

Interestingly, serum GFAP and UCH-L1 levels did not show significant changes despite histological and molecular alterations in brain tissue. This finding suggests that localized tissue responses may occur before detectable systemic changes appear in circulating neurotoxicity-related biomarkers.

### Oxidative stress and antioxidant imbalance

Exposure to the tested fragrance formulation disrupted redox homeostasis, as reflected by increased MDA levels and reduced TAC, SOD, CAT, and GSH-Px activities. These findings indicate that reactive oxygen species generation exceeded endogenous antioxidant defenses, promoting lipid peroxidation and cellular injury (de Sousa et al., 2023; Gabriel-Brisibe et al., 2020; Wang et al., 2013; Arivazhagan et al., 2000; Chi et al., 2015; Airaodion et al., 2020).

The biochemical findings were consistent with tissue-level observations. Lung tissue demonstrated greater biochemical alterations and histological injury compared with heart and brain tissues, which may reflect the inhalational route of exposure and direct contact with respiratory tissue. Similar findings have been reported following exposure to air fresheners and essential oils, supporting the role of volatile compounds in oxidative injury (Xiao et al., 2021; Lee et al., 2021; Andrade et al., 2014).

Vitamin E supplementation attenuated these changes by reducing lipid peroxidation and partially restoring antioxidant enzyme activities. These findings support its role as an exogenous antioxidant capable of reinforcing endogenous defense mechanisms. Nevertheless, incomplete normalization suggests either insufficient antioxidant capacity under continuous exposure conditions or involvement of additional non-oxidative pathways.

### Limitations and future directions

This study focused on oxidative stress, physiological responses, histological alterations, and selected gene expression markers (CYP1A1, CYP1A2, Ckmt2, and KLF4). Neurotoxicity-related serum biomarkers (GFAP and UCH-L1) were included; however, they did not show significant differences among groups. Therefore, neurological interpretation relied primarily on histological findings, while KLF4 expression was considered supportive but not statistically significant.

Additional apoptotic and inflammatory markers such as Bax, Bcl-2, caspase-3, and NF-κB were not included in the present work. Future studies incorporating these markers may provide further mechanistic information and stronger biological validation.

In addition, the use of a single tested fragrance formulation, one exposure concentration, one vitamin E dose, and a limited sample size restricts dose–response interpretation and limits extrapolation to other fragrance products. Future investigations should include larger sample sizes, multiple formulations, component-specific analysis, and different vitamin E doses.

Future studies should also evaluate additional antioxidant compounds and combined antioxidant strategies to determine whether greater protection can be achieved against fragrance-induced oxidative stress and tissue injury. Comparative evaluation of different antioxidant approaches may help identify more effective interventions and clarify the relative contribution of oxidative mechanisms to fragrance-associated toxicity.

## Conclusion

Repeated inhalation exposure to the tested commercial fragrance formulation produced measurable multi-organ biological effects in rats, including oxidative stress, impairment of antioxidant defense systems, cardiovascular and respiratory alterations, histopathological injury, and changes in selected molecular markers. The observed upregulation of CYP1A1, CYP1A2, and Ckmt2, together with the histological and biochemical findings, supports the involvement of oxidative and xenobiotic-related pathways in the biological response to the tested fragrance formulation. Although cortical gliosis was observed in brain tissue, the reduction in KLF4 expression did not reach statistical significance and should therefore be interpreted with caution.

Vitamin E attenuated several biochemical, molecular, physiological, and histological alterations, but complete restoration was not achieved, indicating only partial protection under the present experimental conditions. Because this study evaluated a single commercially available fragrance formulation, the findings should not be generalized to all fragrance products. Further investigations using additional formulations, different exposure conditions, and complementary mechanistic biomarkers are warranted to better characterize the health effects associated with repeated fragrance inhalation.

## Data Availability

The original contributions presented in the study are included in the article/supplementary material, further inquiries can be directed to the corresponding author.
