# Supplementary material for: Biological effects of a tested commercial fragrance formulation in rats: oxidative and molecular alterations and the protective role of vitamin E
Source: Front Pharmacol. 2026 Aug 26;17:1882724. doi: 10.3389/fphar.2026.1882724 (PMC13554957; doi:10.3389/fphar.2026.1882724)

My GC-MS Report

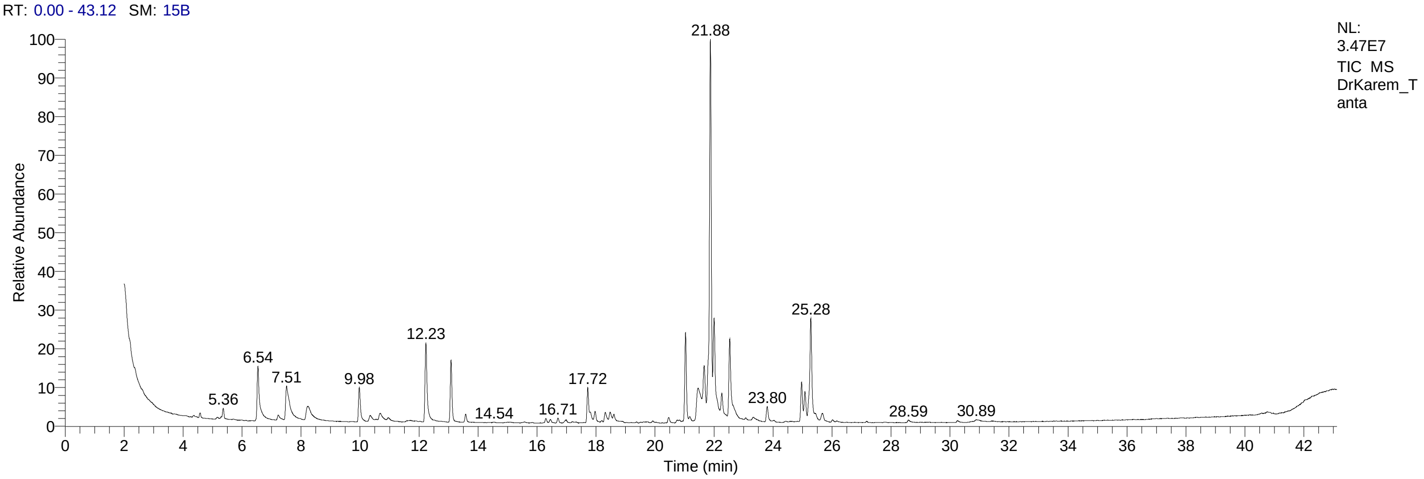

RT	Area %	Peak Area	Peak Height
4.58	0.32	1492754.26	607335.30
5.36	0.63	2993656.89	1179457.89
6.53	4.09	19306398.98	5235858.32
7.23	0.49	2302829.68	467015.91
7.50	3.17	14952700.41	2798365.68
8.23	1.66	7845692.32	991542.66
9.97	2.59	12235633.90	3439176.28
10.34	0.52	2451628.60	474573.37
10.68	0.67	3171699.39	579155.52
12.23	6.11	28851712.90	7457399.26
13.08	4.54	21433197.33	6321168.48
13.58	0.62	2949833.60	836126.06
16.30	0.37	1770063.80	500174.16
16.71	0.37	1738929.16	511791.23
17.72	2.47	11648469.97	3511470.97
17.80	0.62	2921598.19	802918.26
17.97	0.65	3063304.14	900350.47
18.31	0.82	3860461.75	878831.53
18.47	0.71	3369092.85	809025.01
18.60	0.49	2291536.71	618670.40
20.46	0.44	2065328.28	565151.60
21.04	6.42	30325507.22	9109429.55
21.45	2.94	13866712.35	2063197.09
21.67	2.81	13280256.32	3562145.08
21.88	26.87	126910547.62	36139162.37
22.00	4.92	23242300.48	7507052.41
22.27	1.33	6282806.82	1950998.49
22.53	7.43	35077562.71	7807137.47
23.80	1.19	5606043.14	1483408.08
24.97	2.92	13794716.47	3845151.19
25.08	2.07	9781104.82	2647718.04
25.28	8.08	38172660.63	9456896.73
25.67	0.70	3292859.55	651247.60

My GC-MS Report

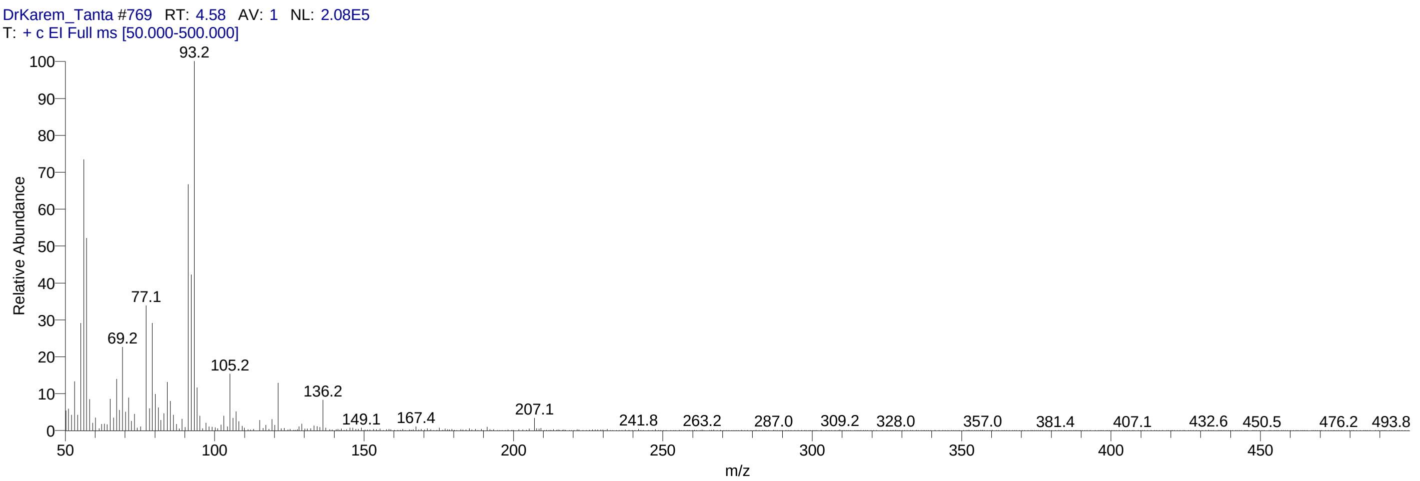

RT	Compound Name	Area %	MF	Molecular Formula	Molecular Weight	Cas #	Library
4.58	6,6-DIMETHYL-2-METHYLENE-BICYCLO[3.1.1]HEPTANE	0.32	799	C10H16	136	NA	WileyRegistry8e
4.58	BICYCLO[3.1.0]HEXANE, 4-METHYLENE-1-(1-METHYLETHYL)-	0.32	791	C10H16	136	3387-41-5	WileyRegistry8e
4.58	BICYCLO[3.1.1]HEPTANE, 6,6-DIMETHYL-2-METHYLENE-	0.32	792	C10H16	136	127-91-3	WileyRegistry8e
4.58	3,7,7-TRIMETHYLBICYCLO[4.1.0]HEPT-3-ENE	0.32	806	C10H16	136	NA	WileyRegistry8e
4.58	trans-á-Ocimene	0.32	808	C10H16	136	3779-61-1	replib

Compound Structure	Hit Spectrum

6,6-DIMETHYL-2-METHYLENE-BICYCLO[3.1.1]HEPTANE
Formula C10H16, MW 136, CAS# NA, Entry# 397119
6,6-DIMETHYL-2-METHYLENEBICYCLO[3.1.1]HEPTANE

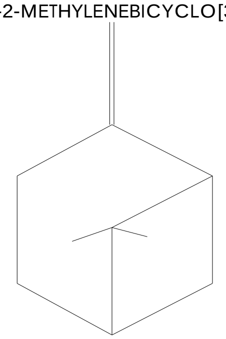

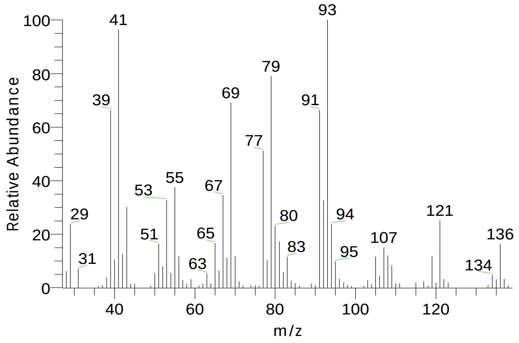

BICYCLO[3.1.0]HEXANE, 4-METHYLENE-1-(1-METHYLETHYL)-
Formula C10H16, MW 136, CAS# 3387-41-5, Entry# 23469
(+)-SABINENE

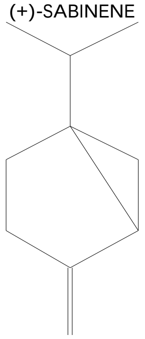

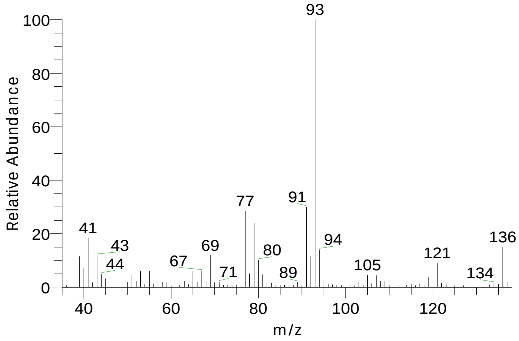

My GC-MS Report

Compound Structure | Hit Spectrum

BICYCLO[3.1.1]HEPTANE, 6,6-DIMETHYL-2-METHYLENE-
Formula C10H16, MW 136, CAS# 127-91-3, Entry# 23510
(+)-BETA-PINEN

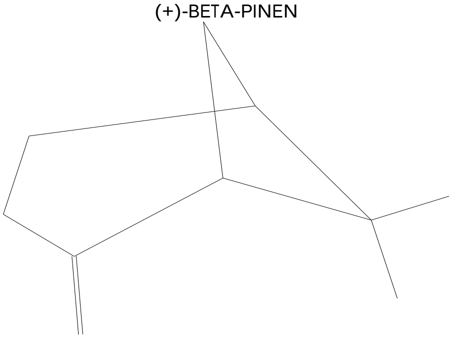

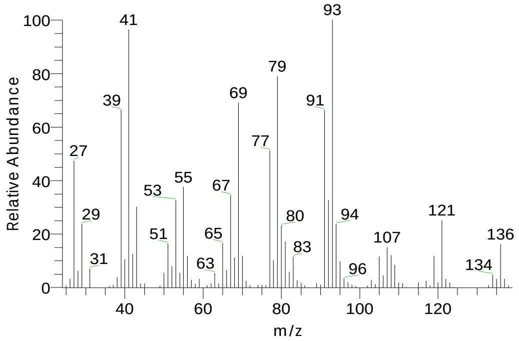

3,7,7-TRIMETHYLBICYCLO[4.1.0]HEPT-3-ENE
Formula C10H16, MW 136, CAS# NA, Entry# 359243
DELTA-3-CAREN

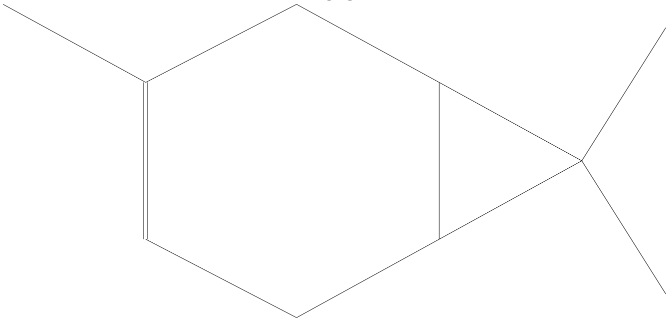

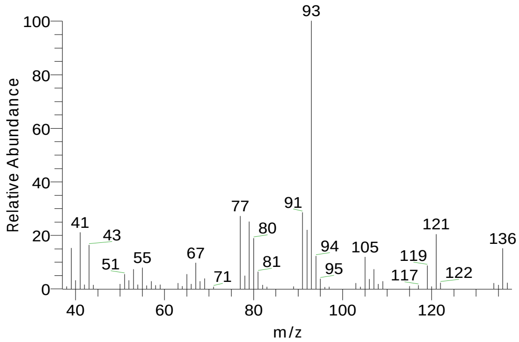

trans-á-Ocimene
Formula C10H16, MW 136, CAS# 3779-61-1, Entry# 14122
1,3,6-Octatriene, 3,7-dimethyl-, (E)-

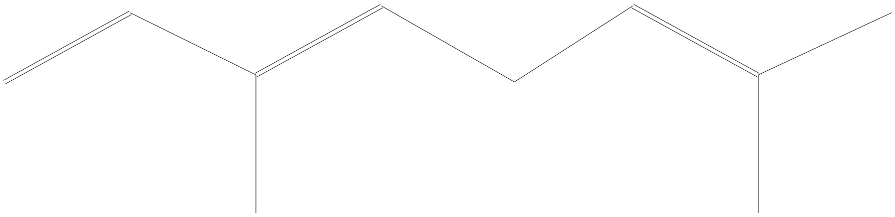

SI 730, RSI 808, replib, Entry# 14122, CAS# 3779-61-1, trans-á-Ocimene

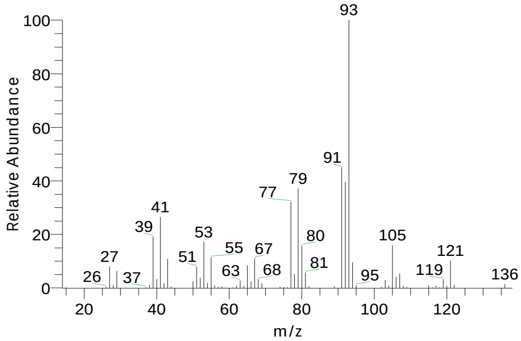

DrKarem_Tanta #1003 RT: 5.36 AV: 1 NL: 4.33E5
T: + c EI Full ms [50.000-500.000]

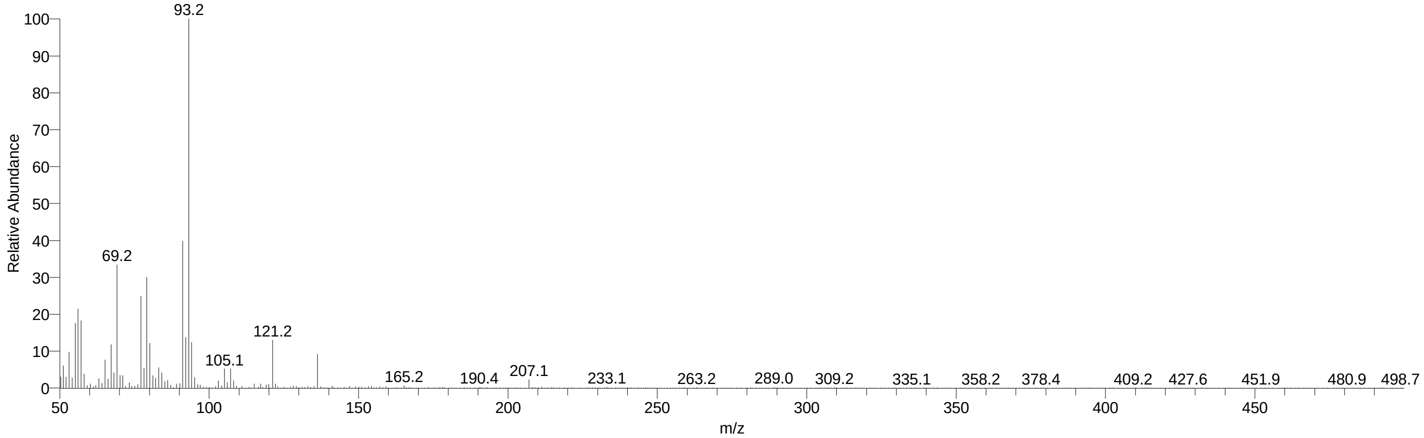

RT	Compound Name	Area %	MF	Molecular Formula	Molecular Weight	Cas #	Library
5.36	Bicyclo[3.1.1]heptane, 6,6-dimethyl-2-methylene-, (1S)-	0.63	921	C10H16	136	18172-67-3	replib
5.36	BICYCLO[3.1.1]HEPTANE, 6,6-DIMETHYL-2-METHYLENE-	0.63	870	C10H16	136	127-91-3	WileyRegistry8e

My GC-MS Report

RT	Compound Name	Area %	MF	Molecular Formula	Molecular Weight	Cas #	Library
5.36	á-Pinene	0.63	879	C10H16	136	127-91-3	mainlib
5.36	BICYCLO[3.1.1]HEPTANE, 6,6-DIMETHYL-2-METHYLENE-	0.63	883	C10H16	136	127-91-3	WileyRegistry8e
5.36	BICYCLO[3.1.1]HEPTANE, 6,6-DIMETHYL-2-METHYLENE-, (1S)-	0.63	855	C10H16	136	18172-67-3	WileyRegistry8e

Compound Structure — Hit Spectrum

Bicyclo[3.1.1]heptane, 6,6-dimethyl-2-methylene-, (1S)-
Formula C10H16, MW 136, CAS# 18172-67-3, Entry# 14004
2(10)-Pinene, (1S,5S)-(-)-

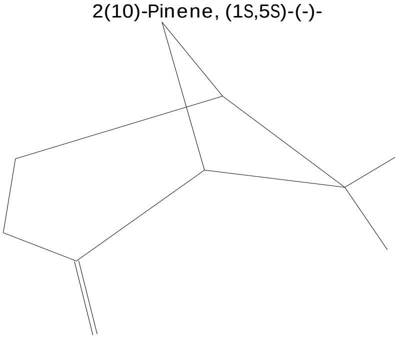

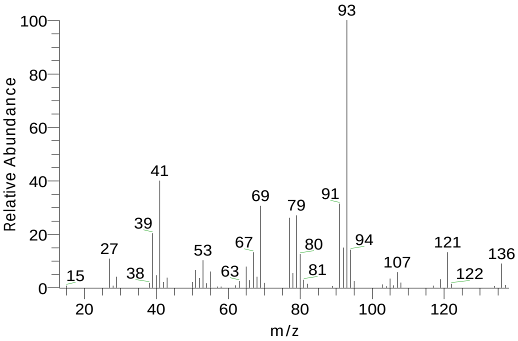

BICYCLO[3.1.1]HEPTANE, 6,6-DIMETHYL-2-METHYLENE-
Formula C10H16, MW 136, CAS# 127-91-3, Entry# 23513
(+)-BETA-PINEN

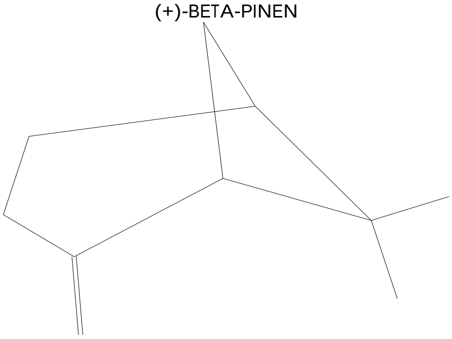

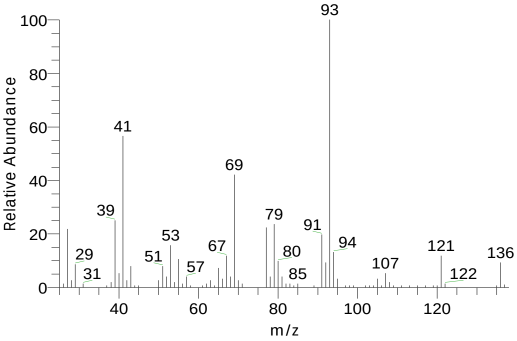

á-Pinene
Formula C10H16, MW 136, CAS# 127-91-3, Entry# 65779
Bicyclo[3.1.1]heptane, 6,6-dimethyl-2-methylene-

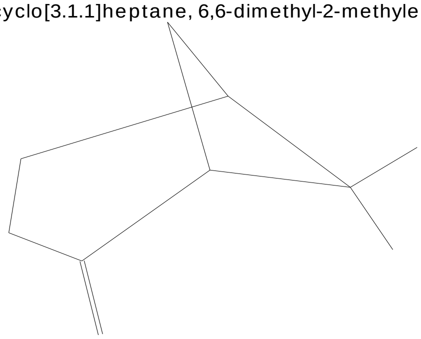

SI 830, RSI 879, mainlib, Entry# 65779, CAS# 127-91-3, á-Pinene

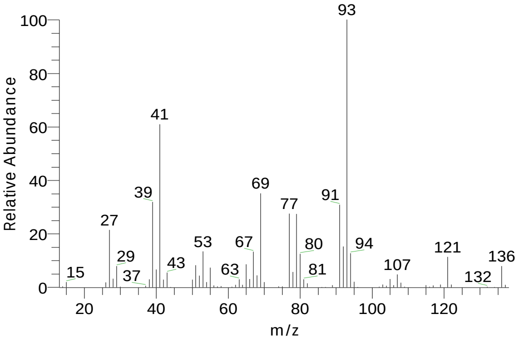

BICYCLO[3.1.1]HEPTANE, 6,6-DIMETHYL-2-METHYLENE-
Formula C10H16, MW 136, CAS# 127-91-3, Entry# 23515
(+)-BETA-PINEN

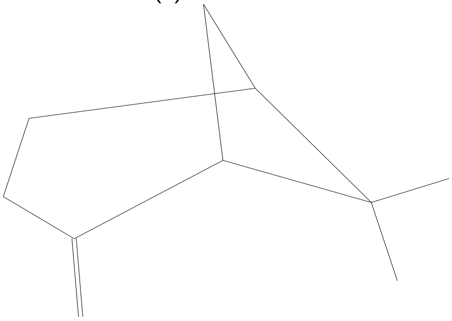

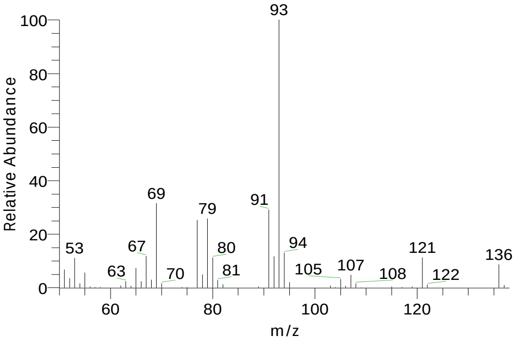

My GC-MS Report

Compound Structure | Hit Spectrum

BICYCLO[3.1.1]HEPTANE, 6,6-DIMETHYL-2-METHYLENE-, (1S)-
Formula C10H16, MW 136, CAS# 18172-67-3, Entry# 23502
6,6-DIMETHYL-2-METHYLENEBICYCLO[3.1.1]HEPTANE #

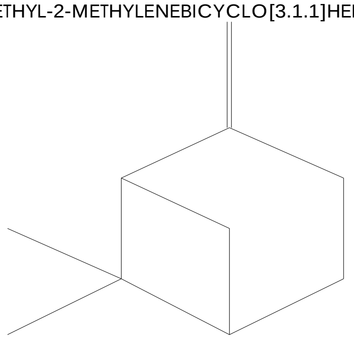

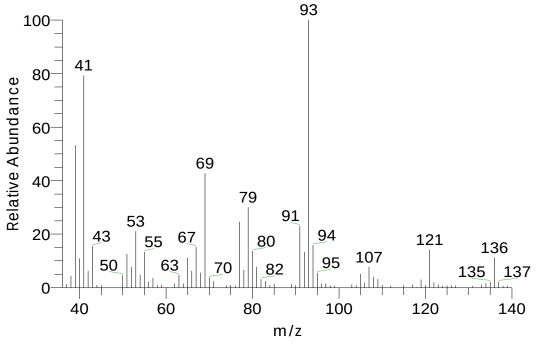

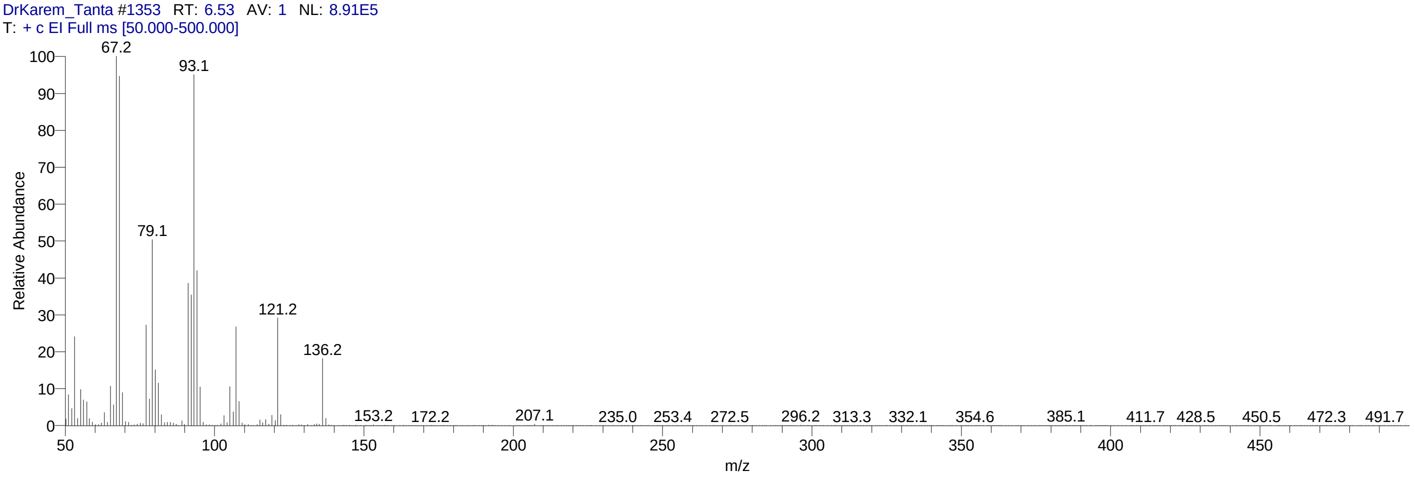

RT	Compound Name	Area %	MF	Molecular Formula	Molecular Weight	Cas #	Library
6.53	D-Limonene	4.09	912	C10H16	136	5989-27-5	replib
6.53	D-Limonene	4.09	907	C10H16	136	5989-27-5	mainlib
6.53	CYCLOHEXENE, 1-METHYL-4-(1-METHYLETHENYL)-	4.09	926	C10H16	136	138-86-3	WileyRegistry8e
6.53	Cyclohexanol, 1-methyl-4-(1-methylethenyl)-, acetate	4.09	917	C12H20O2	196	10198-23-9	replib
6.53	CYCLOHEXANOL, 1-METHYL-4-(1-METHYLETHENYL)-, ACETATE	4.09	917	C12H20O2	196	10198-23-9	WileyRegistry8e

Compound Structure | Hit Spectrum

D-Limonene
Formula C10H16, MW 136, CAS# 5989-27-5, Entry# 8354
Cyclohexene, 1-methyl-4-(1-methylethenyl)-, (R)-

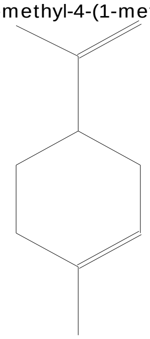

SI 905, RSI 912, replib, Entry# 8354, CAS# 5989-27-5, D-Limonene

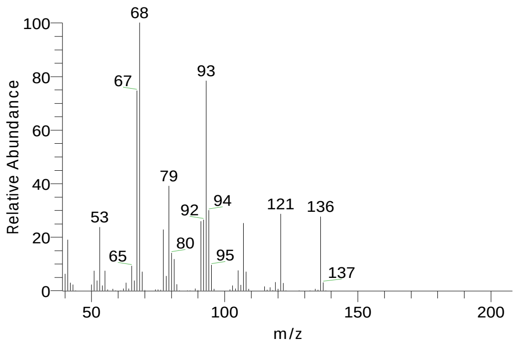

My GC-MS Report

Compound Structure | Hit Spectrum

D-Limonene
Formula C10H16, MW 136, CAS# 5989-27-5, Entry# 33688
Cyclohexene, 1-methyl-4-(1-methylethenyl)-, (R)-

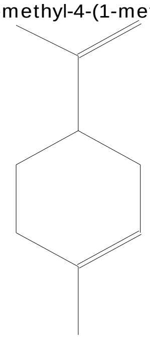

SI 904, RSI 907, mainlib, Entry# 33688, CAS# 5989-27-5, D-Limonene

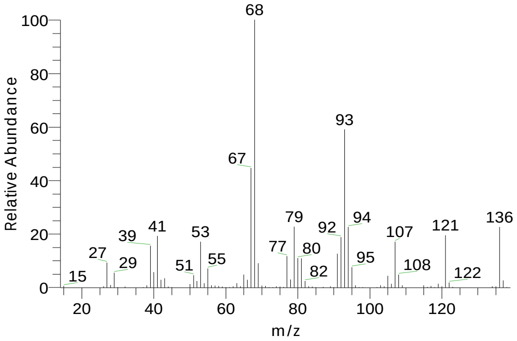

CYCLOHEXENE, 1-METHYL-4-(1-METHYLETHENYL)-
Formula C10H16, MW 136, CAS# 138-86-3, Entry# 23327
1-METHYL-4-(1-METHYLETHENYL)CYCLOHEXENE

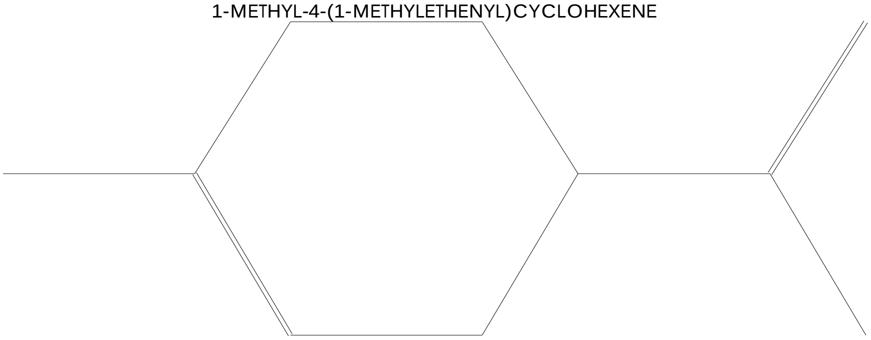

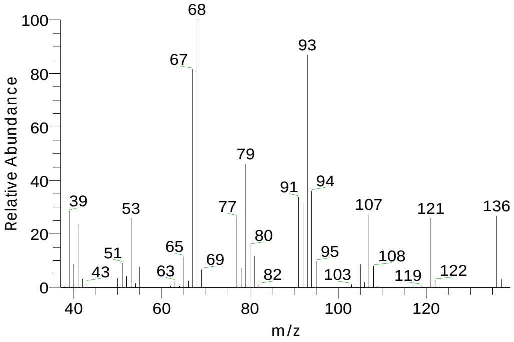

Cyclohexanol, 1-methyl-4-(1-methylethenyl)-, acetate
Formula C12H20O2, MW 196, CAS# 10198-23-9, Entry# 8355
p-Menth-8-en-1-ol, acetate

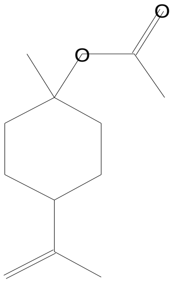

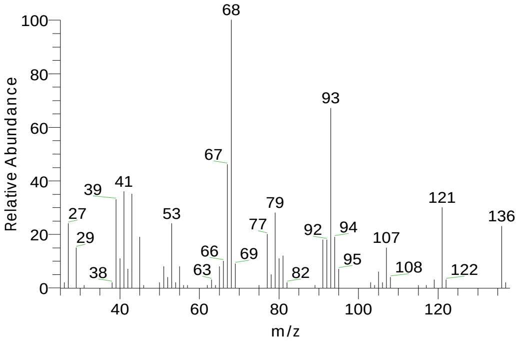

CYCLOHEXANOL, 1-METHYL-4-(1-METHYLETHENYL)-, ACETATE
Formula C12H20O2, MW 196, CAS# 10198-23-9, Entry# 80012
4-ISOPROPENYL-1-METHYLCYCLOHEXYL ACETATE #

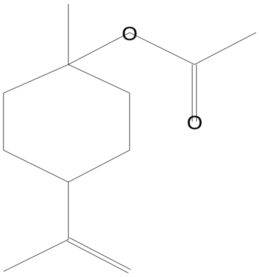

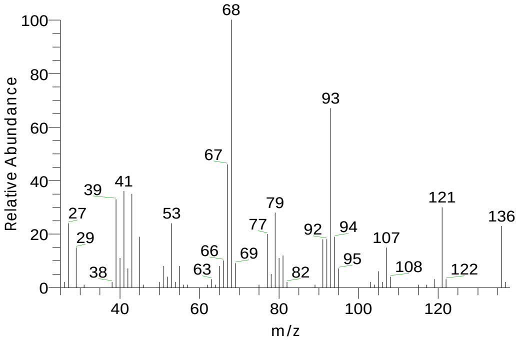

My GC-MS Report

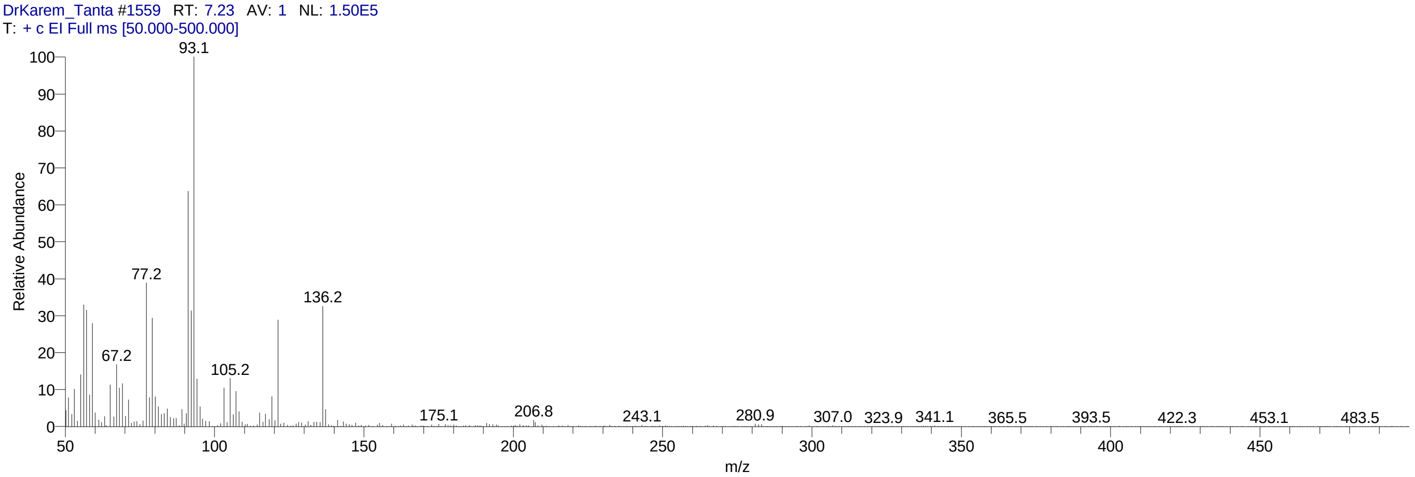

RT	Compound Name	Area %	MF	Molecular Formula	Molecular Weight	Cas #	Library
7.23	BICYCLO[3.1.0]HEXANE, 4-METHYLENE-1-(1-METHYLETHYL)-	0.49	816	C10H16	136	3387-41-5	WileyRegistry8e
7.23	4-ISOPROPENYL-1-METHYL-1-CYCLOHEXENE	0.49	833	C10H16	136	NA	WileyRegistry8e
7.23	BICYCLO[4.1.0]HEPT-3-ENE, 3,7,7-TRIMETHYL-	0.49	802	C10H16	136	13466-78-9	WileyRegistry8e
7.23	3,7,7-TRIMETHYLBICYCLO[4.1.0]HEPT-3-ENE	0.49	828	C10H16	136	NA	WileyRegistry8e
7.23	3-Carene	0.49	804	C10H16	136	13466-78-9	replib

Compound Structure	Hit Spectrum

BICYCLO[3.1.0]HEXANE, 4-METHYLENE-1-(1-METHYLETHYL)-
Formula C10H16, MW 136, CAS# 3387-41-5, Entry# 23469
(+)-SABINENE

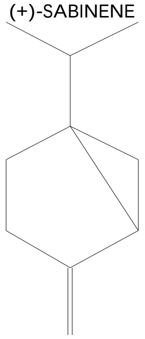

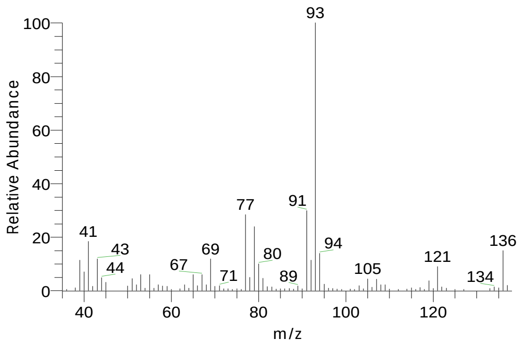

4-ISOPROPENYL-1-METHYL-1-CYCLOHEXENE
Formula C10H16, MW 136, CAS# NA, Entry# 359245
R(+)-LIMONEN

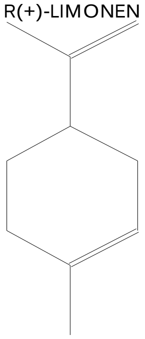

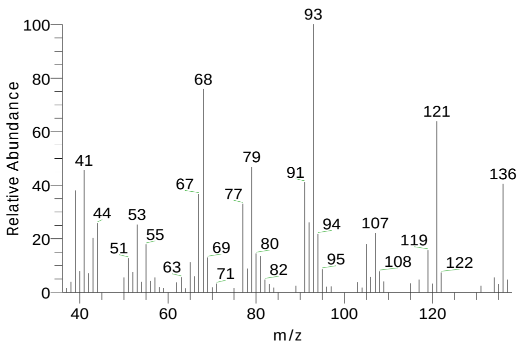

My GC-MS Report

Compound Structure | Hit Spectrum

BICYCLO[4.1.0]HEPT-3-ENE, 3,7,7-TRIMETHYL-
Formula C10H16, MW 136, CAS# 13466-78-9, Entry# 23540
3,7,7-TRIMETHYLBICYCLO[4.1.0]HEPT-3-ENE

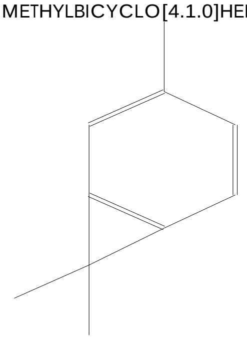

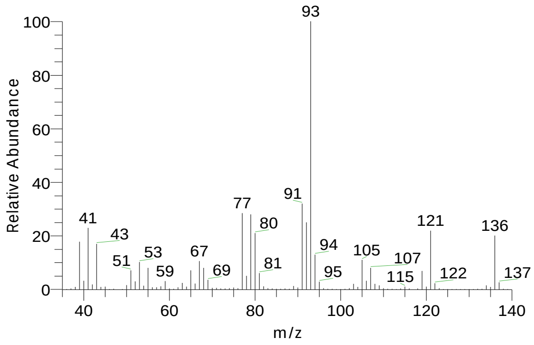

3,7,7-TRIMETHYLBICYCLO[4.1.0]HEPT-3-ENE
Formula C10H16, MW 136, CAS# NA, Entry# 359243
DELTA-3-CAREN

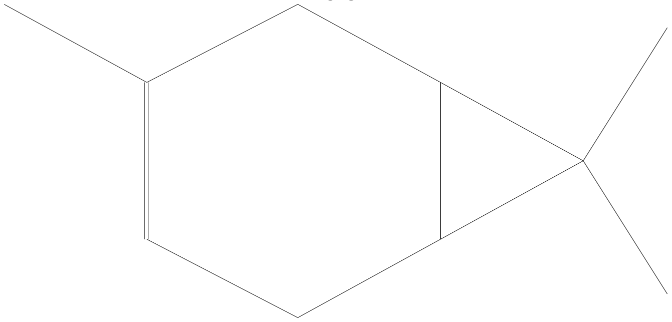

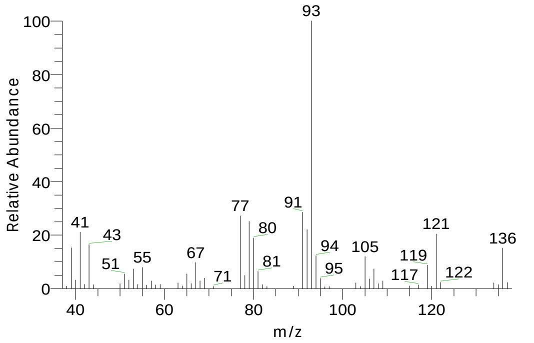

SI 760, RSI 804, replib, Entry# 14120, CAS# 13466-78-9, 3-Carene

3-Carene
Formula C10H16, MW 136, CAS# 13466-78-9, Entry# 14120
Bicyclo[4.1.0]hept-3-ene, 3,7,7-trimethyl-

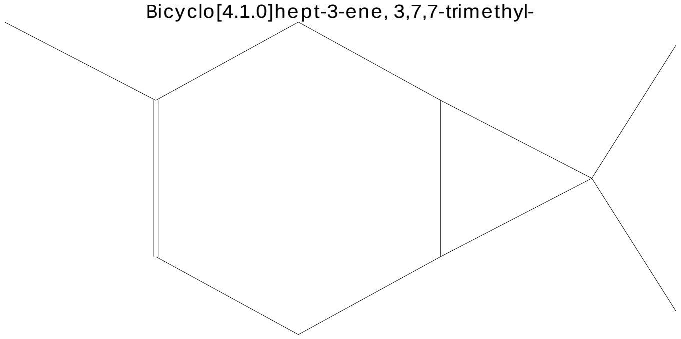

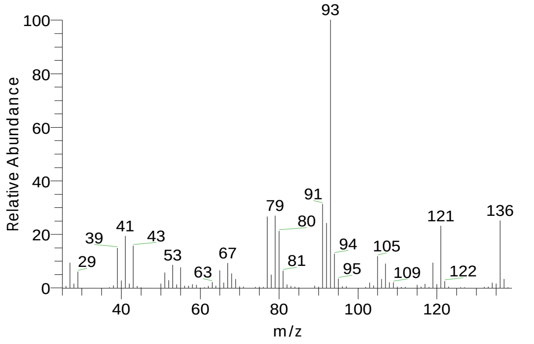

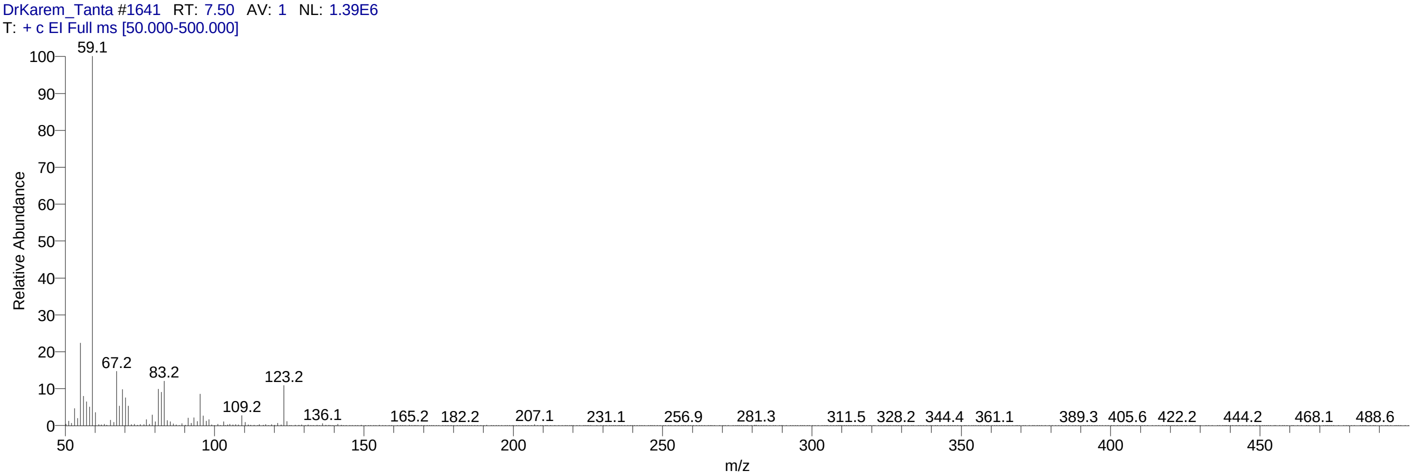

RT	Compound Name	Area %	MF	Molecular Formula	Molecular Weight	Cas #	Library
7.50	7-OCTEN-2-OL, 2-METHYL-6-METHYLENE-, DIHYDRO DERIV.	3.17	955	C10H20O	156	53219-21-9	WileyRegistry8e
7.50	7-Octen-2-ol, 2,6-dimethyl-	3.17	894	C10H20O	156	18479-58-8	replib

My GC-MS Report

RT	Compound Name	Area %	MF	Molecular Formula	Molecular Weight	Cas #	Library
7.50	7-OCTEN-2-OL, 2,6-DIMETHYL-	3.17	894	C10H20O	156	18479-58-8	WileyRegistry8e
7.50	7-Octen-2-ol, 2,6-dimethyl-	3.17	897	C10H20O	156	18479-58-8	replib
7.50	7-OCTEN-2-OL, 2-METHYL-6-METHYLENE-, DIHYDRO DERIV.	3.17	896	C10H20O	156	53219-21-9	WileyRegistry8e

Compound Structure | Hit Spectrum

7-OCTEN-2-OL, 2-METHYL-6-METHYLENE-, DIHYDRO DERIV.
Formula C10H20O, MW 156, CAS# 53219-21-9, Entry# 40804
MYRCENOL, DIHYDRO-

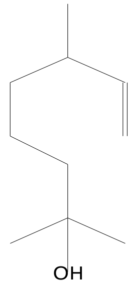

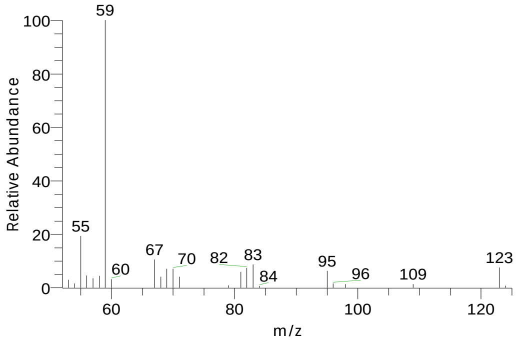

7-Octen-2-ol, 2,6-dimethyl-
Formula C10H20O, MW 156, CAS# 18479-58-8, Entry# 7268
2,6-Dimethyl-7-octen-2-ol

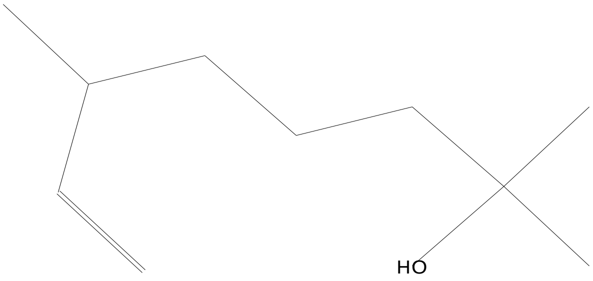

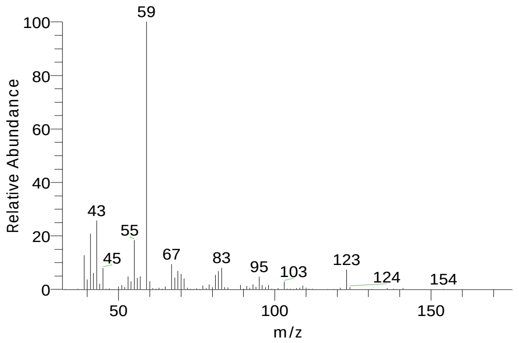

7-OCTEN-2-OL, 2,6-DIMETHYL-
Formula C10H20O, MW 156, CAS# 18479-58-8, Entry# 309473
MYRCENOL, DIHYDRO-

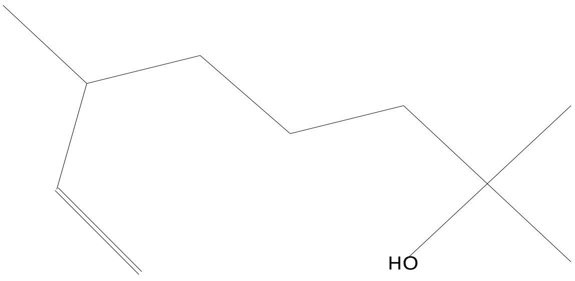

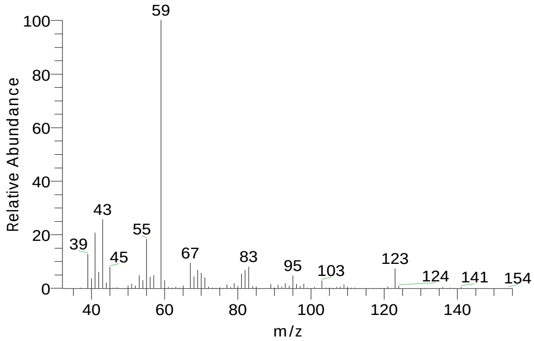

7-Octen-2-ol, 2,6-dimethyl-
Formula C10H20O, MW 156, CAS# 18479-58-8, Entry# 7327
2,6-Dimethyl-7-octen-2-ol

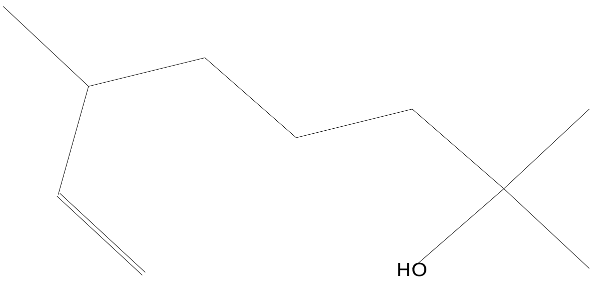

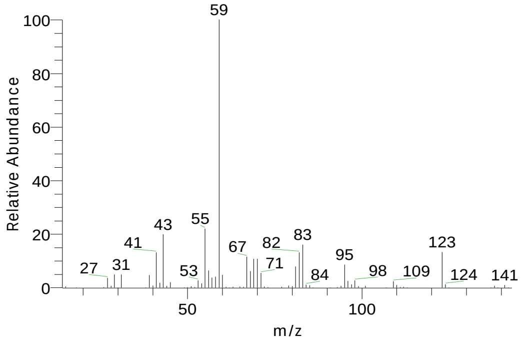

My GC-MS Report

Compound Structure | Hit Spectrum

7-OCTEN-2-OL, 2-METHYL-6-METHYLENE-, DIHYDRO DERIV.
Formula C10H20O, MW 156, CAS# 53219-21-9, Entry# 40683
MYRCENOL, DIHYDRO-

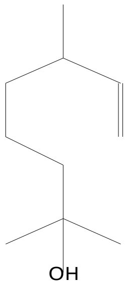

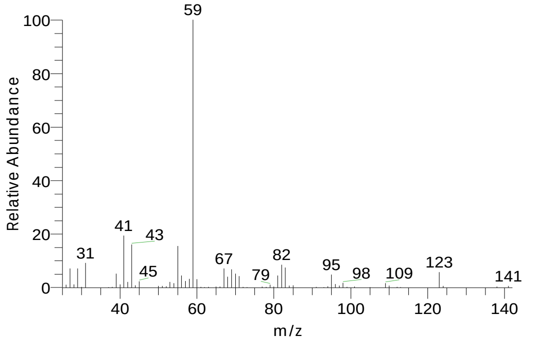

DrKarem_Tanta #1858 RT: 8.23 AV: 1 NL: 2.00E5
T: + c EI Full ms [50.000-500.000]

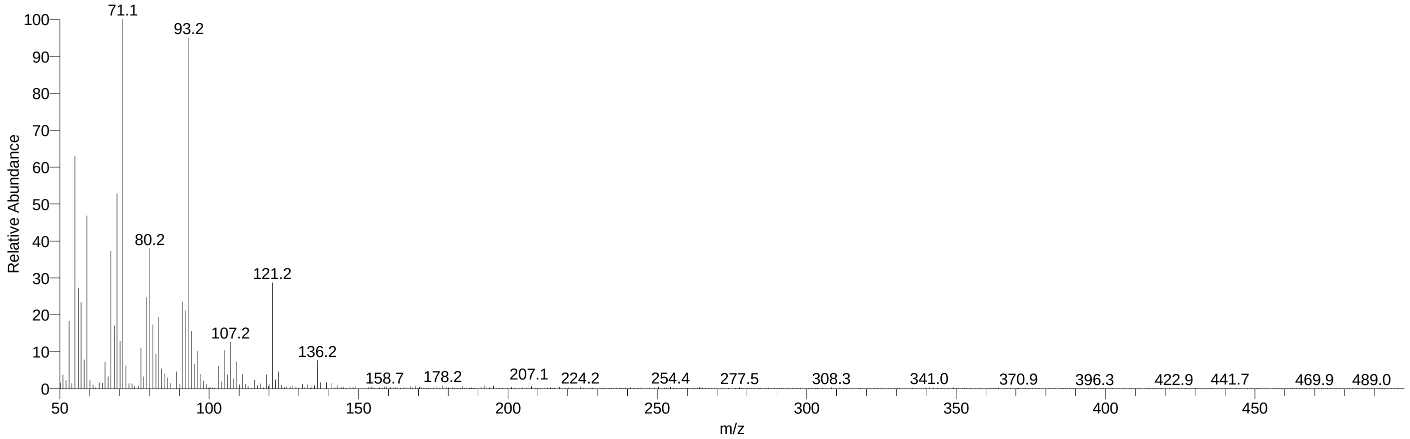

RT	Compound Name	Area %	MF	Molecular Formula	Molecular Weight	Cas #	Library
8.23	1,6-OCTADIEN-3-OL, 3,7-DIMETHYL-	1.66	911	C10H18O	154	78-70-6	WileyRegistry8e
8.23	1,6-OCTADIEN-3-OL, 3,7-DIMETHYL-	1.66	909	C10H18O	154	78-70-6	WileyRegistry8e
8.23	Linalool	1.66	877	C10H18O	154	78-70-6	replib
8.23	1,6-OCTADIEN-3-OL, 3,7-DIMETHYL-	1.66	887	C10H18O	154	78-70-6	WileyRegistry8e
8.23	1,6-OCTADIEN-3-OL, 3,7-DIMETHYL-	1.66	852	C10H18O	154	78-70-6	WileyRegistry8e

Compound Structure | Hit Spectrum

1,6-OCTADIEN-3-OL, 3,7-DIMETHYL-
Formula C10H18O, MW 154, CAS# 78-70-6, Entry# 38259
3,7-DIMETHYLOCTA-1,6-DIEN-3-OL

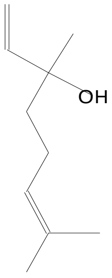

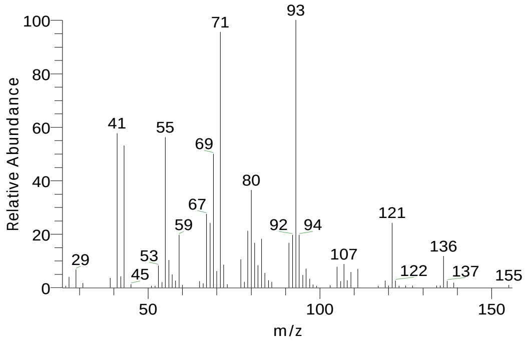

My GC-MS Report

Compound Structure | Hit Spectrum

1,6-OCTADIEN-3-OL, 3,7-DIMETHYL-
Formula C10H18O, MW 154, CAS# 78-70-6, Entry# 38260
3,7-DIMETHYLOCTA-1,6-DIEN-3-OL

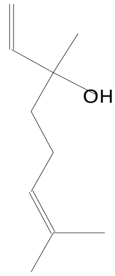

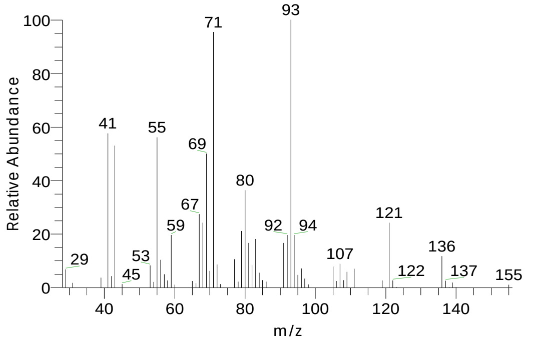

Linalool
Formula C10H18O, MW 154, CAS# 78-70-6, Entry# 9240
1,6-Octadien-3-ol, 3,7-dimethyl-

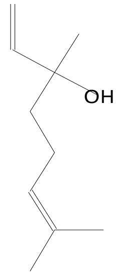

SI 846, RSI 877, replib, Entry# 9240, CAS# 78-70-6, Linalool

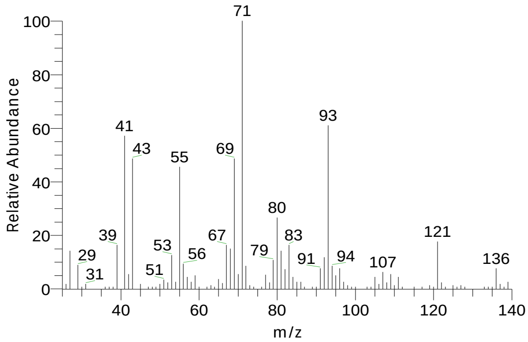

1,6-OCTADIEN-3-OL, 3,7-DIMETHYL-
Formula C10H18O, MW 154, CAS# 78-70-6, Entry# 309549
3,7-DIMETHYLOCTA-1,6-DIEN-3-OL

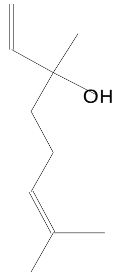

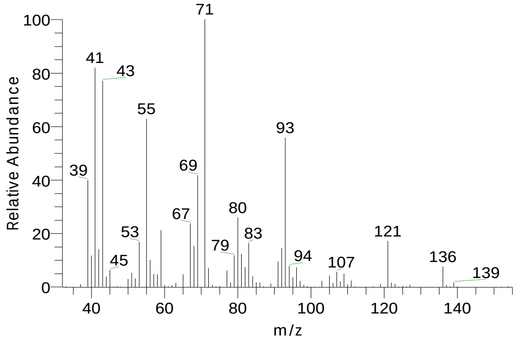

1,6-OCTADIEN-3-OL, 3,7-DIMETHYL-
Formula C10H18O, MW 154, CAS# 78-70-6, Entry# 38251
3,7-DIMETHYLOCTA-1,6-DIEN-3-OL

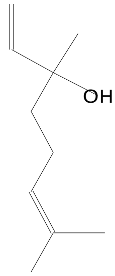

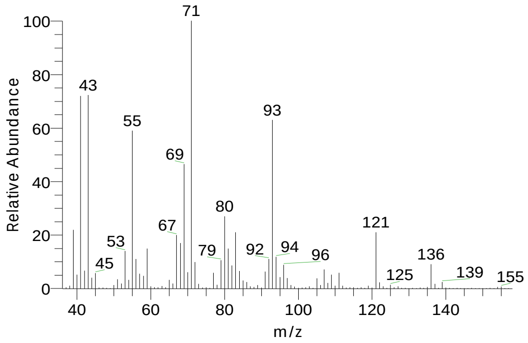

My GC-MS Report

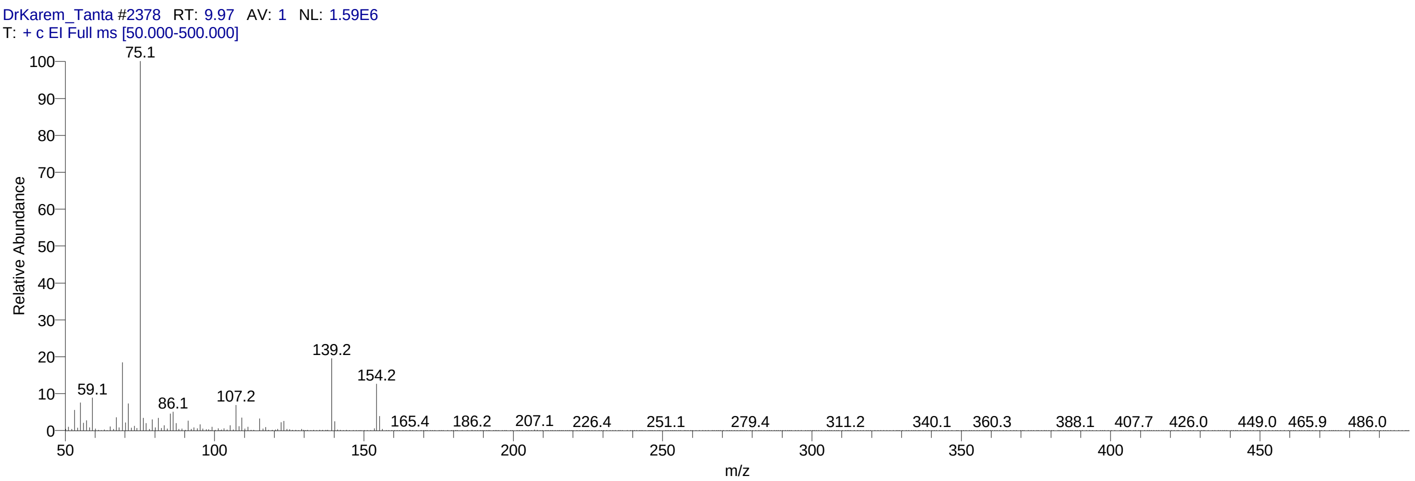

RT	Compound Name	Area %	MF	Molecular Formula	Molecular Weight	Cas #	Library
9.97	2-Hexene, 6,6-dimethoxy-2,5,5-trimethyl-	2.59	898	C11H22O2	186	67674-46-8	mainlib
9.97	3-CYCLOPENTENE-1-METHANOL, 2,2,3,4-TETRAMETHYL-, (S)-	2.59	743	C10H18O	154	104086-71-7	WileyRegistry8e
9.97	1-CYCLOHEXENE-1-ETHANOL, 3,3-DIMETHYL-	2.59	711	C10H18O	154	95495-80-0	WileyRegistry8e
9.97	Cyclohexene, 4-(dimethoxymethyl)-1-methyl-	2.59	658	C10H18O2	170	7560-66-9	mainlib
9.97	CYCLOHEXENE, 4-(DIMETHOXYMETHYL)-1-METHYL-	2.59	658	C10H18O2	170	7560-66-9	WileyRegistry8e

Compound Structure | Hit Spectrum

2-Hexene, 6,6-dimethoxy-2,5,5-trimethyl-
Formula C11H22O2, MW 186, CAS# 67674-46-8, Entry# 45234
1,1-Dimethoxy-2,2,5-trimethylhex-4-ene

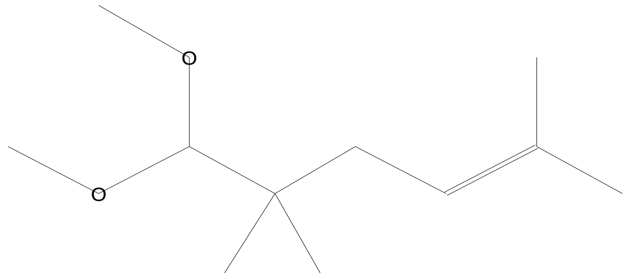

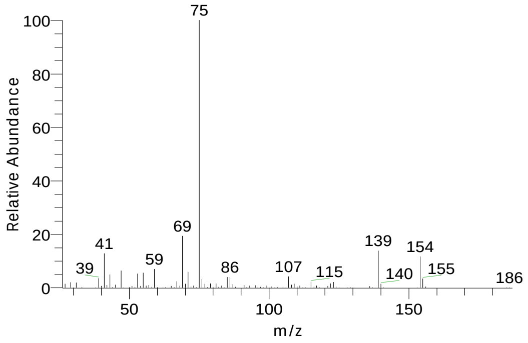

3-CYCLOPENTENE-1-METHANOL, 2,2,3,4-TETRAMETHYL-, (S)-
Formula C10H18O, MW 154, CAS# 104086-71-7, Entry# 38836
(1R,3R)-3-(HYDROXYMETHYL)-1,4,4,5-TETRAMETHYLCYCLOPENTENE

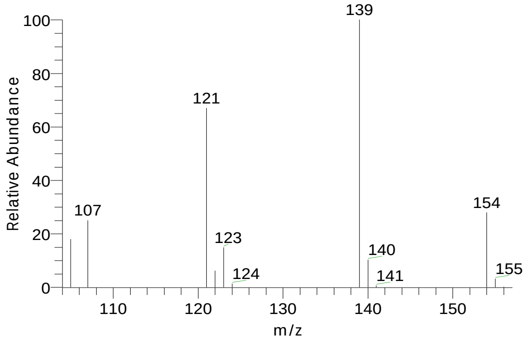

My GC-MS Report

Compound Structure | Hit Spectrum

1-CYCLOHEXENE-1-ETHANOL, 3,3-DIMETHYL-
Formula C10H18O, MW 154, CAS# 95495-80-0, Entry# 38852
3-OCHTODEN-1-OL

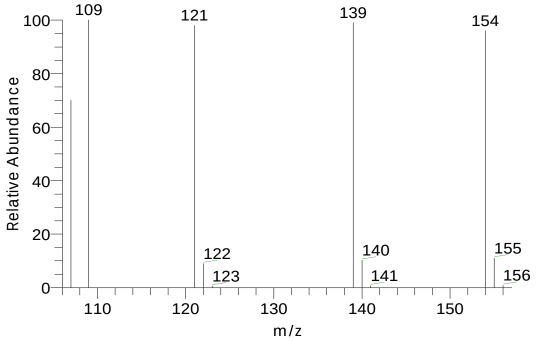

Cyclohexene, 4-(dimethoxymethyl)-1-methyl-
Formula C10H18O2, MW 170, CAS# 7560-66-9, Entry# 45648
3-Cyclohexene-1-carboxaldehyde, 4-methyl-, dimethyl acetal

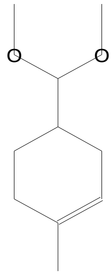

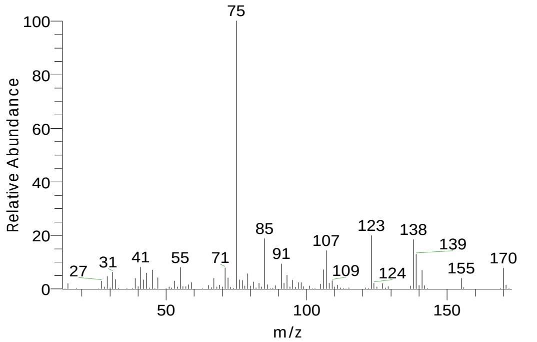

CYCLOHEXENE, 4-(DIMETHOXYMETHYL)-1-METHYL-
Formula C10H18O2, MW 170, CAS# 7560-66-9, Entry# 53551
4-(DIMETHOXYMETHYL)-1-METHYL-1-CYCLOHEXENE #

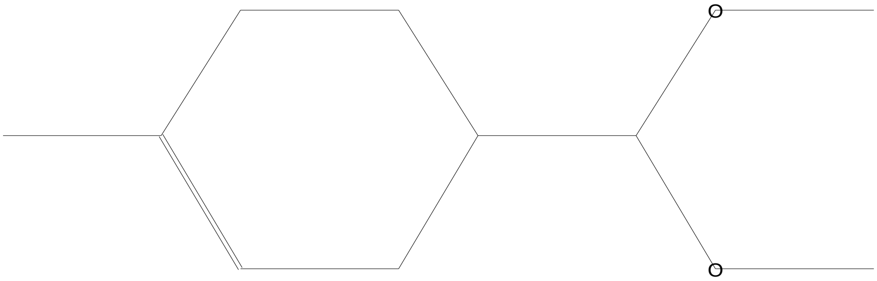

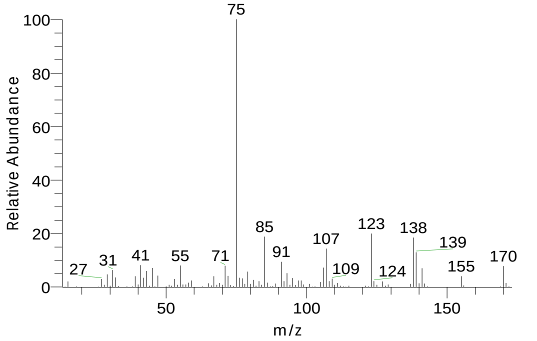

DrKarem_Tanta #2488 RT: 10.34 AV: 1 NL: 9.13E4
T: + c EI Full ms [50.000-500.000]

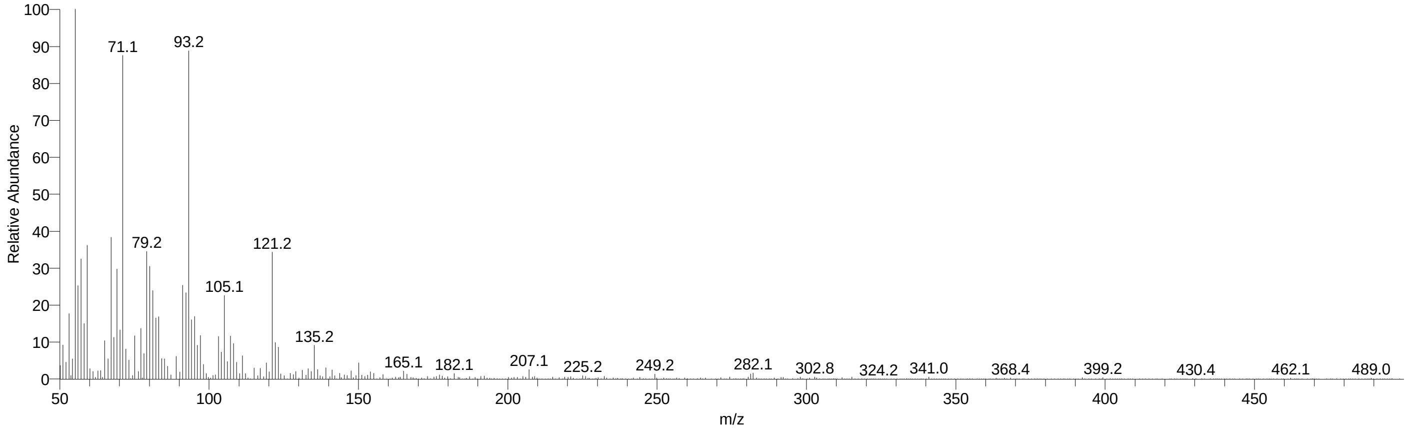

RT	Compound Name	Area %	MF	Molecular Formula	Molecular Weight	Cas #	Library
10.34	Linalool	0.52	824	C10H18O	154	78-70-6	replib
10.34	1,6-OCTADIEN-3-OL, 3,7-DIMETHYL-	0.52	776	C10H18O	154	78-70-6	WileyRegistry8e
10.34	1,6-OCTADIEN-3-OL, 3,7-DIMETHYL-	0.52	829	C10H18O	154	78-70-6	WileyRegistry8e

My GC-MS Report

RT	Compound Name	Area %	MF	Molecular Formula	Molecular Weight	Cas #	Library
10.34	1,6-OCTADIEN-3-OL, 3,7-DIMETHYL-	0.52	812	C10H18O	154	78-70-6	WileyRegistry8e
10.34	3,7-DIMETHYL-1,6-OCTADIEN-3-OL	0.52	787	C10H18O	154	NA	WileyRegistry8e

Compound Structure | Hit Spectrum

Linalool
Formula C10H18O, MW 154, CAS# 78-70-6, Entry# 9240
1,6-Octadien-3-ol, 3,7-dimethyl-

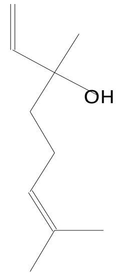

SI 749, RSI 824, replib, Entry# 9240, CAS# 78-70-6, Linalool

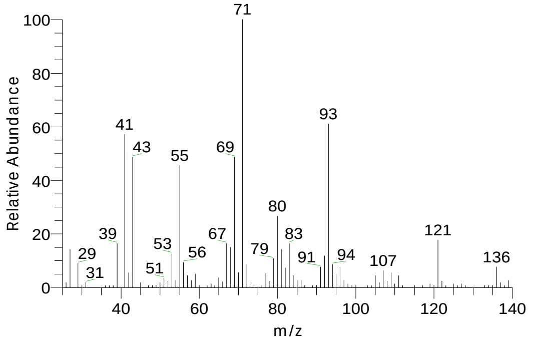

1,6-OCTADIEN-3-OL, 3,7-DIMETHYL-
Formula C10H18O, MW 154, CAS# 78-70-6, Entry# 38251
3,7-DIMETHYLOCTA-1,6-DIEN-3-OL

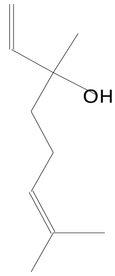

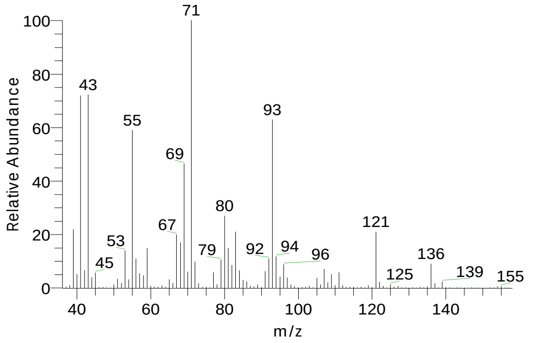

1,6-OCTADIEN-3-OL, 3,7-DIMETHYL-
Formula C10H18O, MW 154, CAS# 78-70-6, Entry# 309549
3,7-DIMETHYLOCTA-1,6-DIEN-3-OL

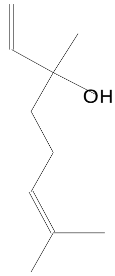

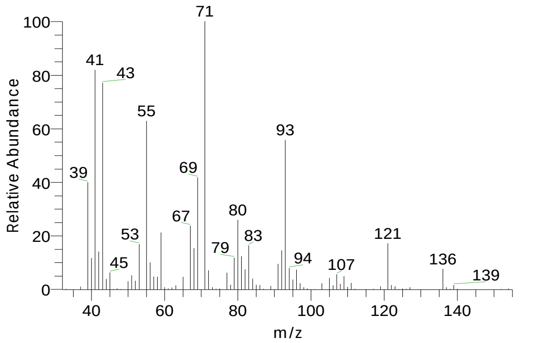

1,6-OCTADIEN-3-OL, 3,7-DIMETHYL-
Formula C10H18O, MW 154, CAS# 78-70-6, Entry# 38239
3,7-DIMETHYLOCTA-1,6-DIEN-3-OL

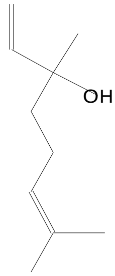

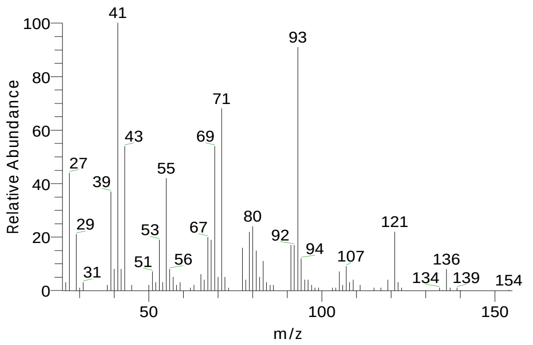

My GC-MS Report

Compound Structure | Hit Spectrum

3,7-DIMETHYL-1,6-OCTADIEN-3-OL
Formula C10H18O, MW 154, CAS# NA, Entry# 383434
BETA-LINALOOL

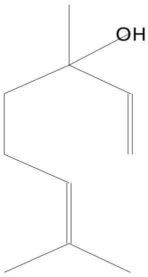

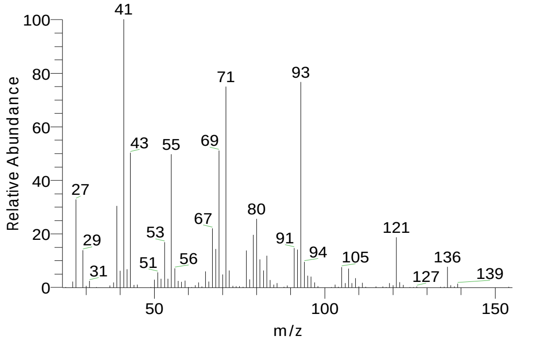

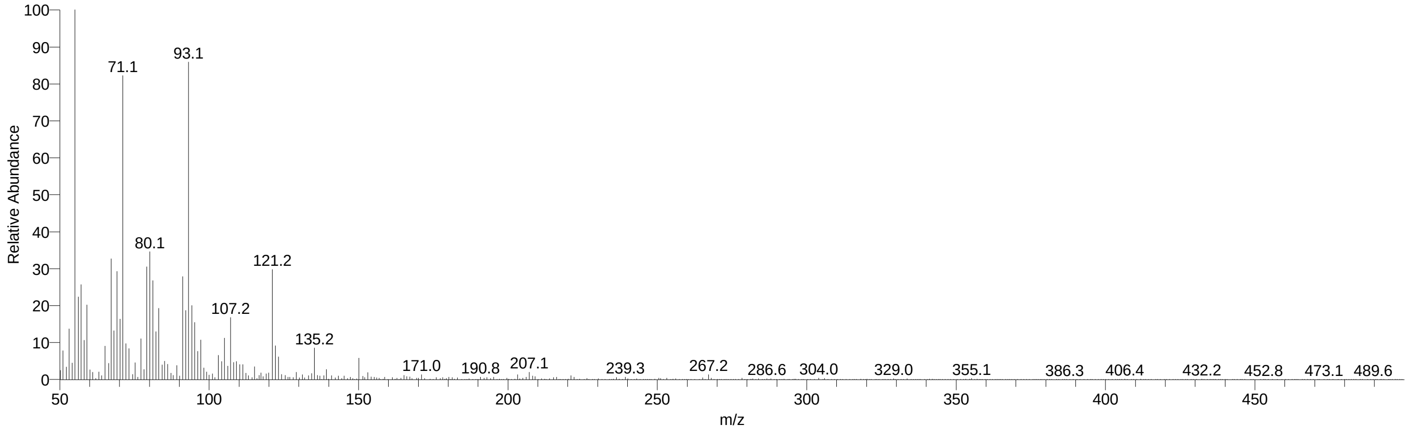

RT	Compound Name	Area %	MF	Molecular Formula	Molecular Weight	Cas #	Library
10.68	Linalool	0.67	822	C10H18O	154	78-70-6	replib
10.68	1,6-OCTADIEN-3-OL, 3,7-DIMETHYL-	0.67	829	C10H18O	154	78-70-6	WileyRegistry8e
10.68	8,11,14-EICOSATRIENOIC ACID, (Z,Z,Z)-	0.67	766	C20H34O2	306	1783-84-2	WileyRegistry8e
10.68	1,6-Octadiene, 3-ethoxy-3,7-dimethyl-	0.67	797	C12H22O	182	72845-33-1	mainlib
10.68	8,11,14-Eicosatrienoic acid, (Z,Z,Z)-	0.67	764	C20H34O2	306	1783-84-2	replib

Compound Structure | Hit Spectrum

Linalool
Formula C10H18O, MW 154, CAS# 78-70-6, Entry# 9240
1,6-Octadien-3-ol, 3,7-dimethyl-

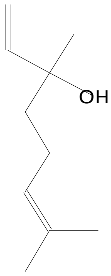

SI 764, RSI 822, replib, Entry# 9240, CAS# 78-70-6, Linalool

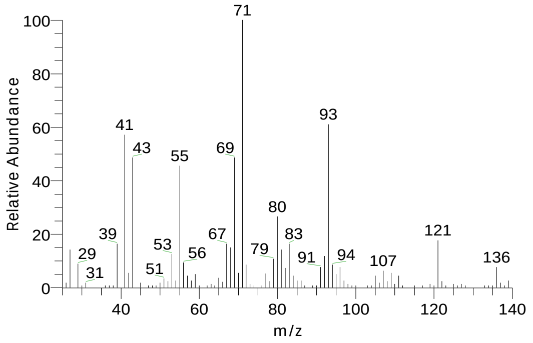

My GC-MS Report

Compound Structure | Hit Spectrum

1,6-OCTADIEN-3-OL, 3,7-DIMETHYL-
Formula C10H18O, MW 154, CAS# 78-70-6, Entry# 38239
3,7-DIMETHYLOCTA-1,6-DIEN-3-OL

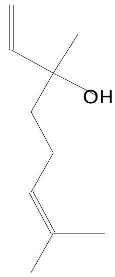

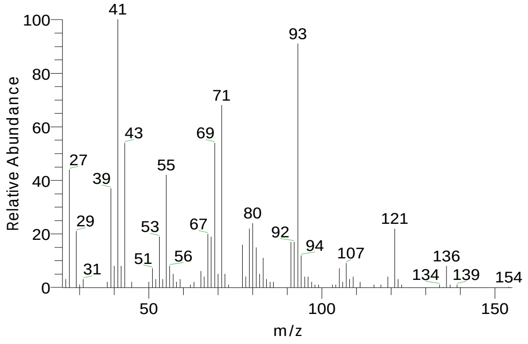

8,11,14-EICOSATRIENOIC ACID, (Z,Z,Z)-
Formula C20H34O2, MW 306, CAS# 1783-84-2, Entry# 195094
(8E,11E,14E)-8,11,14-ICOSATRIENOIC ACID #

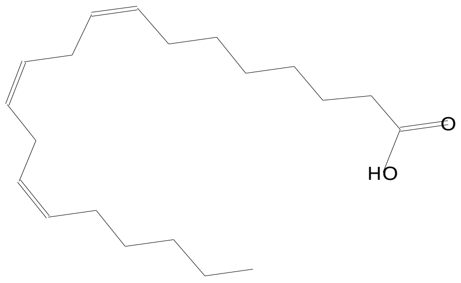

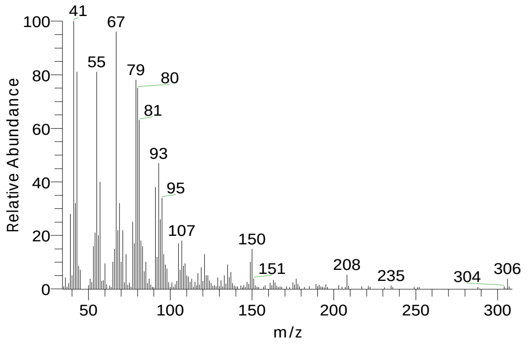

1,6-Octadiene, 3-ethoxy-3,7-dimethyl-
Formula C12H22O, MW 182, CAS# 72845-33-1, Entry# 8842
3-Ethoxy-3,7-dimethyl-1,6-octadiene

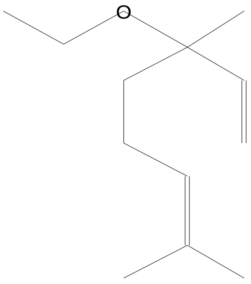

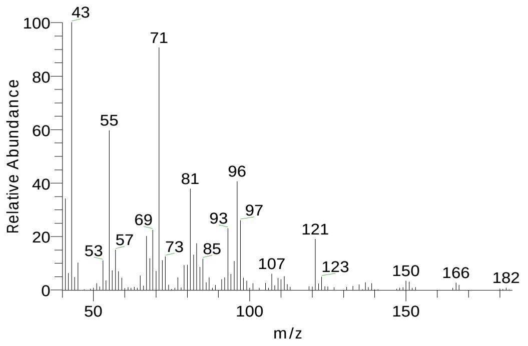

8,11,14-Eicosatrienoic acid, (Z,Z,Z)-
Formula C20H34O2, MW 306, CAS# 1783-84-2, Entry# 1122
cis-8,11,14-Eicosatrienoic Acid

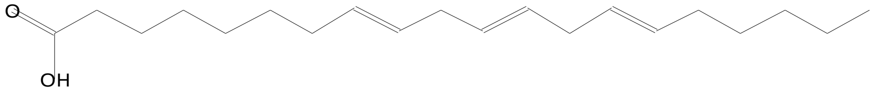

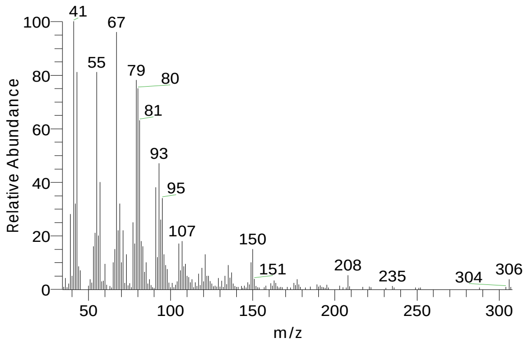

My GC-MS Report

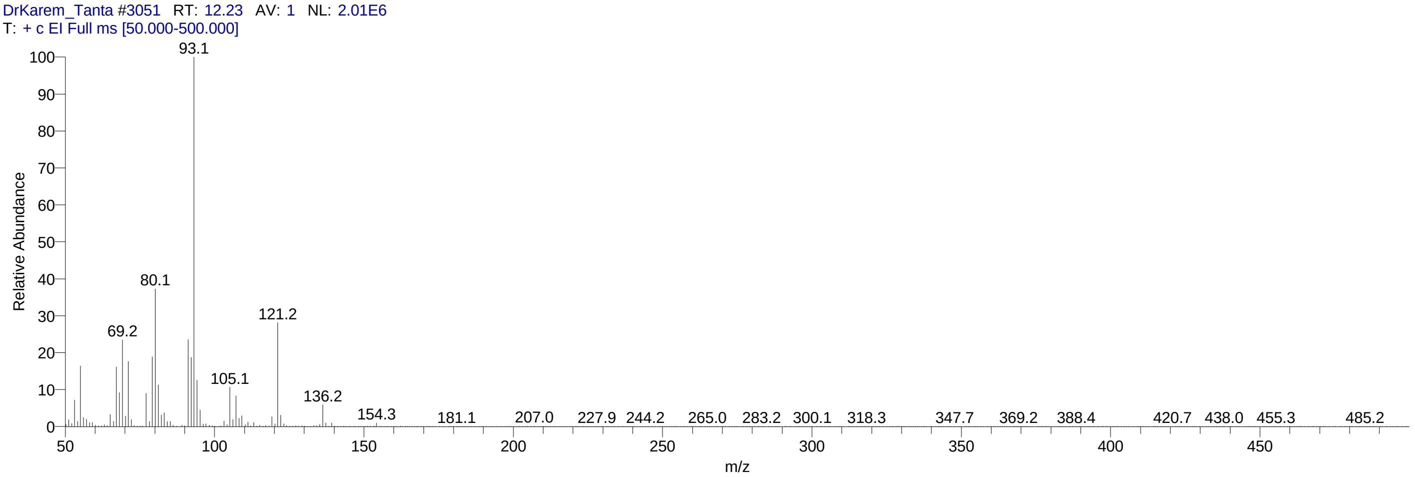

RT	Compound Name	Area %	MF	Molecular Formula	Molecular Weight	Cas #	Library
12.23	1,5-DIMETHYL-1-VINYL-4-HEXENYL 2-AMINOBENZOATE #	6.11	949	C17H23NO2	273	7149-26-0	WileyRegistry8e
12.23	1,6-OCTADIEN-3-OL, 3,7-DIMETHYL-, ACETATE	6.11	946	C12H20O2	196	115-95-7	WileyRegistry8e
12.23	Linalyl acetate	6.11	938	C12H20O2	196	115-95-7	mainlib
12.23	1,6-OCTADIEN-3-OL, 3,7-DIMETHYL-, ACETATE	6.11	940	C12H20O2	196	115-95-7	WileyRegistry8e
12.23	1,6-OCTADIEN-3-OL, 3,7-DIMETHYL-, ACETATE	6.11	928	C12H20O2	196	115-95-7	WileyRegistry8e

Compound Structure | Hit Spectrum

1,5-DIMETHYL-1-VINYL-4-HEXENYL 2-AMINOBENZOATE #
Formula C17H23NO2, MW 273, CAS# 7149-26-0, Entry# 163952
1, 6-OCTADIEN-3-OL, 3,7-DIMETHYL-, 2-AMINOBENZOATE

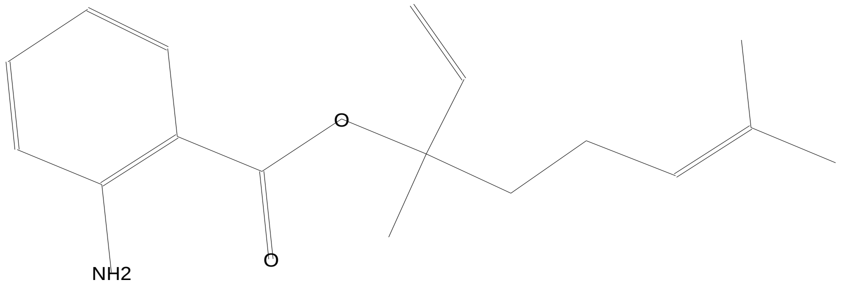

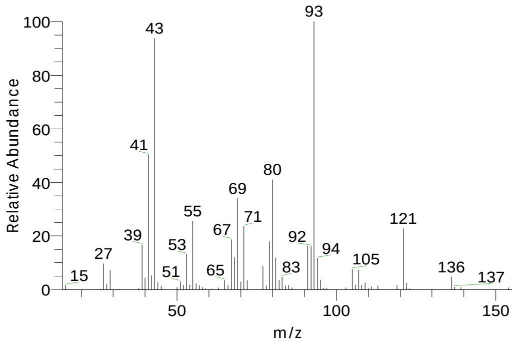

1,6-OCTADIEN-3-OL, 3,7-DIMETHYL-, ACETATE
Formula C12H20O2, MW 196, CAS# 115-95-7, Entry# 80154
(1)-1,5-DIMETHYL-1-VINYLHEX-4-ENYL ACETATE

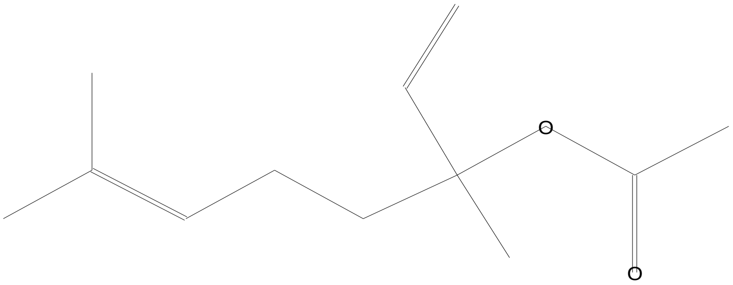

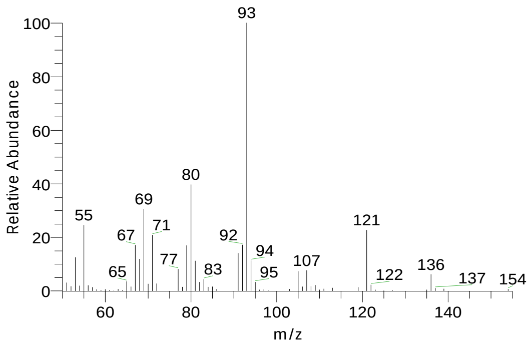

My GC-MS Report

Compound Structure	Hit Spectrum

Linalyl acetate
Formula C12H20O2, MW 196, CAS# 115-95-7, Entry# 65823
1,6-Octadien-3-ol, 3,7-dimethyl-, acetate

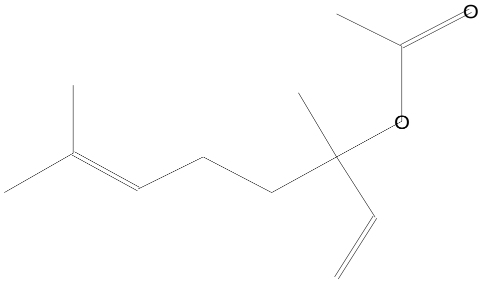

SI 936, RSI 938, mainlib, Entry# 65823, CAS# 115-95-7, Linalyl acetate

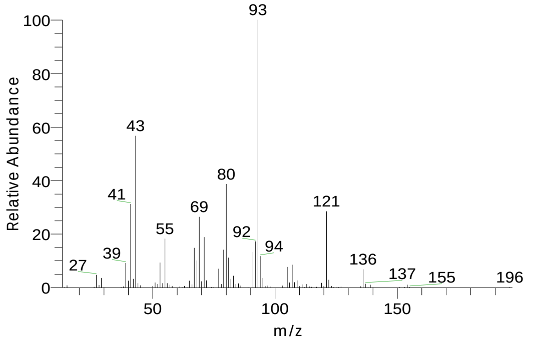

1,6-OCTADIEN-3-OL, 3,7-DIMETHYL-, ACETATE
Formula C12H20O2, MW 196, CAS# 115-95-7, Entry# 80128
(1)-1,5-DIMETHYL-1-VINYLHEX-4-ENYL ACETATE

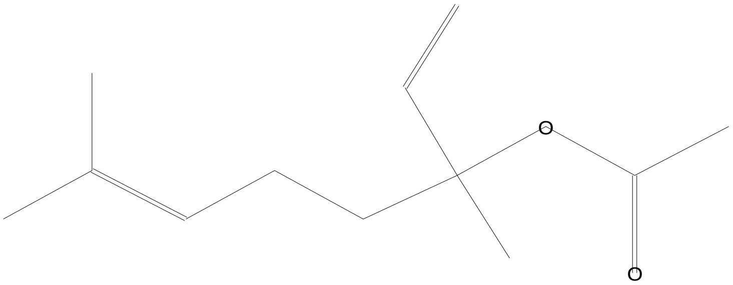

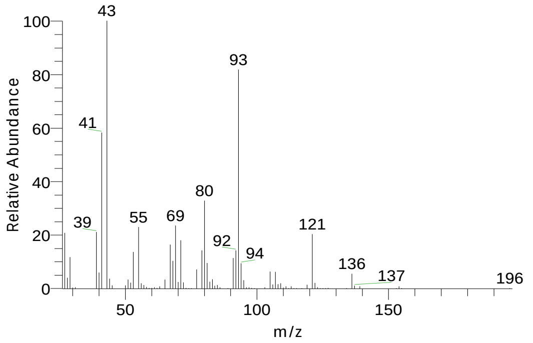

1,6-OCTADIEN-3-OL, 3,7-DIMETHYL-, ACETATE
Formula C12H20O2, MW 196, CAS# 115-95-7, Entry# 80148
(1)-1,5-DIMETHYL-1-VINYLHEX-4-ENYL ACETATE

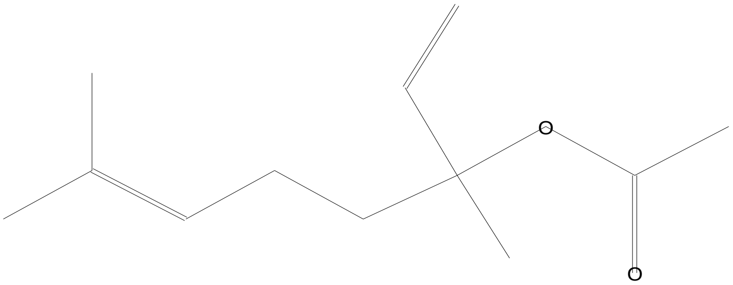

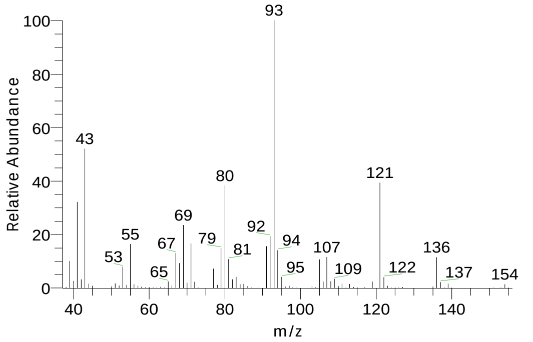

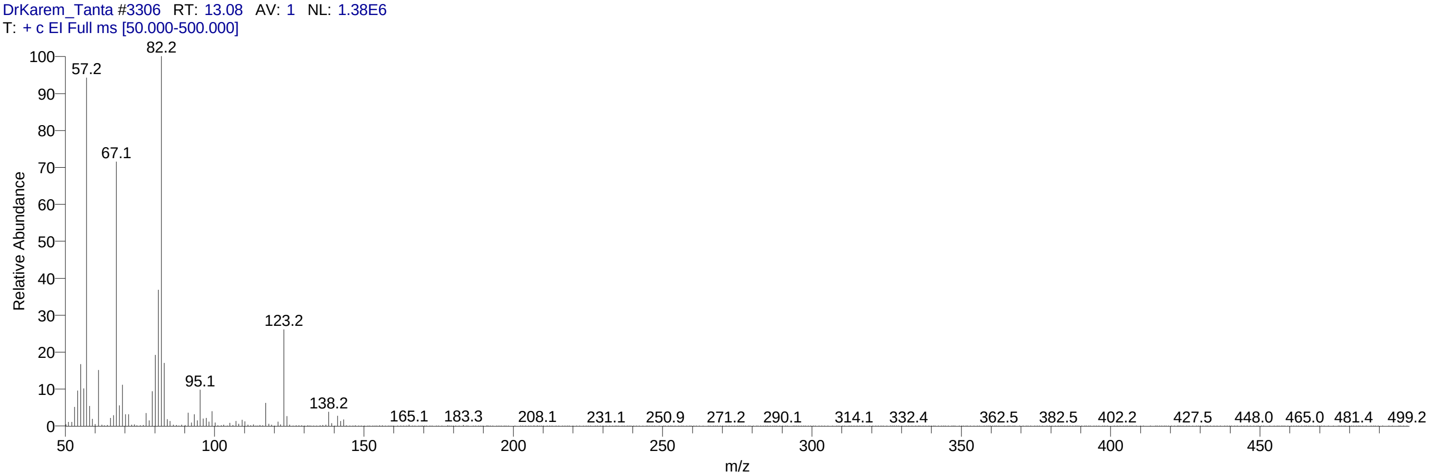

RT	Compound Name	Area %	MF	Molecular Formula	Molecular Weight	Cas #	Library
13.08	4-tert-Butylcyclohexyl acetate	4.54	855	C12H22O2	198	32210-23-4	mainlib
13.08	4-tert-Butylcyclohexyl acetate	4.54	850	C12H22O2	198	32210-23-4	replib

My GC-MS Report

RT	Compound Name	Area %	MF	Molecular Formula	Molecular Weight	Cas #	Library
13.08	Cyclohexanol, 2-(1,1-dimethylethyl)-	4.54	869	C10H20O	156	13491-79-7	mainlib
13.08	Cyclohexanol, 2-(1,1-dimethylethyl)-	4.54	862	C10H20O	156	13491-79-7	replib
13.08	CYCLOHEXANOL, 2-(1,1-DIMETHYLETHYL)-	4.54	862	C10H20O	156	13491-79-7	WileyRegistry8e

Compound Structure | Hit Spectrum

4-tert-Butylcyclohexyl acetate
Formula C12H22O2, MW 198, CAS# 32210-23-4, Entry# 24427
(4-tert-Butylcyclohexyl)acetate

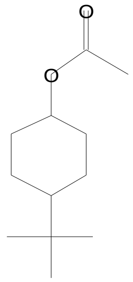

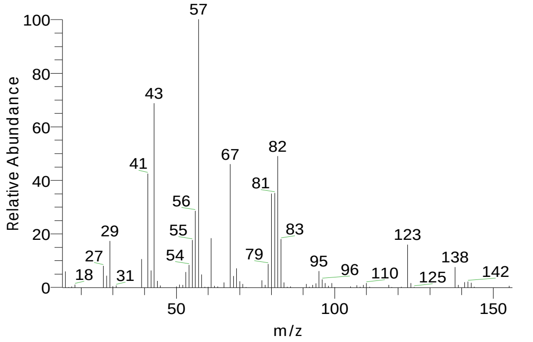

4-tert-Butylcyclohexyl acetate
Formula C12H22O2, MW 198, CAS# 32210-23-4, Entry# 6476
(4-tert-Butylcyclohexyl)acetate

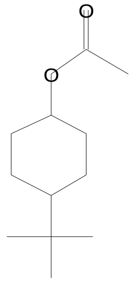

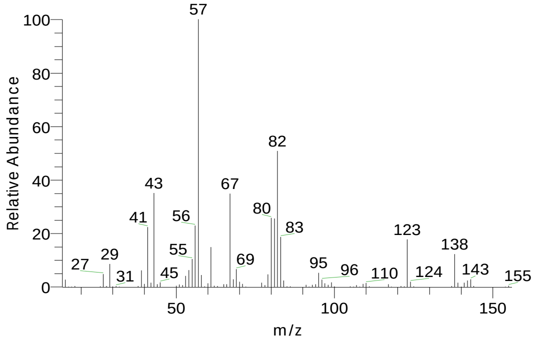

Cyclohexanol, 2-(1,1-dimethylethyl)-
Formula C10H20O, MW 156, CAS# 13491-79-7, Entry# 25713
Cyclohexanol, 2-tert-butyl-

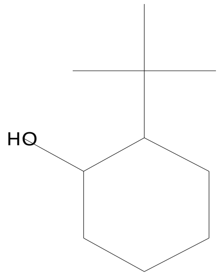

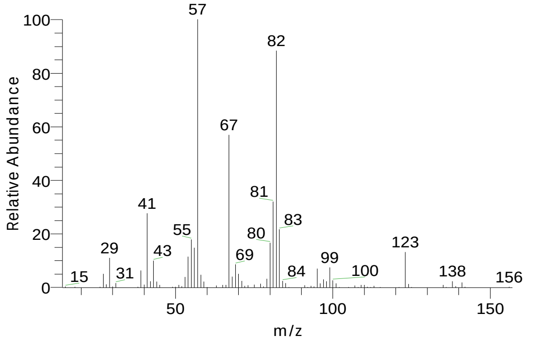

Cyclohexanol, 2-(1,1-dimethylethyl)-
Formula C10H20O, MW 156, CAS# 13491-79-7, Entry# 6483
Cyclohexanol, 2-tert-butyl-

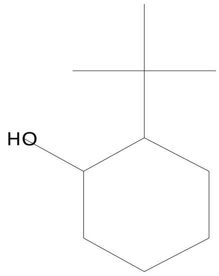

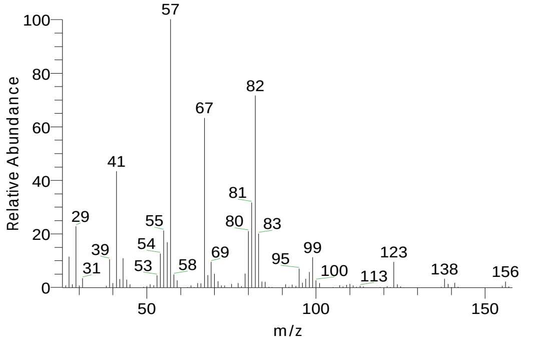

My GC-MS Report

Compound Structure | Hit Spectrum

CYCLOHEXANOL, 2-(1,1-DIMETHYLETHYL)-
Formula C10H20O, MW 156, CAS# 13491-79-7, Entry# 387741
2-(1,1-DIMETHYLETHYL)CYCLOHEXANOL

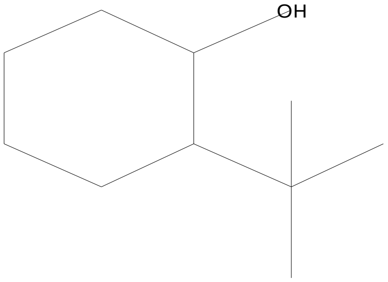

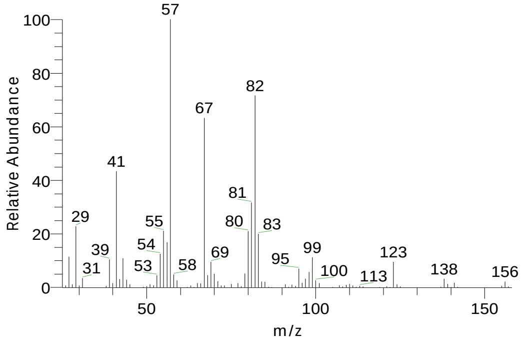

DrKarem_Tanta #3454 RT: 13.58 AV: 1 NL: 1.91E5
T: + c EI Full ms [50.000-500.000]

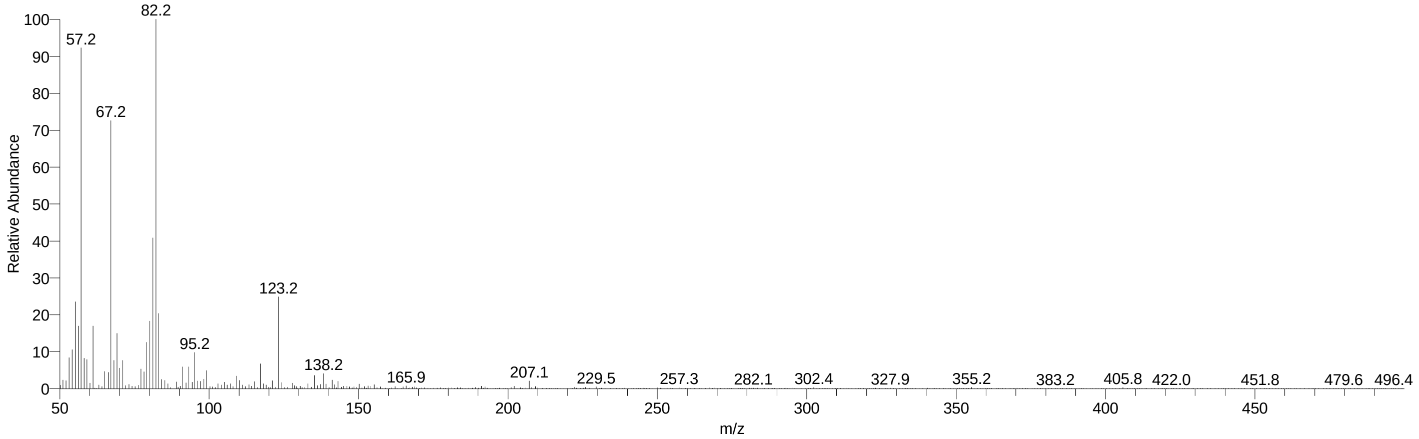

RT	Compound Name	Area %	MF	Molecular Formula	Molecular Weight	Cas #	Library
13.58	Cyclohexanol, 2-(1,1-dimethylethyl)-	0.62	849	C10H20O	156	13491-79-7	mainlib
13.58	Cyclohexanol, 2-(1,1-dimethylethyl)-	0.62	845	C10H20O	156	13491-79-7	replib
13.58	CYCLOHEXANOL, 2-(1,1-DIMETHYLETHYL)-	0.62	845	C10H20O	156	13491-79-7	WileyRegistry8e
13.58	Cyclohexanol, 2-(1,1-dimethylethyl)-	0.62	848	C10H20O	156	13491-79-7	replib
13.58	CYCLOHEXANOL, 2-(1,1-DIMETHYLETHYL)-	0.62	848	C10H20O	156	13491-79-7	WileyRegistry8e

Compound Structure | Hit Spectrum

Cyclohexanol, 2-(1,1-dimethylethyl)-
Formula C10H20O, MW 156, CAS# 13491-79-7, Entry# 25713
Cyclohexanol, 2-tert-butyl-

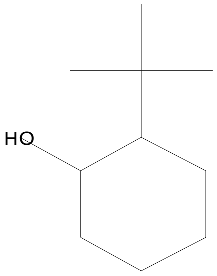

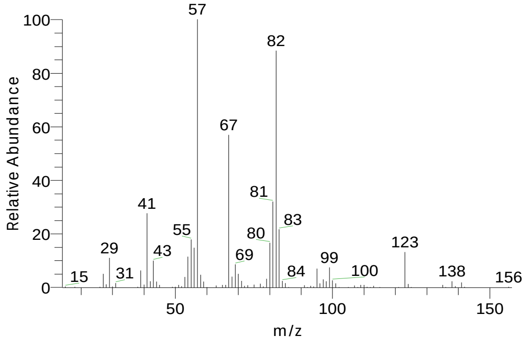

My GC-MS Report

Compound Structure | Hit Spectrum

Cyclohexanol, 2-(1,1-dimethylethyl)-
Formula C10H20O, MW 156, CAS# 13491-79-7, Entry# 6483
Cyclohexanol, 2-tert-butyl-

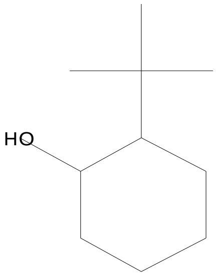

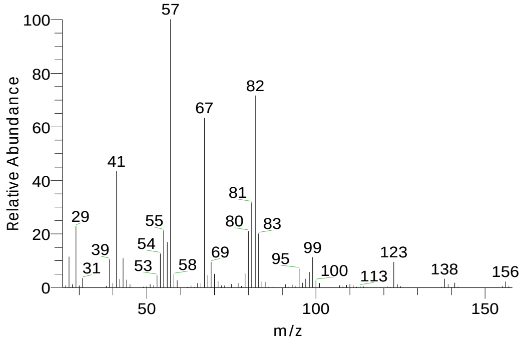

CYCLOHEXANOL, 2-(1,1-DIMETHYLETHYL)-
Formula C10H20O, MW 156, CAS# 13491-79-7, Entry# 387741
2-(1,1-DIMETHYLETHYL)CYCLOHEXANOL

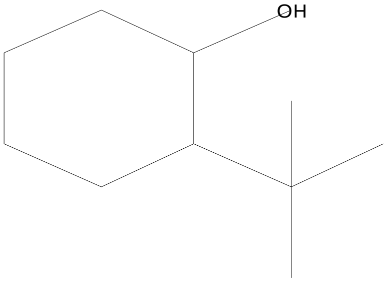

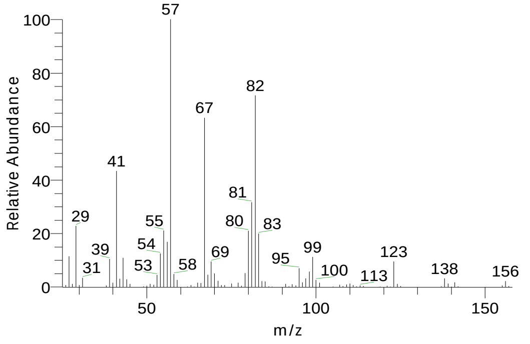

Cyclohexanol, 2-(1,1-dimethylethyl)-
Formula C10H20O, MW 156, CAS# 13491-79-7, Entry# 6482
Cyclohexanol, 2-tert-butyl-

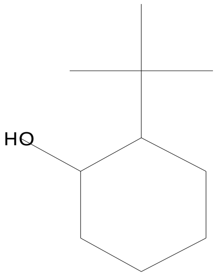

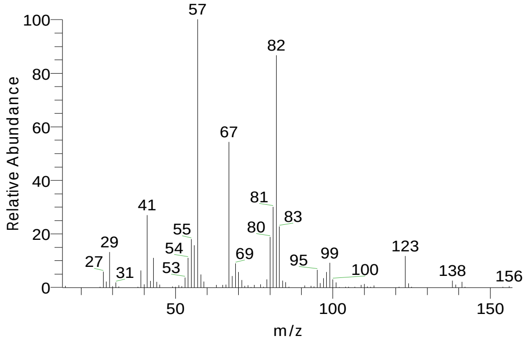

CYCLOHEXANOL, 2-(1,1-DIMETHYLETHYL)-
Formula C10H20O, MW 156, CAS# 13491-79-7, Entry# 40719
2-(1,1-DIMETHYLETHYL)CYCLOHEXANOL

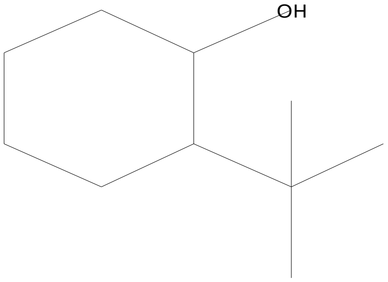

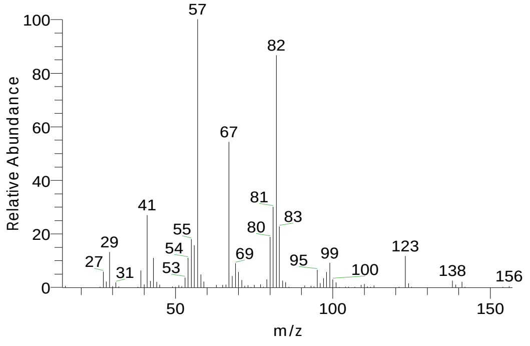

My GC-MS Report

DrKarem_Tanta #4265 RT: 16.30 AV: 1 NL: 1.16E5
T: + c EI Full ms [50.000-500.000]

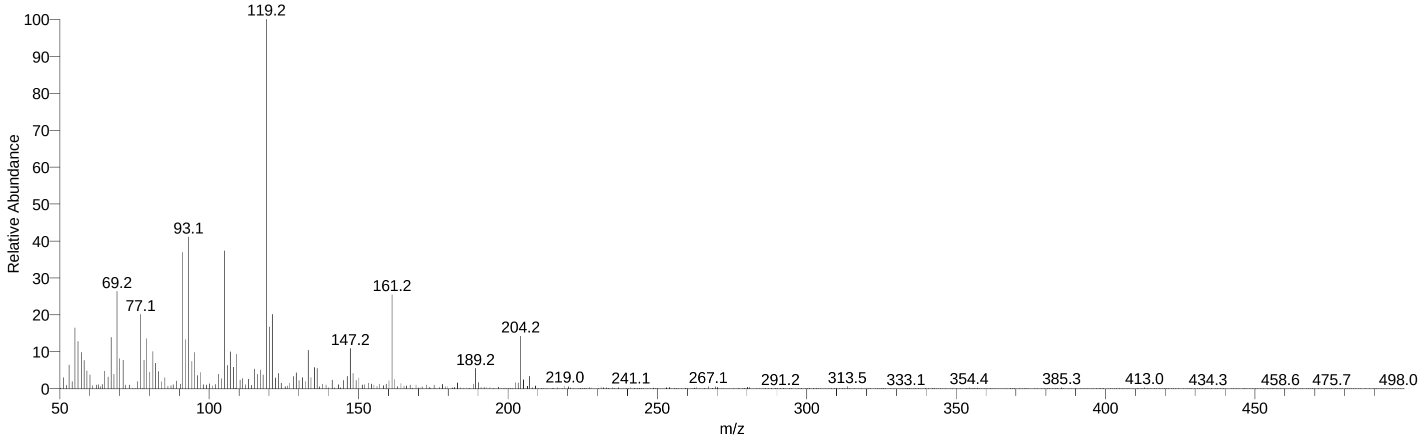

RT	Compound Name	Area %	MF	Molecular Formula	Molecular Weight	Cas #	Library
16.30	1H-3a,7-Methanoazulene, 2,3,4,7,8,8a-hexahydro-3,6,8,8-tetra methyl-, [3R-(3à,3aá,7á,8aà)]-	0.37	861	C15H24	204	469-61 -4	replib
16.30	(+)-ALPHA-CEDREN	0.37	861	C15H24	204	NA	WileyRegi stry8e
16.30	1H-3a,7-Methanoazulene, 2,3,4,7,8,8a-hexahydro-3,6,8,8-tetra methyl-, [3R-(3à,3aá,7á,8aà)]-	0.37	897	C15H24	204	469-61 -4	replib
16.30	1H-3A,7-METHANOAZULENE, 2,3,4,7,8,8A-HEXAHYDRO-3,6,8,8 -TETRAMETHYL-, [3R-(3à,3Aá,7á,8Aà)]-	0.37	897	C15H24	204	469-61 -4	WileyRegi stry8e
16.30	1H-3A,7-METHANOAZULENE, 2,3,4,7,8,8A-HEXAHYDRO-3,6,8,8 -TETRAMETHYL-, [3R-(3à,3Aá,7á,8Aà)]-	0.37	835	C15H24	204	469-61 -4	WileyRegi stry8e

Compound Structure | Hit Spectrum

1H-3a,7-Methanoazulene, 2,3,4,7,8,8a-hexahydro-3,6,8,8-tetramethyl-, [3R-(3à,3aá,7á,8aà)]-
Formula C15H24, MW 204, CAS# 469-61-4, Entry# 18651
Cedr-8-ene

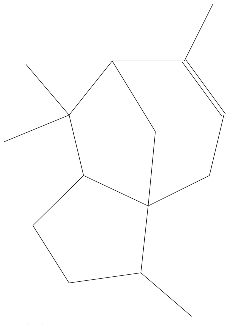

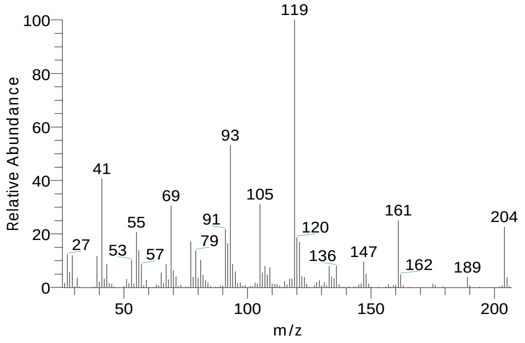

(+)-ALPHA-CEDREN
Formula C15H24, MW 204, CAS# NA, Entry# 391057
CEDR-8-ENE

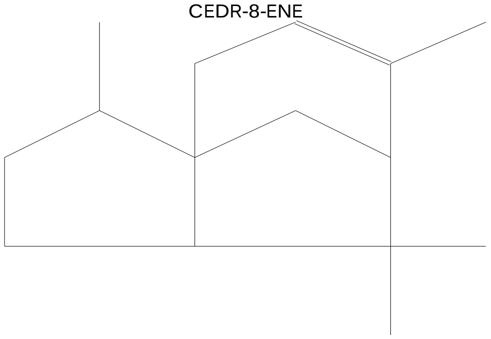

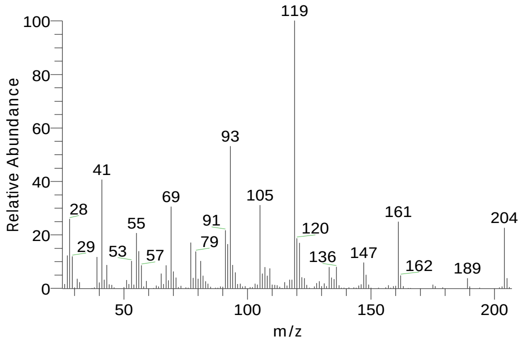

My GC-MS Report

Compound Structure | Hit Spectrum

1H-3a,7-Methanoazulene, 2,3,4,7,8,8a-hexahydro-3,6,8,8-tetramethyl-, [3R-(3à,3aá,7á,8aà)]-
Formula C15H24, MW 204, CAS# 469-61-4, Entry# 18652
Cedr-8-ene

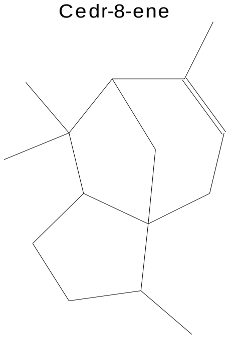

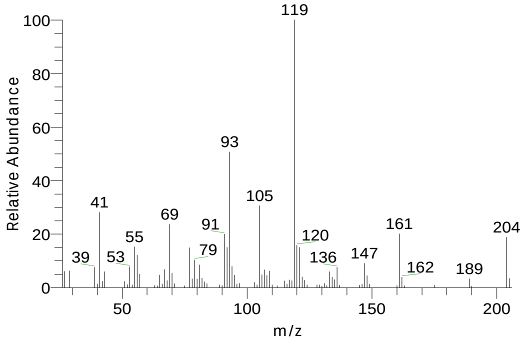

Formula C15H24, MW 204, CAS# 469-61-4, Entry# 89215
CEDR-8-ENE #

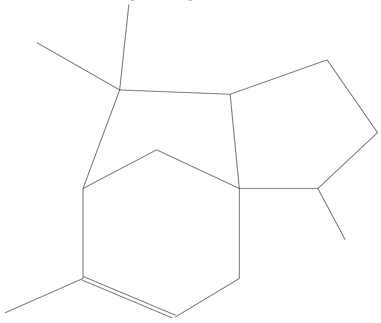

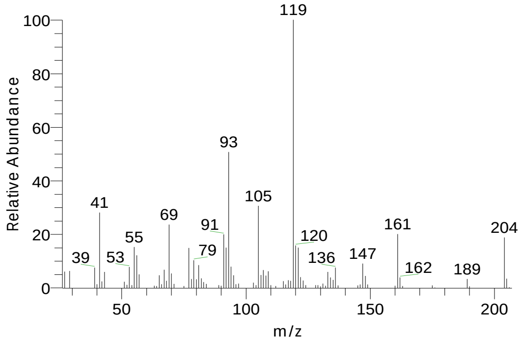

Formula C15H24, MW 204, CAS# 469-61-4, Entry# 89218
CEDR-8-ENE #

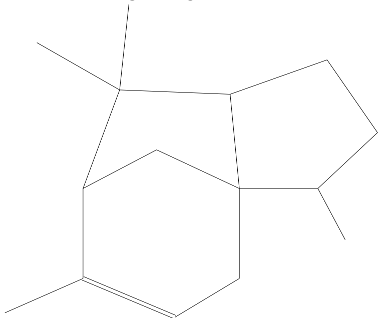

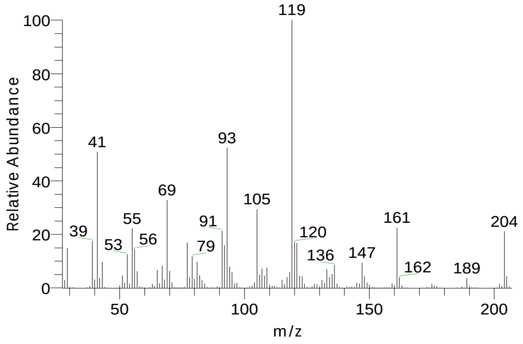

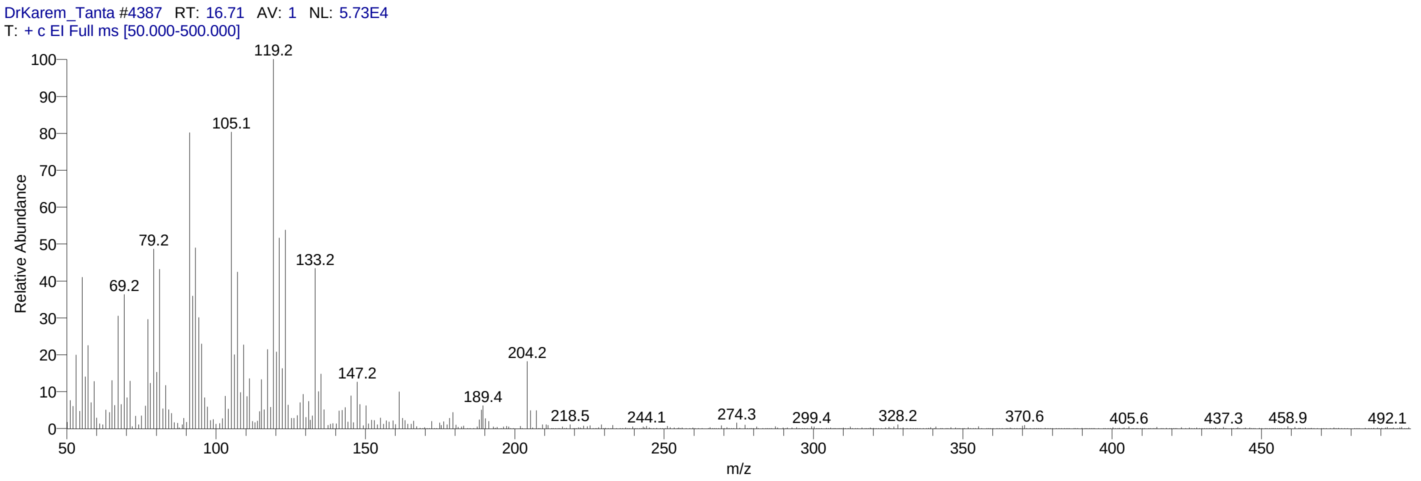

RT	Compound Name	Area %	MF	Molecular Formula	Molecular Weight	Cas #	Library
16.71	cis-Thujopsene	0.37	872	C15H24	204	470-40-6	mainlib
16.71	(-)-THUJOPSEN	0.37	880	C15H24	204	NA	WileyRegistry8e

My GC-MS Report

RT	Compound Name	Area %	MF	Molecular Formula	Molecular Weight	Cas #	Library
16.71	CYCLOPROPA[D]NAPHTHALENE, 1,1A,4,4A,5,6,7,8-OCTAHYDRO-2,4A,8,8-TETRAMETHYL-, [1AS-(1Aà,4Aá,8AR*)]-	0.37	863	C15H24	204	470-40-6	WileyRegistry8e
16.71	CYCLOPROPA[D]NAPHTHALENE, 1,1A,4,4A,5,6,7,8-OCTAHYDRO-2,4A,8,8-TETRAMETHYL-, [1AS-(1Aà,4Aá,8AR*)]-	0.37	837	C15H24	204	470-40-6	WileyRegistry8e
16.71	1H-3A,7-METHANOAZULENE, OCTAHYDRO-3,8,8-TRIMETHYL-6-METHYLENE-, [3R-(3à,3Aá,7á,8Aà)]-	0.37	827	C15H24	204	546-28-1	WileyRegistry8e

Compound Structure	Hit Spectrum

cis-Thujopsene
Formula C15H24, MW 204, CAS# 470-40-6, Entry# 100282
Thujopsene

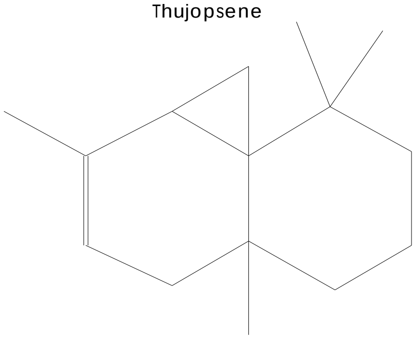

SI 815, RSI 872, mainlib, Entry# 100282, CAS# 470-40-6, cis-Thujopsene

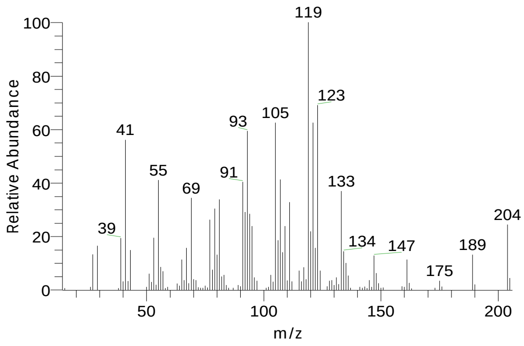

(-)-THUJOPSEN
Formula C15H24, MW 204, CAS# NA, Entry# 387961
2,4A,8,8-TETRAMETHYL-1,1A,4,4A,5,6,7,8-OCTAHYDROCYCLOPROPA[D]NAPHTHALENE

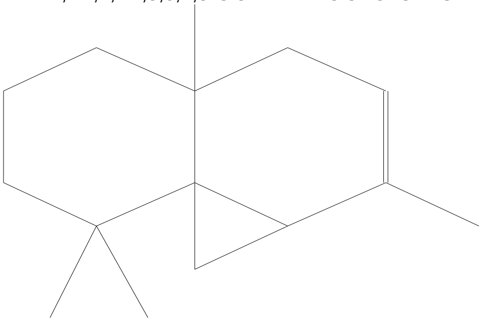

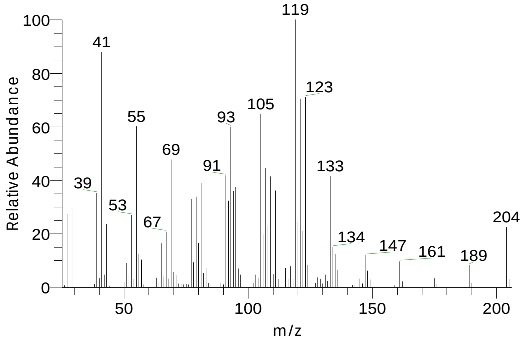

Formula C15H24, MW 204, CAS# 470-40-6, Entry# 89437
1,1,8,9A-TETRAMETHYL-2,3,5,6,7,9A-HEXAHYDRO-1H-BENZO[A]CYCLOHEPTENE #

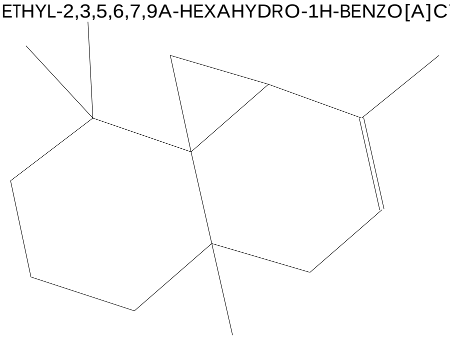

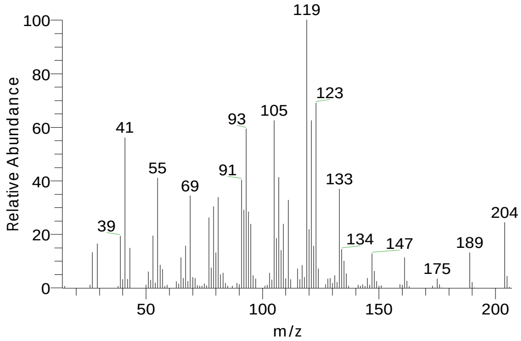

My GC-MS Report

Compound Structure | Hit Spectrum

Formula C15H24, MW 204, CAS# 470-40-6, Entry# 89435
1,1,8,9A-TETRAMETHYL-2,3,5,6,7,9A-HEXAHYDRO-1H-BENZO[A]CYCLOHEPTENE #

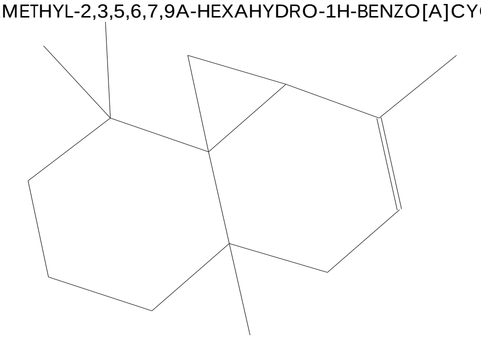

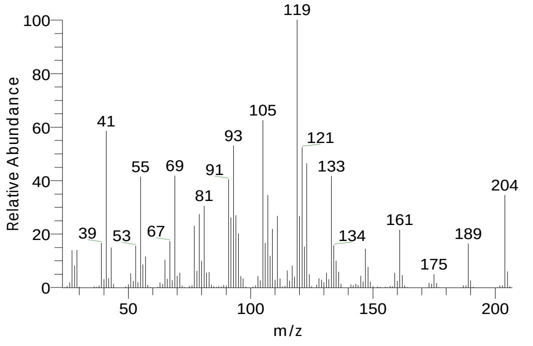

Formula C15H24, MW 204, CAS# 546-28-1, Entry# 89227
(+)-á-CEDRENE

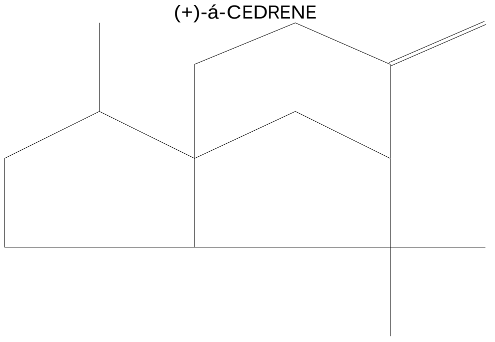

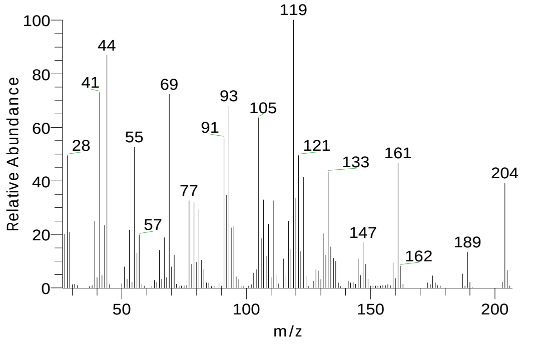

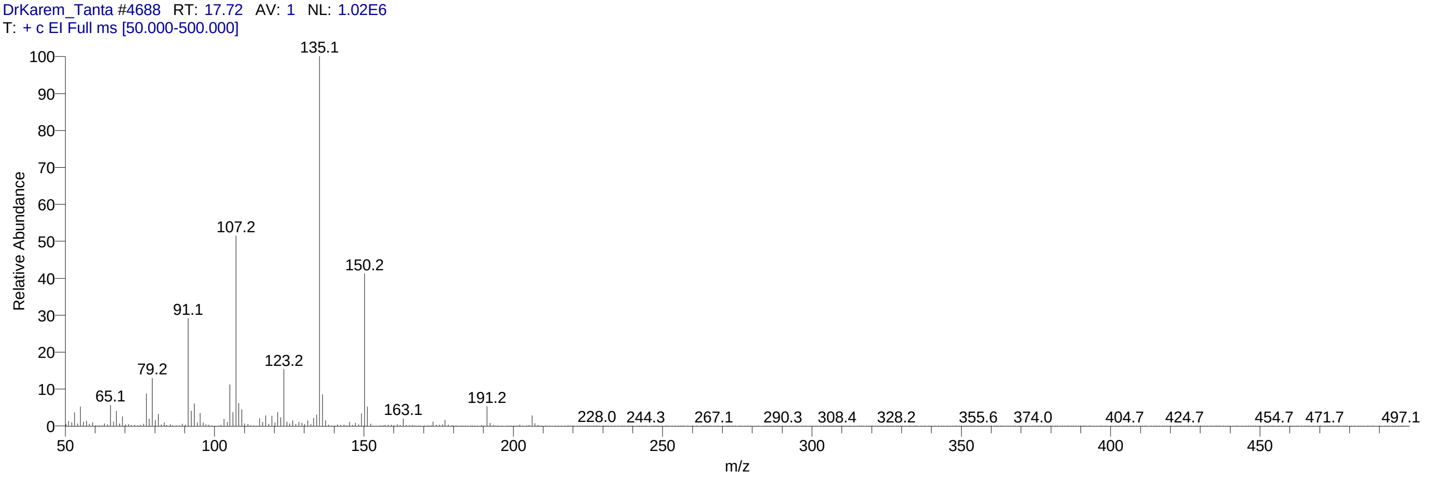

RT	Compound Name	Area %	MF	Molecular Formula	Molecular Weight	Cas #	Library
17.72	(3E)-3-METHYL-4-(2,6,6-TRIMETHYL-2-CYCLOHEXEN-1-YL)-3-BUTEN-2-ONE #	2.47	921	C14H22O	206	127-51-5	WileyRegistry8e
17.72	à Isomethyl ionone	2.47	920	C14H22O	206	127-51-5	mainlib
17.72	3-METHYL-4-(2,6,6-TRIMETHYL-2-CYCLOHEXEN-1-YL)-3-BUTEN-2-ONE	2.47	920	C14H22O	206	127-51-5	WileyRegistry8e
17.72	ç-IONONE, METHYL-	2.47	916	C14H22O	206	27417-37-4	WileyRegistry8e
17.72	ç-IONONE, METHYL-	2.47	956	C14H22O	206	27417-37-4	WileyRegistry8e

My GC-MS Report

Compound Structure | Hit Spectrum

(3E)-3-METHYL-4-(2,6,6-TRIMETHYL-2-CYCLOHEXEN-1-YL)-3-BUTEN-2-ONE #
Formula C14H22O, MW 206, CAS# 127-51-5, Entry# 91720
(3E)-3-METHYL-4-(2,6,6-TRIMETHYL-2-CYCLOHEXEN-1-YL)-3-BUTEN-2-ONE

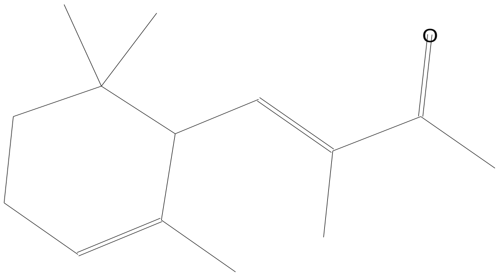

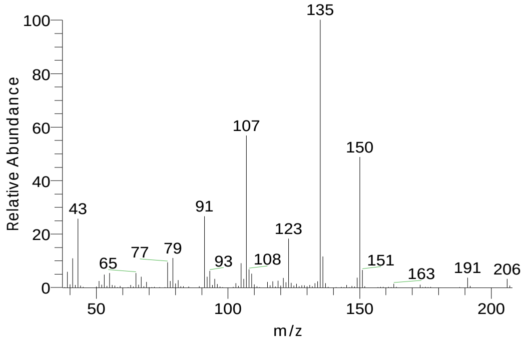

à Isomethyl ionone
Formula C14H22O, MW 206, CAS# 127-51-5, Entry# 120909
4-(2,6,6-Trimethyl 2-cyclohexen-1-yl)-3-methyl-3-buten-2-one

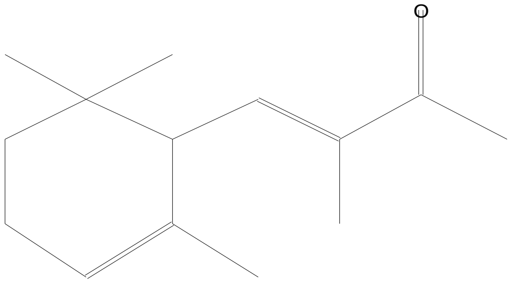

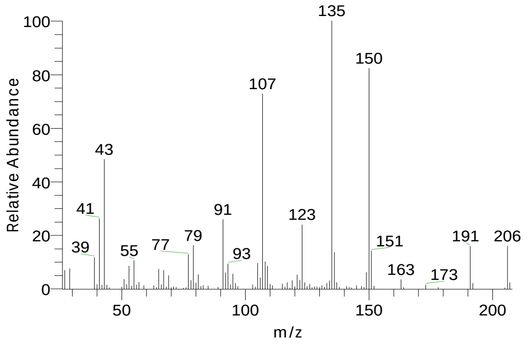

3-METHYL-4-(2,6,6-TRIMETHYL-2-CYCLOHEXEN-1-YL)-3-BUTEN-2-ONE
Formula C14H22O, MW 206, CAS# 127-51-5, Entry# 365084

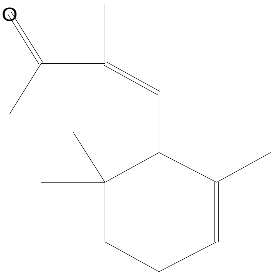

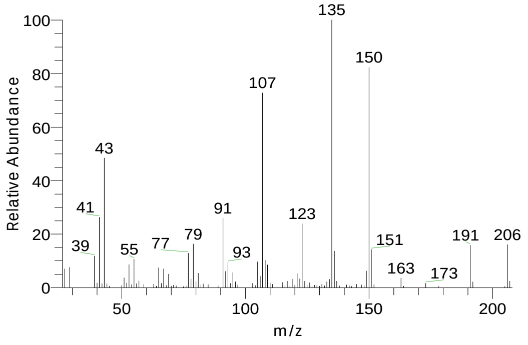

ç-IONONE, METHYL-
Formula C14H22O, MW 206, CAS# 27417-37-4, Entry# 91729
3-BUTEN-2-ONE, 4-(2,2-DIMETHYL-6-METHYLENECYCLOHEXYL)-, MONOMETHYL DERIV.

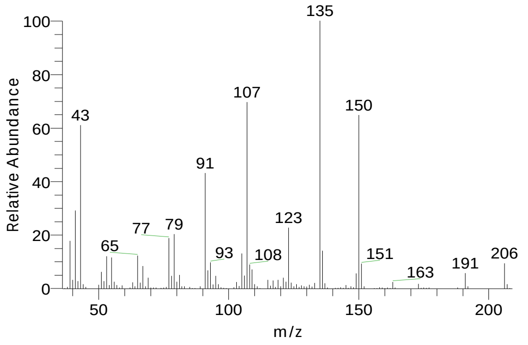

My GC-MS Report

Compound Structure | Hit Spectrum

ç-IONONE, METHYL-
Formula C14H22O, MW 206, CAS# 27417-37-4, Entry# 91728
3-BUTEN-2-ONE, 4-(2,2-DIMETHYL-6-METHYLENECYCLOHEXYL)-, MONOMETHYL DERIV.

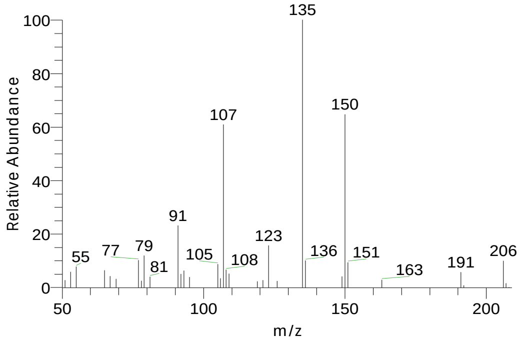

DrKarem_Tanta #4713 RT: 17.80 AV: 1 NL: 1.68E5
T: + c EI Full ms [50.000-500.000]

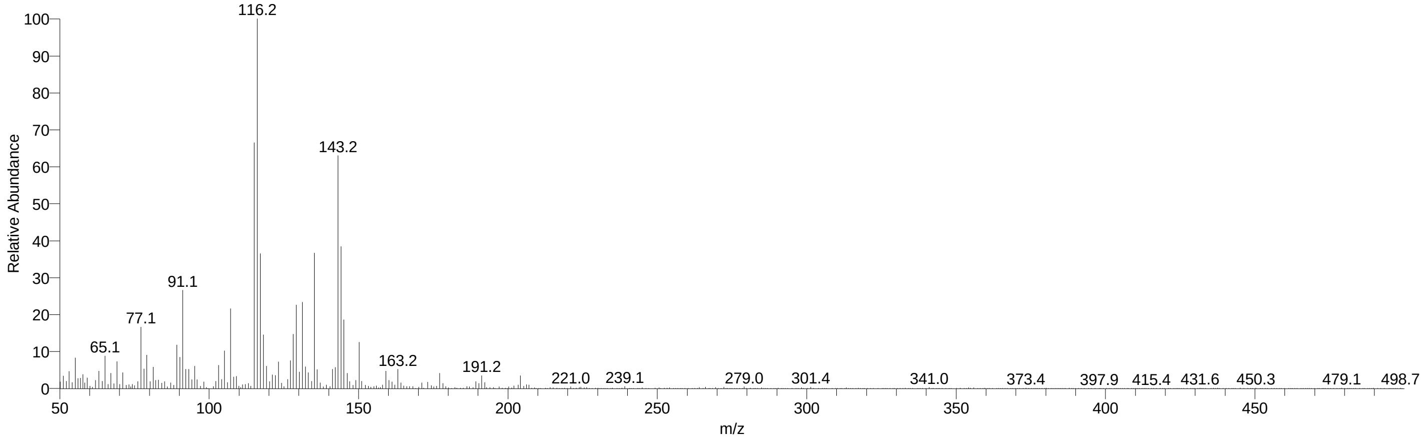

RT	Compound Name	Area %	MF	Molecular Formula	Molecular Weight	Cas #	Library
17.80	2-(3,4-DIHYDRO-1(2H)-NAPHTHALENYLIDENE)HYDRAZINECARBIMIDOTHIOIC ACID	0.62	644	C11H13N3S	219	NA	WileyRegistry8e
17.80	CYCLOHEXANONE, 2,6-DIMETHYL-4-NITRO-3-PHENYL-, (2à,3á,4á,6á)-(.+-.)-	0.62	630	C14H17NO3	247	101328-12-5	WileyRegistry8e
17.80	2,6-Dimethyl-4-nitro-3-phenyl-cyclohexanone	0.62	629	C14H17NO3	247	101328-12-5	replib
17.80	1-Trifluoroacetoxy-5-methoxy-3-phenylpentane	0.62	719	C14H17F3O3	290	NA	mainlib
17.80	1-Phenyl-1-penten-5-ol	0.62	650	C11H14O	162	37464-86-1	mainlib

Compound Structure | Hit Spectrum

2-(3,4-DIHYDRO-1(2H)-NAPHTHALENYLIDENE)HYDRAZINECARBIMIDOTHIOIC ACID
Formula C11H13N3S, MW 219, CAS# NA, Entry# 325796
NAPHTHALEN-1-ONE, 1,2,3,4-TETRAHYDRO-, ISOTHIOSEMICARBAZONE

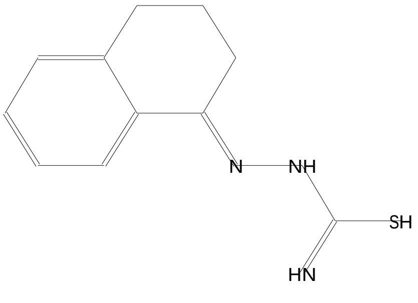

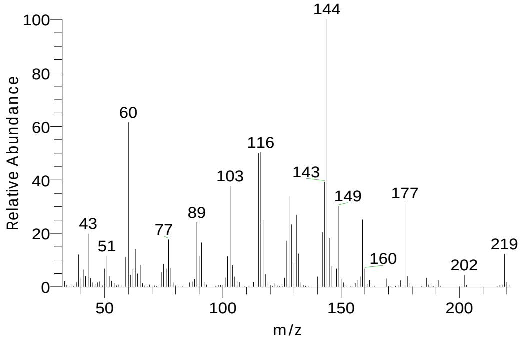

My GC-MS Report

Compound Structure | Hit Spectrum

CYCLOHEXANONE, 2,6-DIMETHYL-4-NITRO-3-PHENYL-, (2à,3á,4á,6á)-(.+-.)-
Formula C14H17NO3, MW 247, CAS# 101328-12-5, Entry# 369126
2,6-DIMETHYL-4-NITRO-3-PHENYL-CYCLOHEXANONE

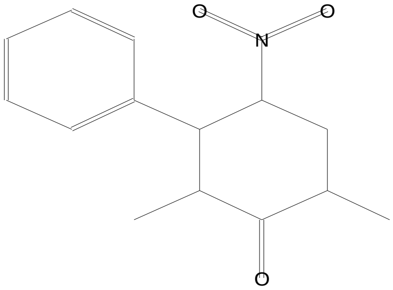

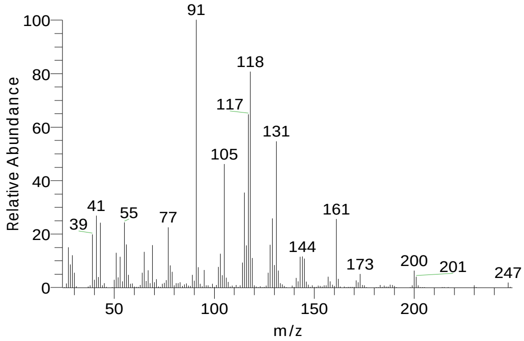

2,6-Dimethyl-4-nitro-3-phenyl-cyclohexanone
Formula C14H17NO3, MW 247, CAS# 101328-12-5, Entry# 13422
$:28FCKXGTBPFNRWQX-UHFFFAOYSA-N

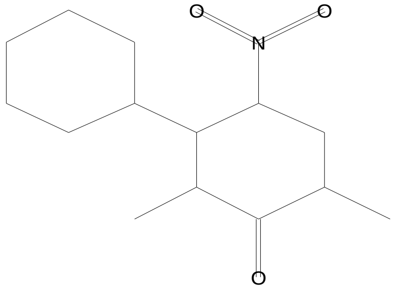

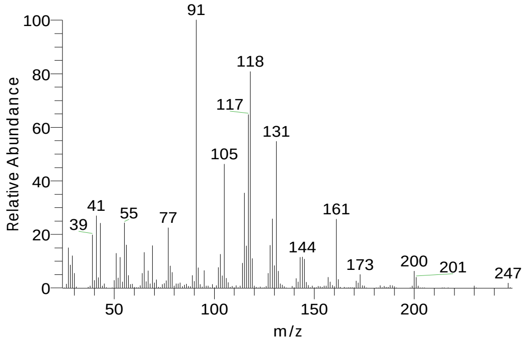

1-Trifluoroacetoxy-5-methoxy-3-phenylpentane
Formula C14H17F3O3, MW 290, CAS# NA, Entry# 17653
5-Methoxy-3-phenylpentyl trifluoroacetate #

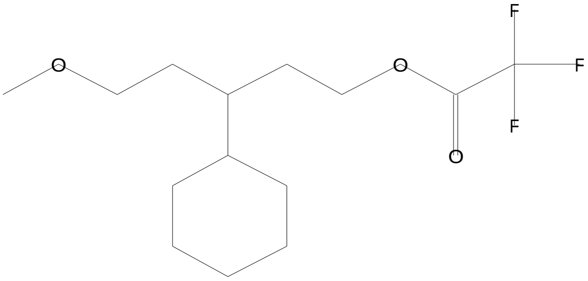

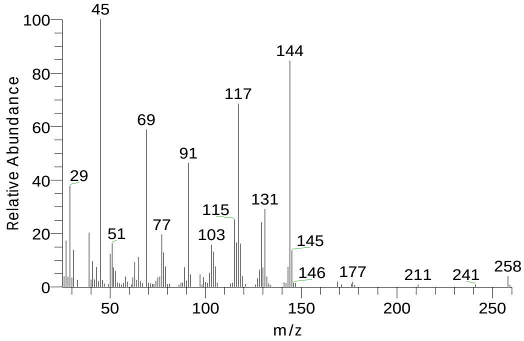

1-Phenyl-1-penten-5-ol
Formula C11H14O, MW 162, CAS# 37464-86-1, Entry# 113254
(4E)-5-Phenyl-4-penten-1-ol #

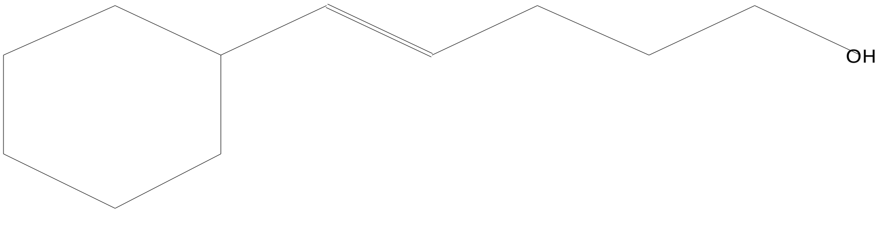

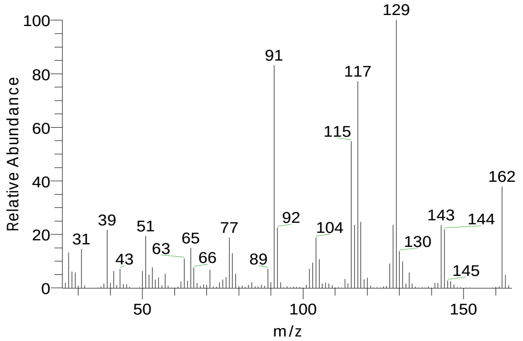

My GC-MS Report

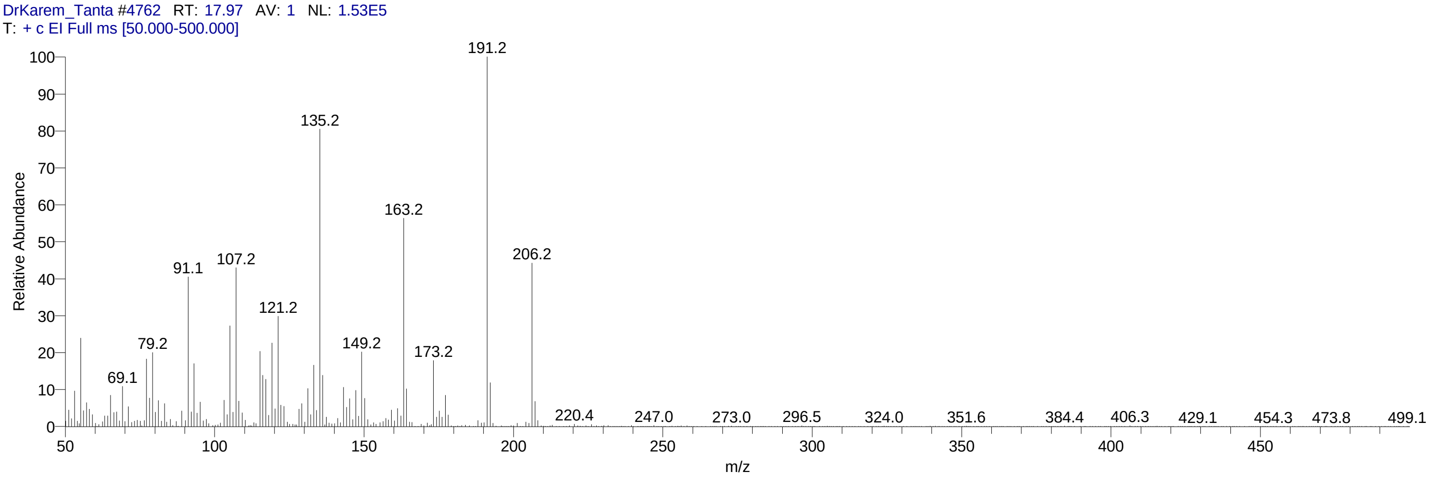

RT	Compound Name	Area %	MF	Molecular Formula	Molecular Weight	Cas #	Library
17.97	4H-Inden-4-one, 1,2,3,5,6,7-hexahydro-1,1,2,3,3-pentamethyl-	0.65	902	C14H22O	206	33704-61-9	mainlib
17.97	3-METHYL-4-(2,6,6-TRIMETHYL-1-CYCLOHEXEN-1-YL)-3-BUTEN-2-ONE	0.65	814	C14H22O	206	79-89-0	WileyRegistry8e
17.97	3-METHYL-4-(2,6,6-TRIMETHYL-1-CYCLOHEXEN-1-YL)-3-BUTEN-2-ONE	0.65	814	C14H22O	206	79-89-0	WileyRegistry8e
17.97	3-Ethyl-4,4-dimethyl-2-(2-methylpropenyl)cyclohex-2-enone	0.65	810	C14H22O	206	97452-08-9	mainlib
17.97	3-ETHYL-4,4-DIMETHYL-2-(2-METHYL-1-PROPENYL)-2-CYCLOHEXEN-1-ONE	0.65	810	C14H22O	206	97452-08-9	WileyRegistry8e

Compound Structure | Hit Spectrum

4H-Inden-4-one, 1,2,3,5,6,7-hexahydro-1,1,2,3,3-pentamethyl-
Formula C14H22O, MW 206, CAS# 33704-61-9, Entry# 177429
Cashmeran

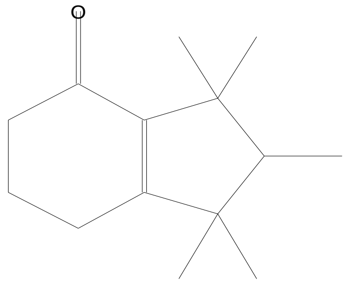

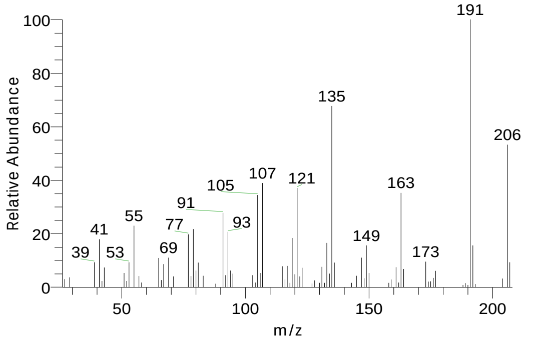

3-METHYL-4-(2,6,6-TRIMETHYL-1-CYCLOHEXEN-1-YL)-3-BUTEN-2-ONE
Formula C14H22O, MW 206, CAS# 79-89-0, Entry# 364092

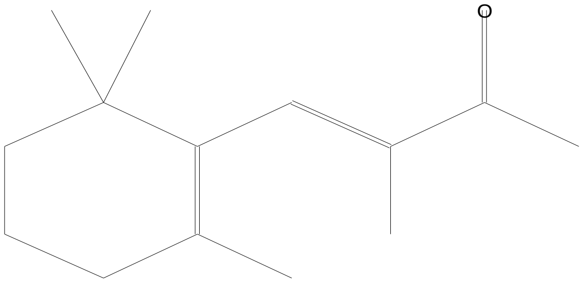

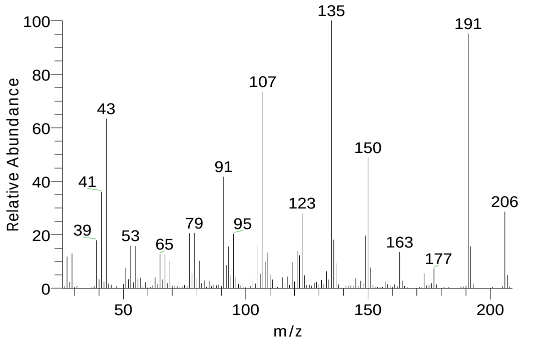

My GC-MS Report

Compound Structure | Hit Spectrum

3-METHYL-4-(2,6,6-TRIMETHYL-1-CYCLOHEXEN-1-YL)-3-BUTEN-2-ONE
Formula C14H22O, MW 206, CAS# 79-89-0, Entry# 366469
3-METHYL-4-(2,6,6-TRIMETHYL-CYCLOHEX-1-ENYL)-BUT-3-EN-2-ONE

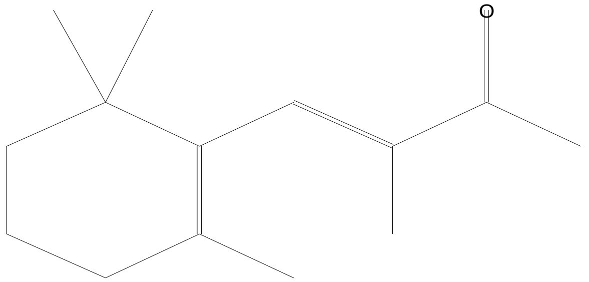

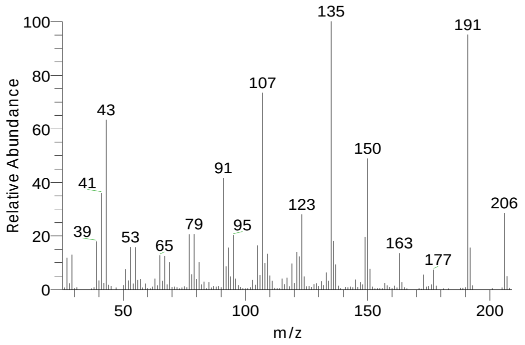

3-Ethyl-4,4-dimethyl-2-(2-methylpropenyl)cyclohex-2-enone
Formula C14H22O, MW 206, CAS# 97452-08-9, Entry# 178145
3-Ethyl-4,4-dimethyl-2-(2-methyl-1-propenyl)-2-cyclohexen-1-one #

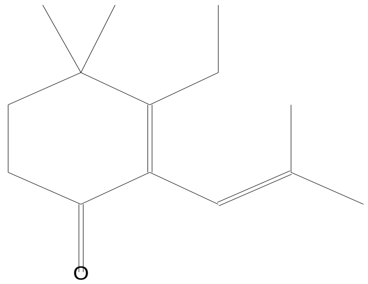

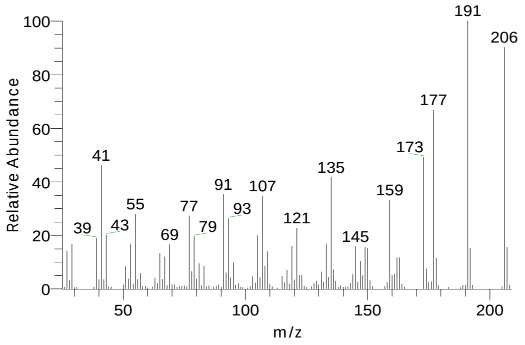

3-ETHYL-4,4-DIMETHYL-2-(2-METHYL-1-PROPENYL)-2-CYCLOHEXEN-1-ONE
Formula C14H22O, MW 206, CAS# 97452-08-9, Entry# 366470
3-ETHYL-4,4-DIMETHYL-2-(2-METHYL-PROPENYL)-CYCLOHEX-2-ENONE

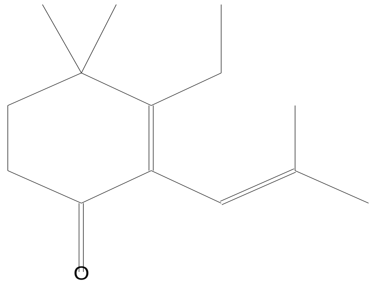

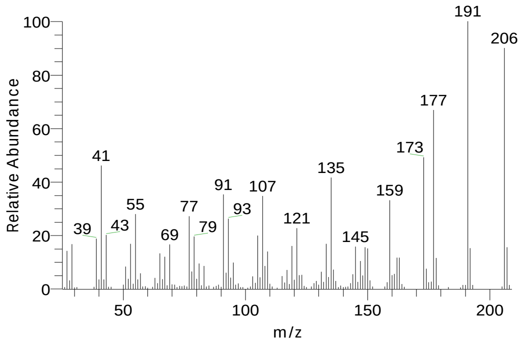

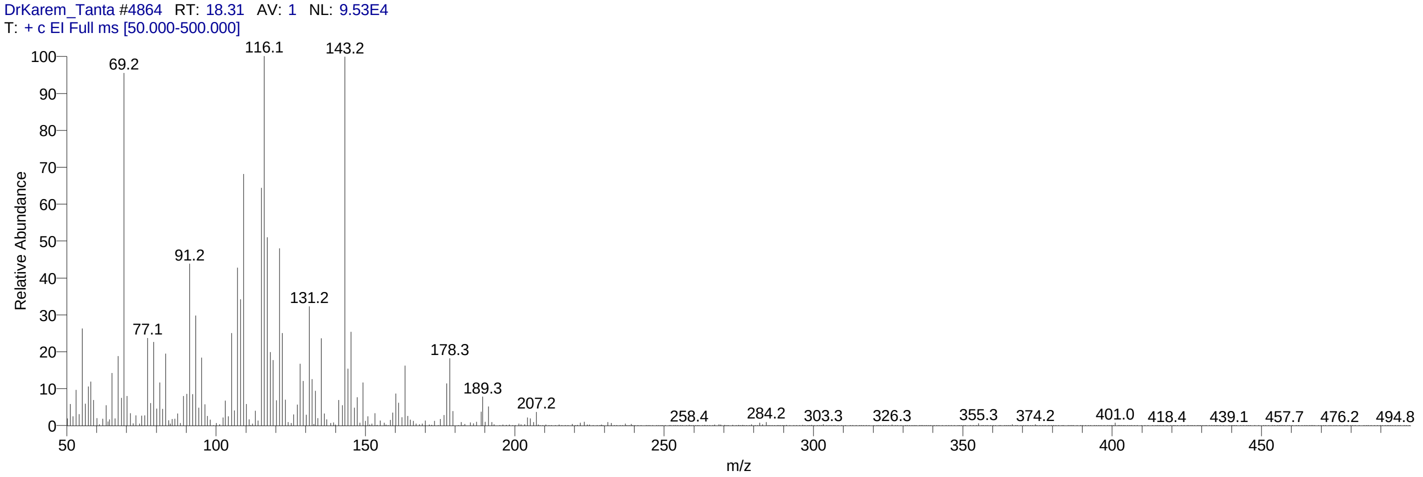

RT	Compound Name	Area %	MF	Molecular Formula	Molecular Weight	Cas #	Library
18.31	Methyl 3,5-tetradecadiynoate	0.82	687	C15H22O2	234	NA	mainlib
18.31	METHYL IONONE	0.82	704	C14H22O	206	NA	WileyRegistry8e
18.31	2,5-Dimethoxy-4-methylamphetamine	0.82	646	C12H19NO2	209	15588-95-1	nist_msms

My GC-MS Report

RT	Compound Name	Area %	MF	Molecular Formula	Molecular Weight	Cas #	Library
18.31	CYCLOISOLONGIFOL-5-OL	0.82	625	C15H24O	220	NA	WileyRegistry8e
18.31	2-Cyclopenten-1-one, 4-hydroxy-3-methyl-2-(2,4-pentadienyl)-, (Z)-(+)-	0.82	724	C11H14O2	178	487-67-2	mainlib

Compound Structure | Hit Spectrum

Methyl 3,5-tetradecadiynoate
Formula C15H22O2, MW 234, CAS# NA, Entry# 59437
$:28UQNJJXLLQHFBJO-UHFFFAOYSA-N

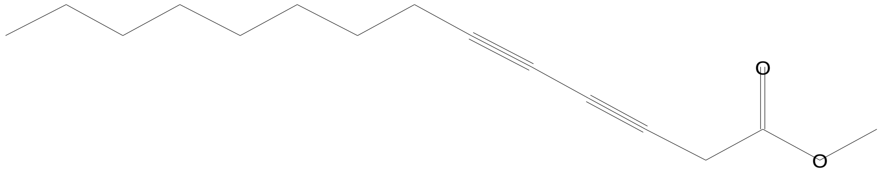

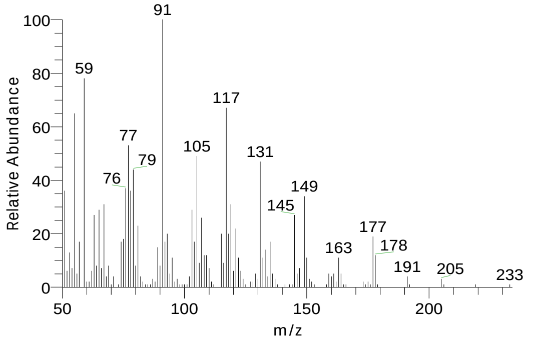

METHYL IONONE
Formula C14H22O, MW 206, CAS# NA, Entry# 91732

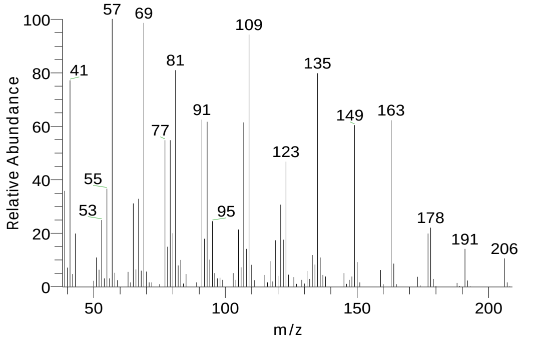

2,5-Dimethoxy-4-methylamphetamine
Formula C12H19NO2, MW 209, CAS# 15588-95-1, Entry# 185803
$:03[M+H]+

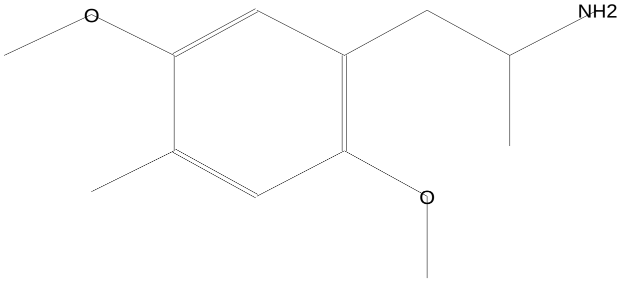

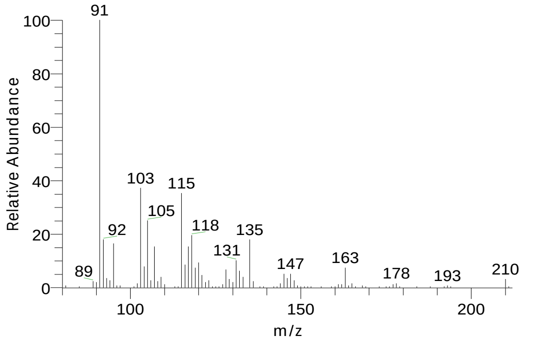

CYCLOISOLONGIFOL-5-OL
Formula C15H24O, MW 220, CAS# NA, Entry# 107403

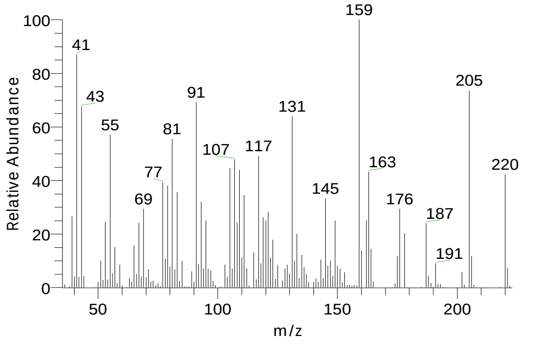

My GC-MS Report

Compound Structure | Hit Spectrum

2-Cyclopenten-1-one, 4-hydroxy-3-methyl-2-(2,4-pentadienyl)-, (Z)-(+)-
Formula C11H14O2, MW 178, CAS# 487-67-2, Entry# 119314
(+)-Pyrethrolone

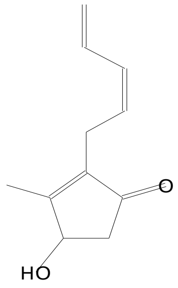

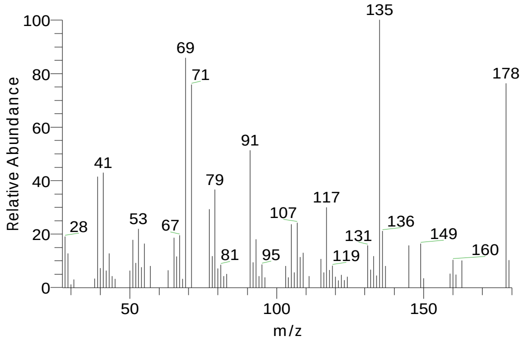

DrKarem_Tanta #4913 RT: 18.47 AV: 1 NL: 2.72E5
T: + c EI Full ms [50.000-500.000]

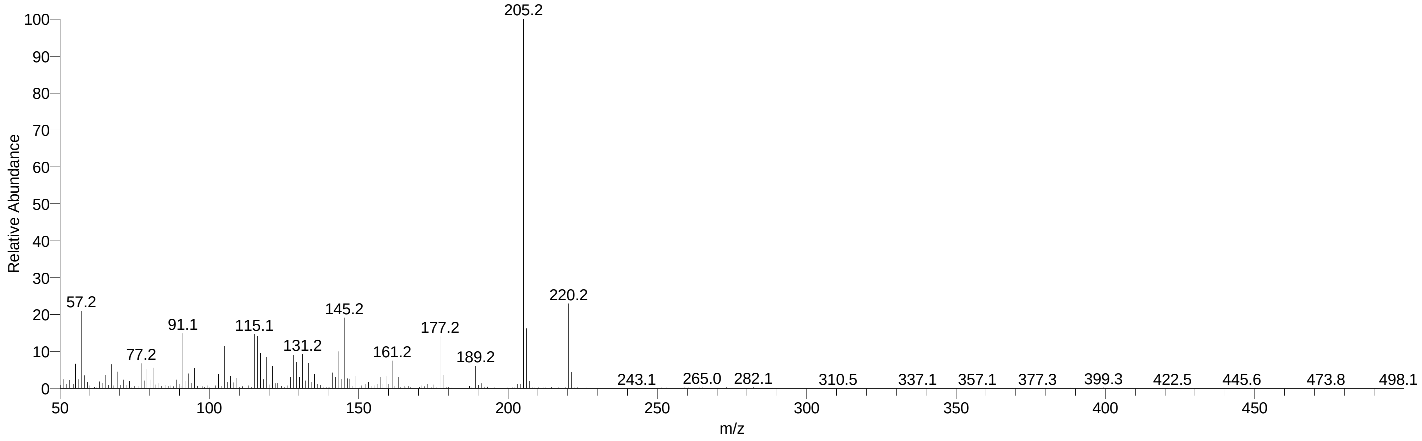

RT	Compound Name	Area %	MF	Molecular Formula	Molecular Weight	Cas #	Library
18.47	PHENOL, 2,6-BIS(1,1-DIMETHYLETHYL)-4 -METHYL-	0.71	881	C15H24O	220	128-37 -0	WileyRegi stry8e
18.47	Butylated Hydroxytoluene	0.71	873	C15H24O	220	128-37 -0	mainlib
18.47	Butylated Hydroxytoluene	0.71	872	C15H24O	220	128-37 -0	replib
18.47	PHENOL, 2,6-BIS(1,1-DIMETHYLETHYL)-4 -METHYL-	0.71	860	C15H24O	220	128-37 -0	WileyRegi stry8e
18.47	PHENOL, 2,6-BIS(1,1-DIMETHYLETHYL)-4 -METHYL-	0.71	851	C15H24O	220	128-37 -0	WileyRegi stry8e

Compound Structure | Hit Spectrum

PHENOL, 2,6-BIS(1,1-DIMETHYLETHYL)-4-METHYL-
Formula C15H24O, MW 220, CAS# 128-37-0, Entry# 107306
1-HYDROXY-4-METHYL-2,6-DI-TERT-BUTYLBENZENE

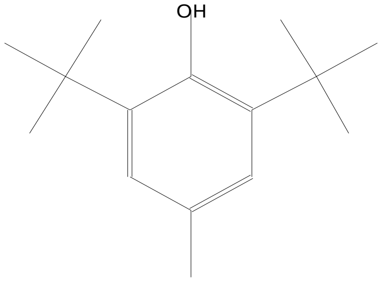

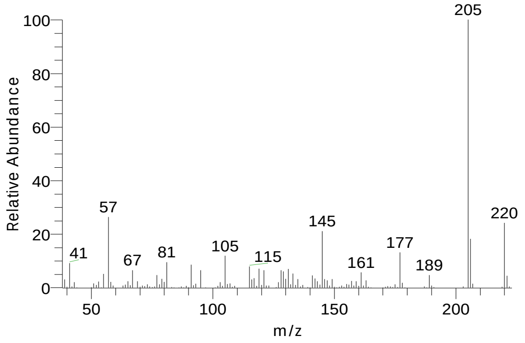

My GC-MS Report

Compound Structure | Hit Spectrum

Butylated Hydroxytoluene
Formula C15H24O, MW 220, CAS# 128-37-0, Entry# 187220
Phenol, 2,6-bis(1,1-dimethylethyl)-4-methyl-

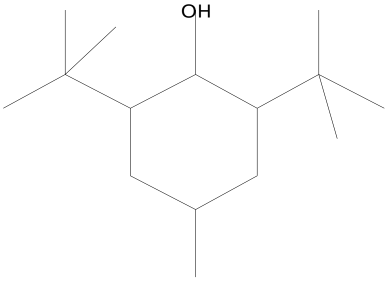

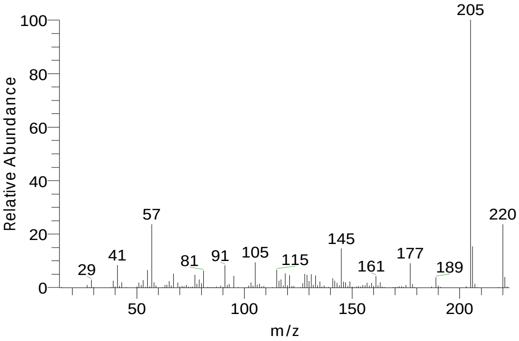

Butylated Hydroxytoluene
Formula C15H24O, MW 220, CAS# 128-37-0, Entry# 29004
Phenol, 2,6-bis(1,1-dimethylethyl)-4-methyl-

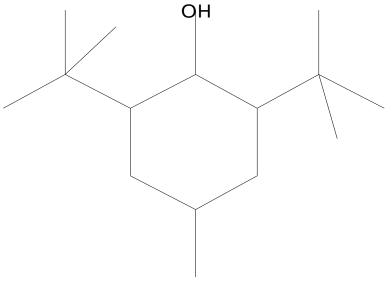

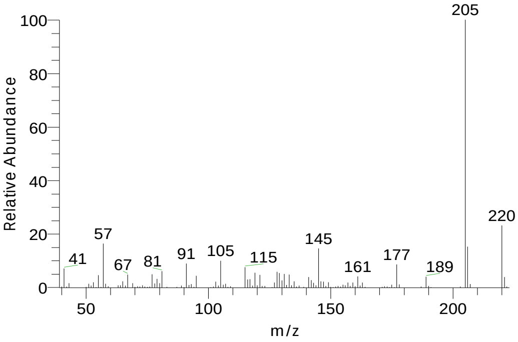

PHENOL, 2,6-BIS(1,1-DIMETHYLETHYL)-4-METHYL-
Formula C15H24O, MW 220, CAS# 128-37-0, Entry# 107054
1-HYDROXY-4-METHYL-2,6-DI-TERT-BUTYLBENZENE

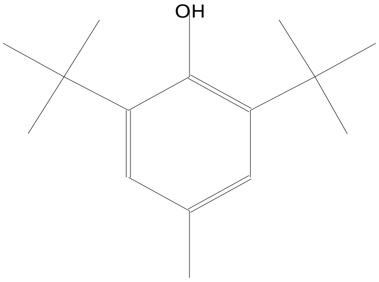

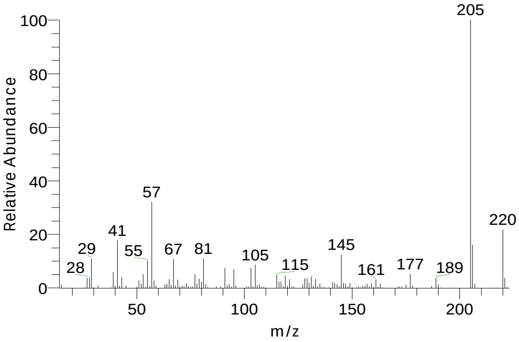

PHENOL, 2,6-BIS(1,1-DIMETHYLETHYL)-4-METHYL-
Formula C15H24O, MW 220, CAS# 128-37-0, Entry# 107058
1-HYDROXY-4-METHYL-2,6-DI-TERT-BUTYLBENZENE

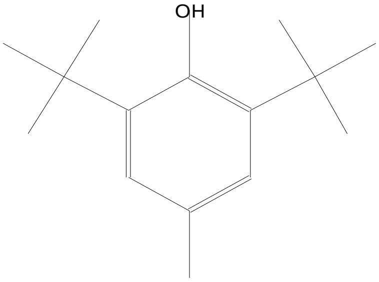

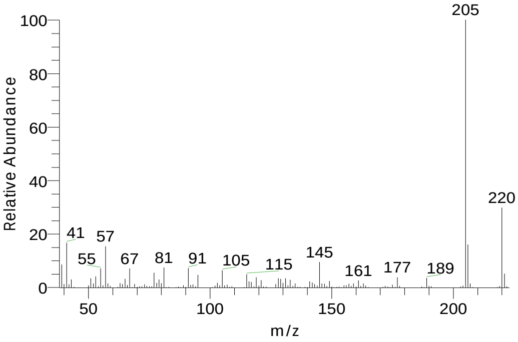

My GC-MS Report

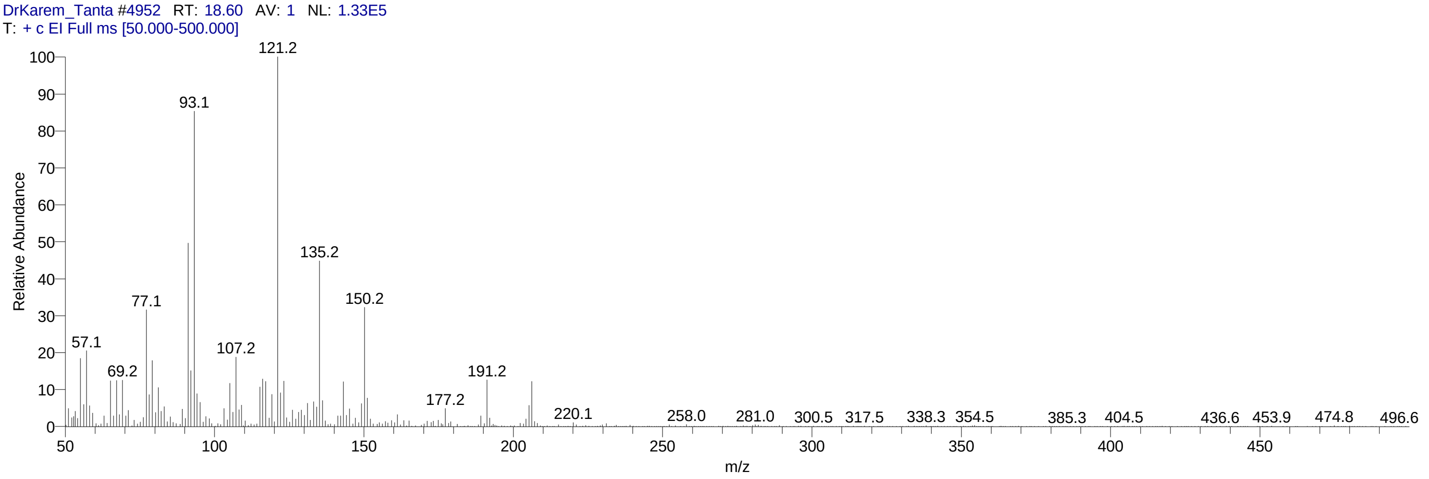

RT	Compound Name	Area %	MF	Molecular Formula	Molecular Weight	Cas #	Library
18.60	ç ISO METHYL IONONE	0.49	825	C14H22O	206	7388-22-9	WileyRegistry8e
18.60	(R-(E))-1-(2,6,6-TRIMETHYL-2-CYCLOHEXEN-1-YL)PENT-1-EN-3-ONE	0.49	820	C14H22O	206	127-42-4	WileyRegistry8e
18.60	1-Penten-3-one, 1-(2,6,6-trimethyl-2-cyclohexen-1-yl)-	0.49	811	C14H22O	206	7779-30-8	mainlib
18.60	1-Penten-3-one, 1-(2,6,6-trimethyl-2-cyclohexen-1-yl)-, (E)-	0.49	850	C14H22O	206	93302-56-8	mainlib
18.60	1-PENTEN-3-ONE, 1-(2,6,6-TRIMETHYL-2-CYCLOHEXEN-1-YL)-, (E)-	0.49	850	C14H22O	206	93302-56-8	WileyRegistry8e

Compound Structure	Hit Spectrum

ç ISO METHYL IONONE
Formula C14H22O, MW 206, CAS# 7388-22-9, Entry# 91730
3-BUTEN-2-ONE, 4-(2,2-DIMETHYL-6-METHYLENECYCLOHEXYL)-3-METHYL-

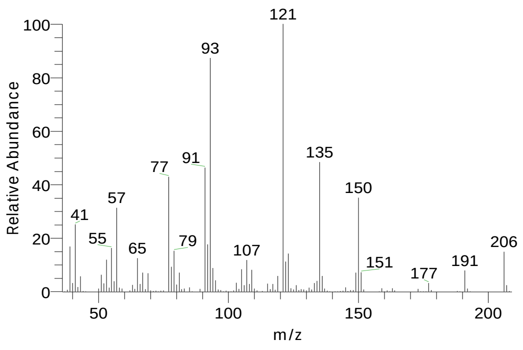

(R-(E))-1-(2,6,6-TRIMETHYL-2-CYCLOHEXEN-1-YL)PENT-1-EN-3-ONE
Formula C14H22O, MW 206, CAS# 127-42-4, Entry# 91731
à-METHYL IONONE

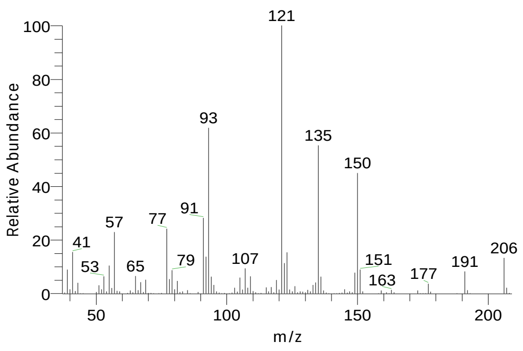

My GC-MS Report

Compound Structure | Hit Spectrum

1-Penten-3-one, 1-(2,6,6-trimethyl-2-cyclohexen-1-yl)-
Formula C14H22O, MW 206, CAS# 7779-30-8, Entry# 102833
1-(2,6,6-Trimethylcyclohex-2-en-1-yl)pent-1-en-3-one

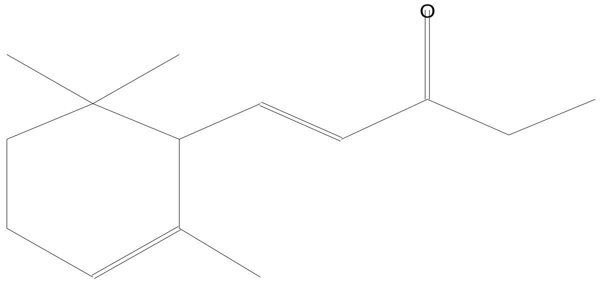

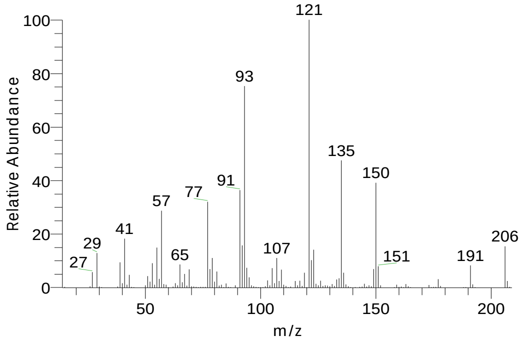

1-Penten-3-one, 1-(2,6,6-trimethyl-2-cyclohexen-1-yl)-, (E)-
Formula C14H22O, MW 206, CAS# 93302-56-8, Entry# 102842
(1E)-1-(2,6,6-Trimethyl-2-cyclohexen-1-yl)-1-penten-3-one #

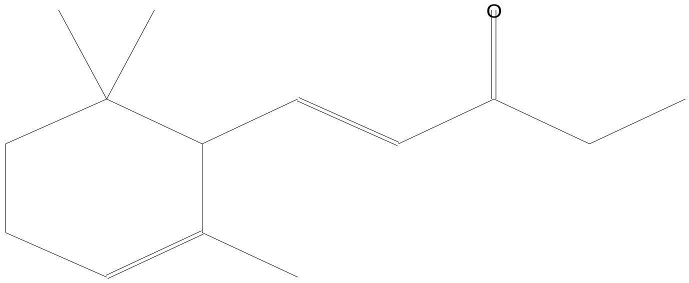

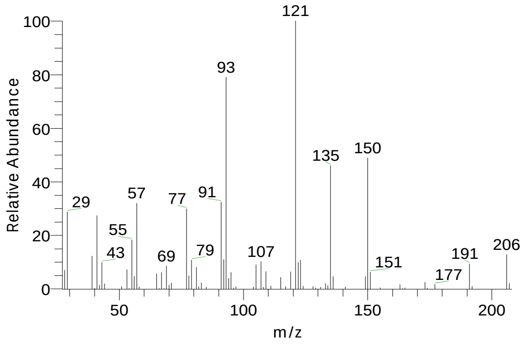

1-PENTEN-3-ONE, 1-(2,6,6-TRIMETHYL-2-CYCLOHEXEN-1-YL)-, (E)-
Formula C14H22O, MW 206, CAS# 93302-56-8, Entry# 379873
(1E)-1-(2,6,6-TRIMETHYL-2-CYCLOHEXEN-1-YL)-1-PENTEN-3-ONE #

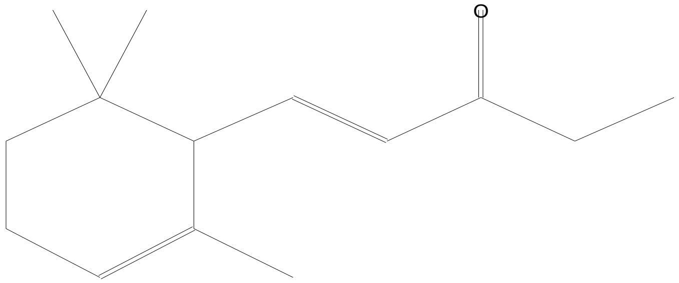

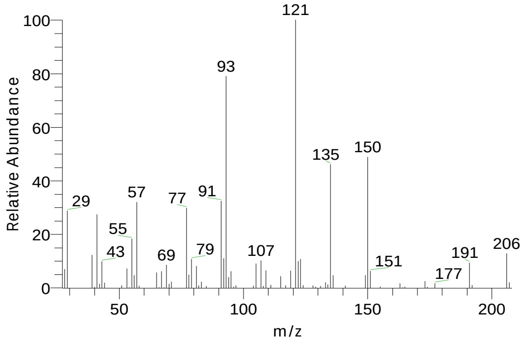

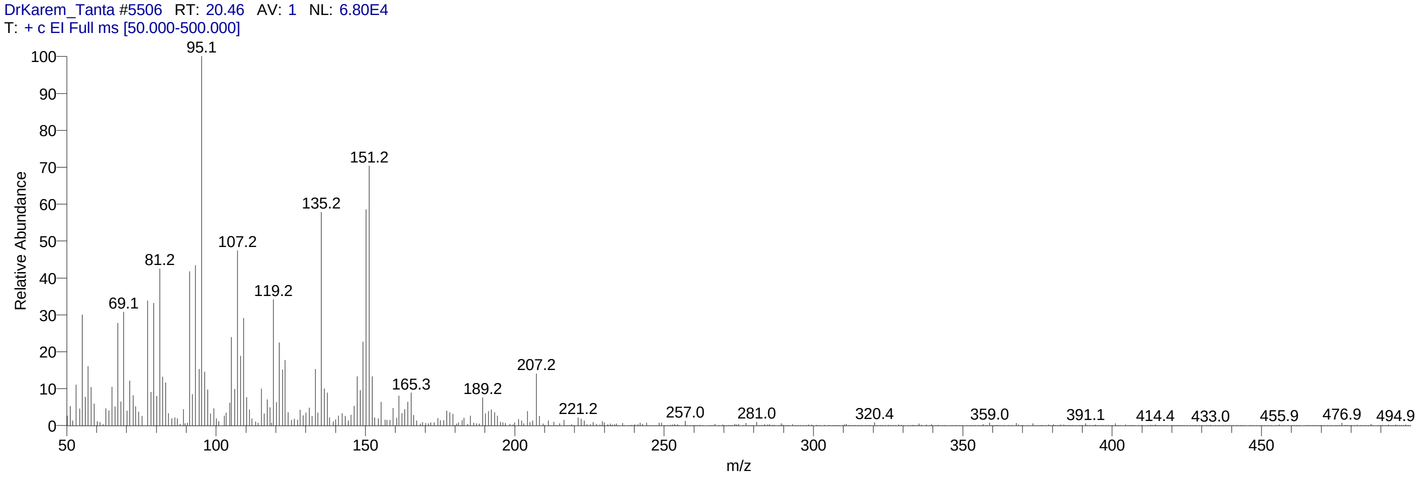

RT	Compound Name	Area %	MF	Molecular Formula	Molecular Weight	Cas #	Library
20.46	1H-3A,7-METHANOAZULEN-5-OL, OCTAHYDRO-3,8,8-TRIMETHYL-6-METHYLENE-	0.44	851	C15H24O	220	28231-03-0	WileyRegistry8e

My GC-MS Report

RT	Compound Name	Area %	MF	Molecular Formula	Molecular Weight	Cas #	Library
20.46	1H-3A,7-METHANOAZULEN-6-OL, OCTAHYDRO-3,6,8,8-TETRAMETHYL-, [3R-(3à,3Aá,6à,7á,8Aà)]-	0.44	855	C15H26O	222	77-53-2	WileyRegistry8e
20.46	Cedrol	0.44	834	C15H26O	222	77-53-2	replib
20.46	1H-3A,7-METHANOAZULEN-6-OL, OCTAHYDRO-3,6,8,8-TETRAMETHYL-, [3R-(3à,3Aá,6à,7á,8Aà)]-	0.44	832	C15H26O	222	77-53-2	WileyRegistry8e
20.46	Cedrol	0.44	830	C15H26O	222	77-53-2	replib

Compound Structure | Hit Spectrum

1H-3A,7-METHANOAZULEN-5-OL, OCTAHYDRO-3,8,8-TRIMETHYL-6-METHYLENE-
Formula C15H24O, MW 220, CAS# 28231-03-0, Entry# 107149
1H-3A,7-METHANOAZULEN-5-OL, OCTAHYDRO-6-METHYLENE-3,8,8-TRIMETHYL-

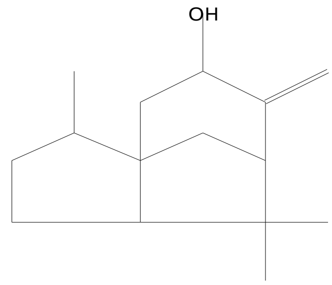

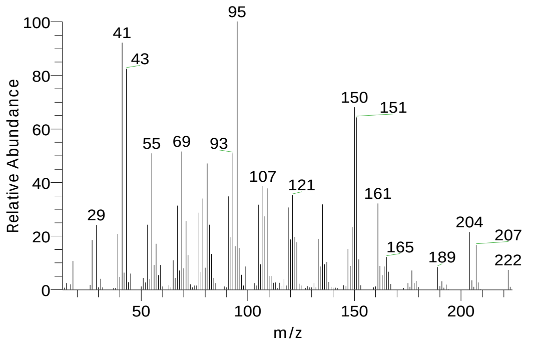

Formula C15H26O, MW 222, CAS# 77-53-2, Entry# 109783
CEDRAN-8-OL #

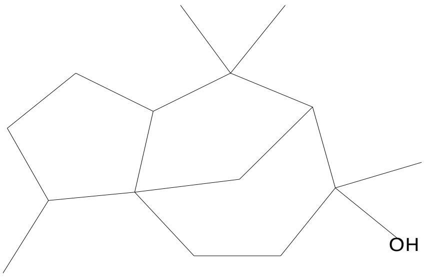

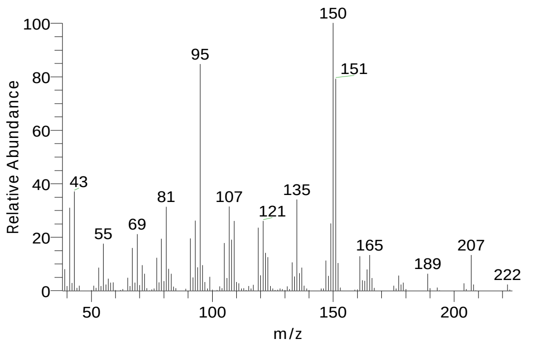

Cedrol
Formula C15H26O, MW 222, CAS# 77-53-2, Entry# 14631
1H-3a,7-Methanoazulen-6-ol, octahydro-3,6,8,8-tetramethyl-, [3R-(3à,3aá,6à,7á,8aà)]-

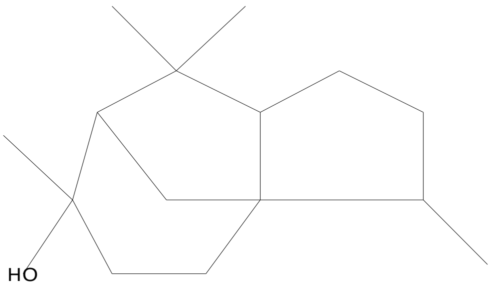

SI 796, RSI 834, replib, Entry# 14631, CAS# 77-53-2, Cedrol

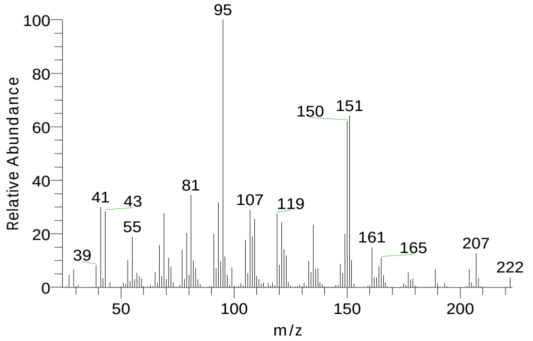

My GC-MS Report

Compound Structure | Hit Spectrum

Formula C15H26O, MW 222, CAS# 77-53-2, Entry# 109786
CEDRAN-8-OL #

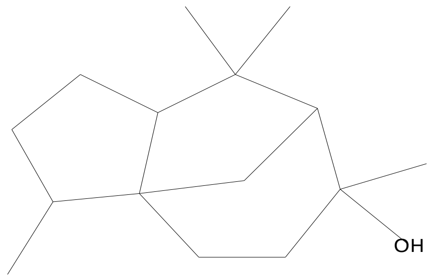

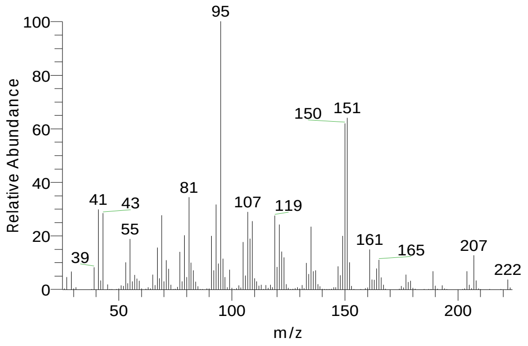

SI 787, RSI 830, replib, Entry# 14630, CAS# 77-53-2, Cedrol

Cedrol
Formula C15H26O, MW 222, CAS# 77-53-2, Entry# 14630
1H-3a,7-Methanoazulen-6-ol, octahydro-3,6,8,8-tetramethyl-, [3R-(3à,3aá,6à,7á,8aà)]-

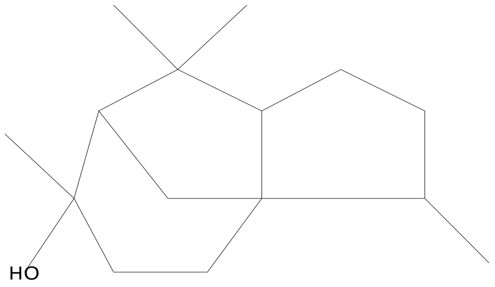

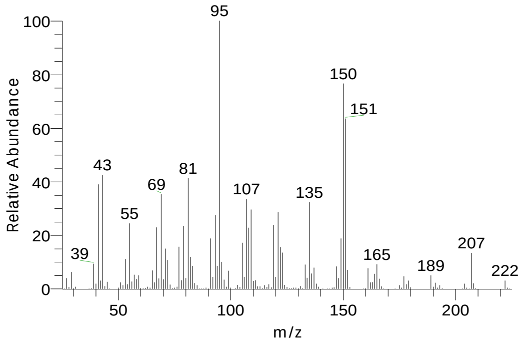

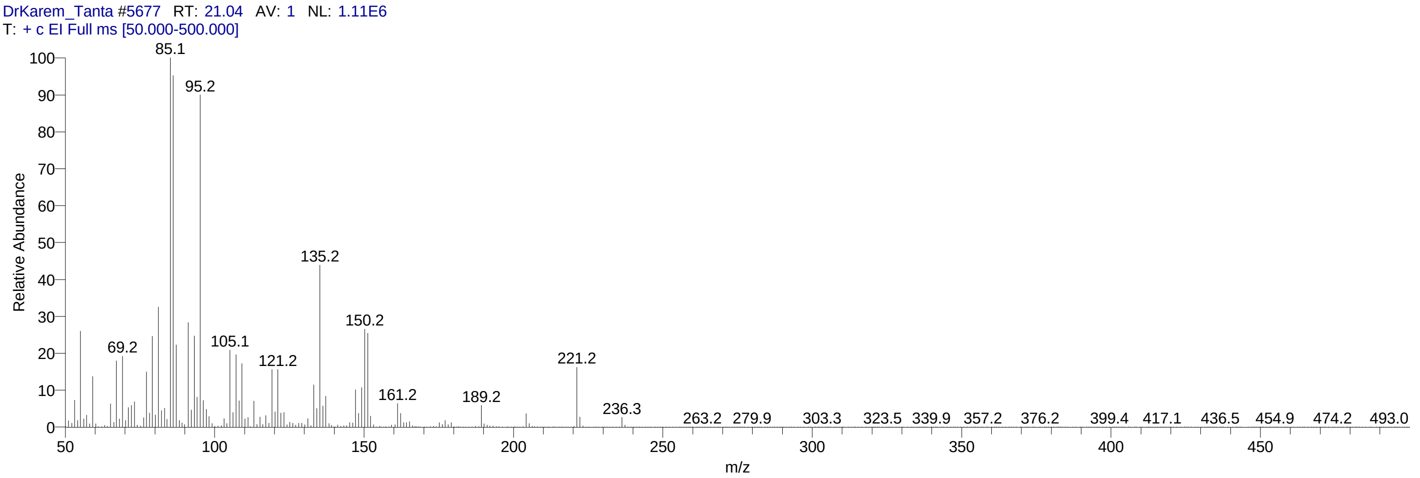

RT	Compound Name	Area %	MF	Molecular Formula	Molecular Weight	Cas #	Library
21.04	Epicedrol	6.42	786	C15H26O	222	NA	mainlib
21.04	CEDRAN-8-OL	6.42	786	C15H26O	222	NA	WileyRegistry8e
21.04	Cedrol	6.42	734	C15H26O	222	77-53-2	replib
21.04	1H-3A,7-METHANOAZULEN-6-OL, OCTAHYDRO-3,6,8,8-TETRAMETHYL-, [3R-(3à,3Aá,6à,7á,8Aà)]-	6.42	734	C15H26O	222	77-53-2	WileyRegistry8e
21.04	1H-3A,7-METHANOAZULEN-5-OL, OCTAHYDRO-3,8,8-TRIMETHYL-6-METHYLENE-	6.42	778	C15H24O	220	28231-03-0	WileyRegistry8e

My GC-MS Report

Compound Structure | Hit Spectrum

Epicedrol
Formula C15H26O, MW 222, CAS# NA, Entry# 68752
Cedran-8-ol #

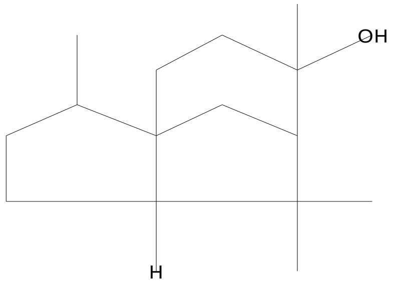

SI 719, RSI 786, mainlib, Entry# 68752, CAS# NA, Epicedrol

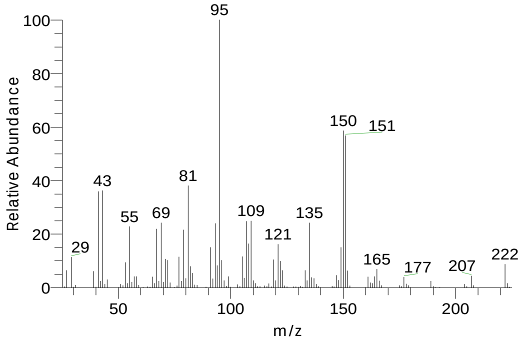

CEDRAN-8-OL
Formula C15H26O, MW 222, CAS# NA, Entry# 388201
EPICEDROL

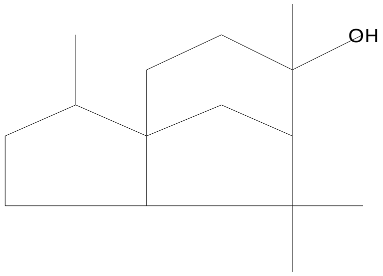

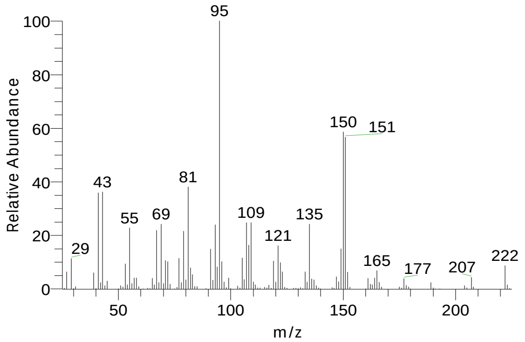

Cedrol
Formula C15H26O, MW 222, CAS# 77-53-2, Entry# 14631
1H-3a,7-Methanoazulen-6-ol, octahydro-3,6,8,8-tetramethyl-, [3R-(3à,3aá,6à,7á,8aà)]-

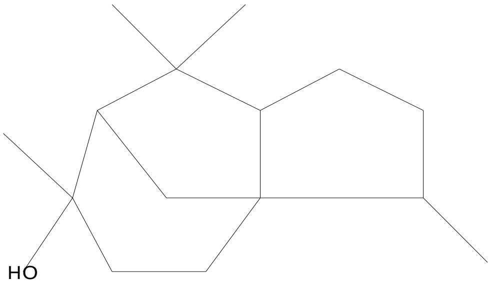

SI 717, RSI 734, replib, Entry# 14631, CAS# 77-53-2, Cedrol

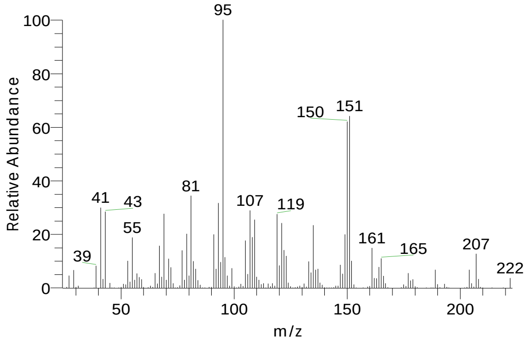

Formula C15H26O, MW 222, CAS# 77-53-2, Entry# 109786
CEDRAN-8-OL #

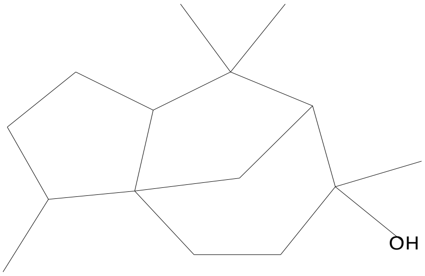

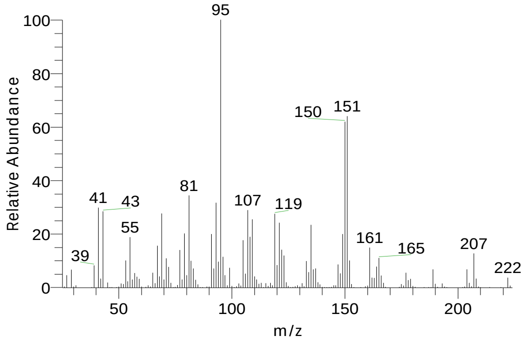

My GC-MS Report

Compound Structure | Hit Spectrum

1H-3A,7-METHANOAZULEN-5-OL, OCTAHYDRO-3,8,8-TRIMETHYL-6-METHYLENE-
Formula C15H24O, MW 220, CAS# 28231-03-0, Entry# 107149
1H-3A,7-METHANOAZULEN-5-OL, OCTAHYDRO-6-METHYLENE-3,8,8-TRIMETHYL-

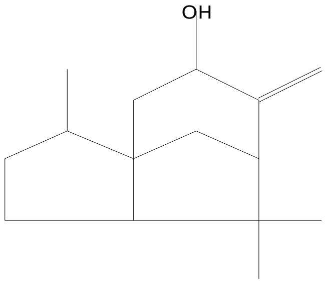

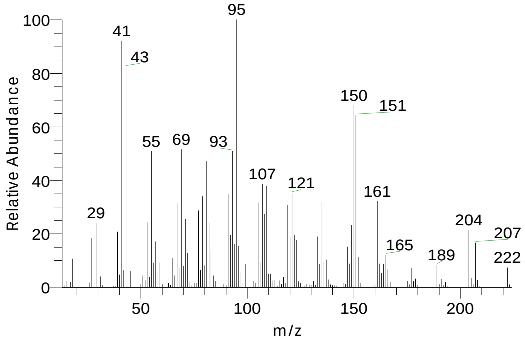

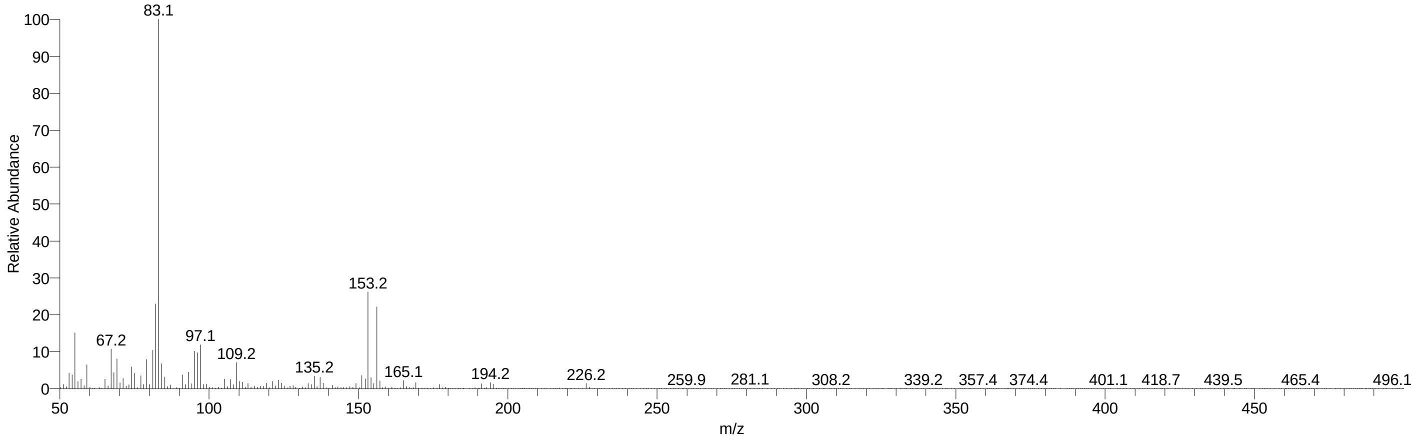

RT	Compound Name	Area %	MF	Molecular Formula	Molecular Weight	Cas #	Library
21.45	METHYL (3-OXO-2-PENTYLCYCLOPENTYL)ACETATE #	2.94	918	C13H22O3	226	24851-98-7	WileyRegistry8e
21.45	METHYL (3-OXO-2-PENTYLCYCLOPENTYL)ACETATE #	2.94	911	C13H22O3	226	24851-98-7	WileyRegistry8e
21.45	Cyclopentaneacetic acid, 3-oxo-2-pentyl-, methyl ester	2.94	848	C13H22O3	226	24851-98-7	mainlib
21.45	METHYL (3-OXO-2-PENTYLCYCLOPENTYL)ACETATE #	2.94	850	C13H22O3	226	24851-98-7	WileyRegistry8e
21.45	5,7-DIMETHYL-6-OCTEN-1-OL	2.94	785	C10H20O	156	18479-52-2	WileyRegistry8e

Compound Structure | Hit Spectrum

METHYL (3-OXO-2-PENTYLCYCLOPENTYL)ACETATE #
Formula C13H22O3, MW 226, CAS# 24851-98-7, Entry# 114153
CYCLOPENTANEACETIC ACID, 3-OXO-2-PENTYL-, METHYL ESTER

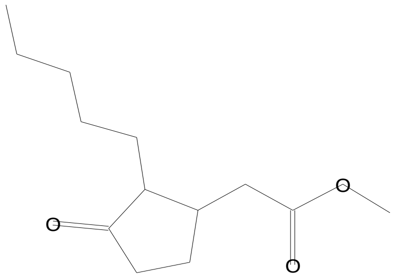

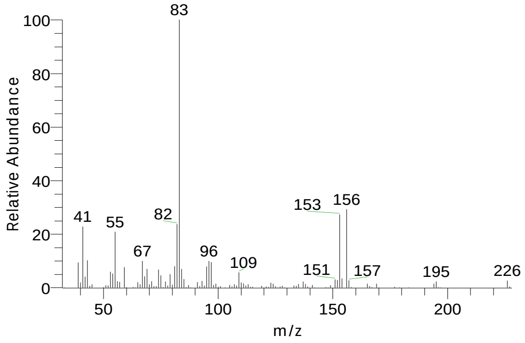

My GC-MS Report

Compound Structure | Hit Spectrum

METHYL (3-OXO-2-PENTYLCYCLOPENTYL)ACETATE #
Formula C13H22O3, MW 226, CAS# 24851-98-7, Entry# 114155
CYCLOPENTANEACETIC ACID, 3-OXO-2-PENTYL-, METHYL ESTER

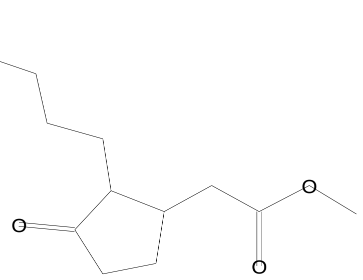

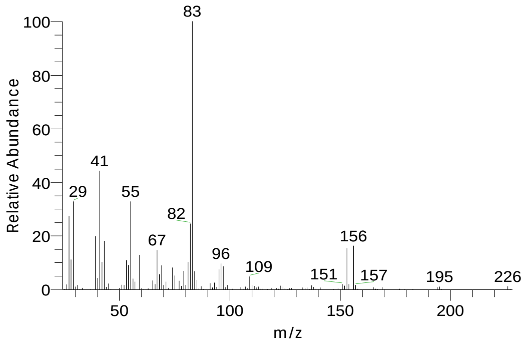

Cyclopentaneacetic acid, 3-oxo-2-pentyl-, methyl ester
Formula C13H22O3, MW 226, CAS# 24851-98-7, Entry# 52825
Methyl (3-oxo-2-pentylcyclopentyl)acetate

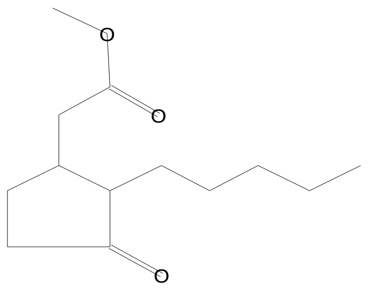

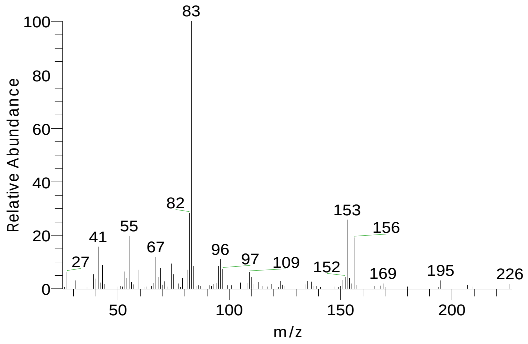

METHYL (3-OXO-2-PENTYLCYCLOPENTYL)ACETATE #
Formula C13H22O3, MW 226, CAS# 24851-98-7, Entry# 114154
CYCLOPENTANEACETIC ACID, 3-OXO-2-PENTYL-, METHYL ESTER

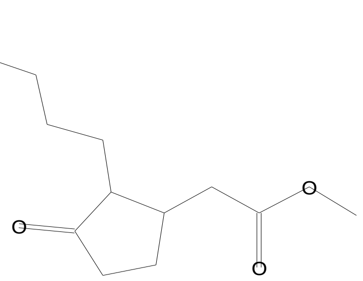

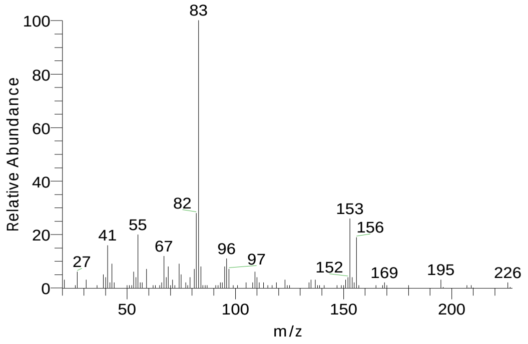

5,7-DIMETHYL-6-OCTEN-1-OL
Formula C10H20O, MW 156, CAS# 18479-52-2, Entry# 40679
ISOCITRONELLOL

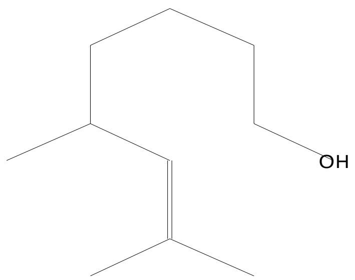

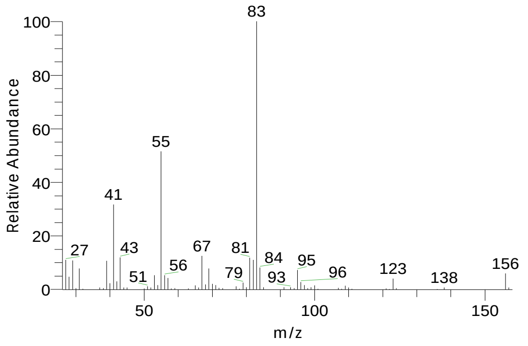

My GC-MS Report

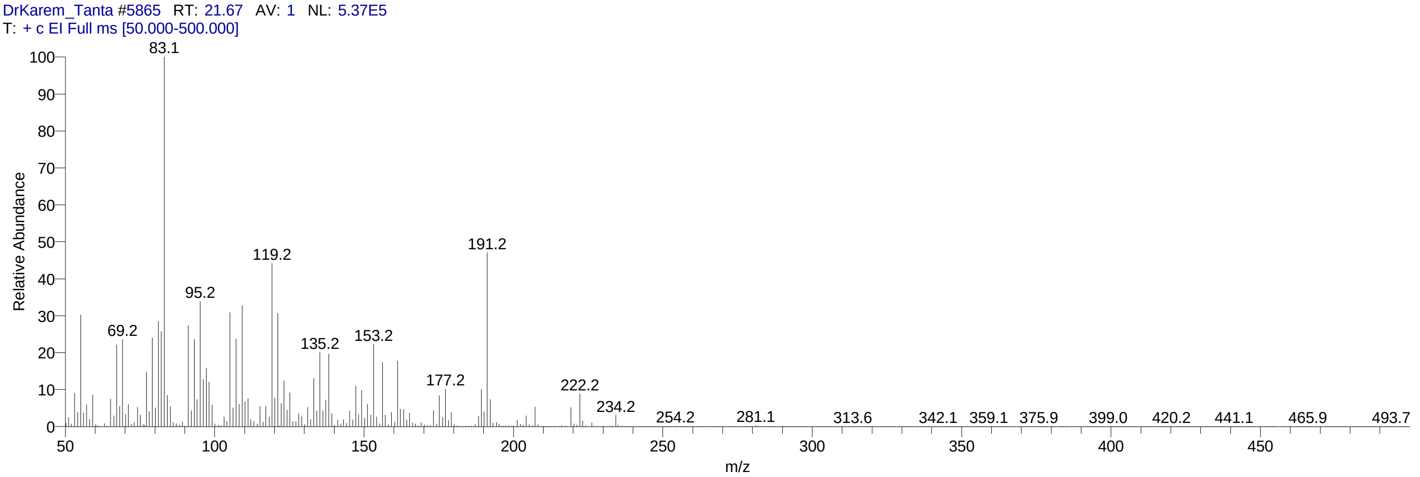

RT	Compound Name	Area %	MF	Molecular Formula	Molecular Weight	Cas #	Library
21.67	1-(4-ISOPROPYLPHENYL)-2-METHYLPROPYL ACETATE	2.81	805	C15H22O2	234	NA	WileyRegistry8e
21.67	1-Naphthalenemethanol, 1,4,4a,5,6,7,8,8a-octahydro-2,5,5,8a-tetramethyl-	2.81	760	C15H26O	222	19078-37-6	mainlib
21.67	(2,5,5,8A-TETRAMETHYL-1,4,4A,5,6,7,8,8A-OCTAHYDRO-1-NAPHTHALENYL)METHANOL	2.81	760	C15H26O	222	19078-37-6	WileyRegistry8e
21.67	1-[3,3-Dimethyl-2-(3-methyl-buta-1,3-dienyl)-cyclopentyl]-2-hydroxy-ethanone	2.81	745	C14H22O2	222	NA	mainlib
21.67	1-[3,3-DIMETHYL-2-(3-METHYL-BUTA-1,3-DIENYL)-CYCLOPENTYL]-2-HYDROXY-ETHANONE	2.81	745	C14H22O2	222	NA	WileyRegistry8e

Compound Structure | Hit Spectrum

1-(4-ISOPROPYLPHENYL)-2-METHYLPROPYL ACETATE
Formula C15H22O2, MW 234, CAS# NA, Entry# 311526
ACETIC ACID, [1-(4-ISOPROPYLPHENYL)-2-METHYL]PROPYL ESTER

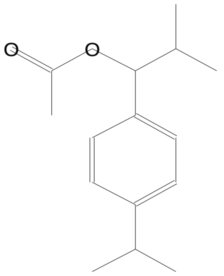

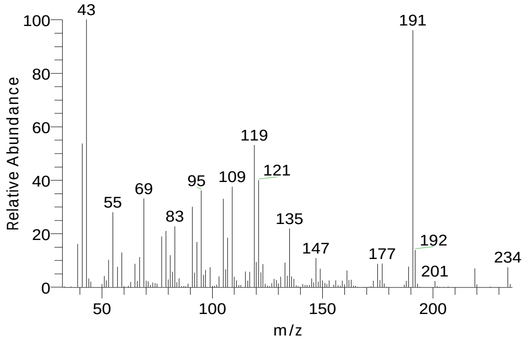

1-Naphthalenemethanol, 1,4,4a,5,6,7,8,8a-octahydro-2,5,5,8a-tetramethyl-
Formula C15H26O, MW 222, CAS# 19078-37-6, Entry# 86293
Drimenol

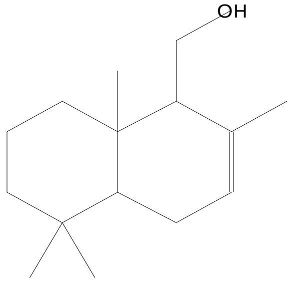

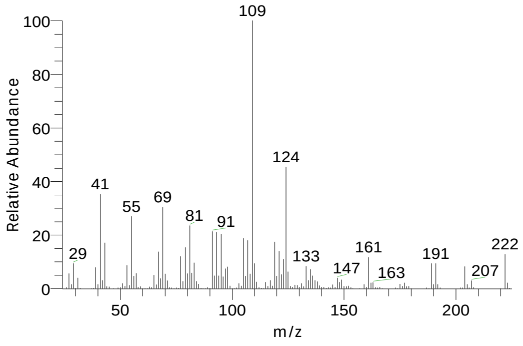

My GC-MS Report

Compound Structure | Hit Spectrum

(2,5,5,8A-TETRAMETHYL-1,4,4A,5,6,7,8,8A-OCTAHYDRO-1-NAPHTHALENYL)METHANOL
Formula C15H26O, MW 222, CAS# 19078-37-6, Entry# 362112
(2,5,5,8A-TETRAMETHYL-1,4,4A,5,6,7,8,8A-OCTAHYDRO-NAPHTHALEN-1-YL)-METHANOL

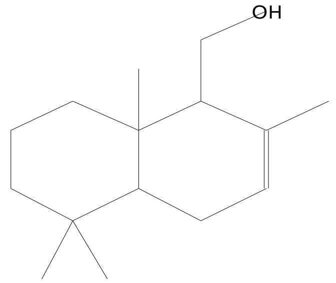

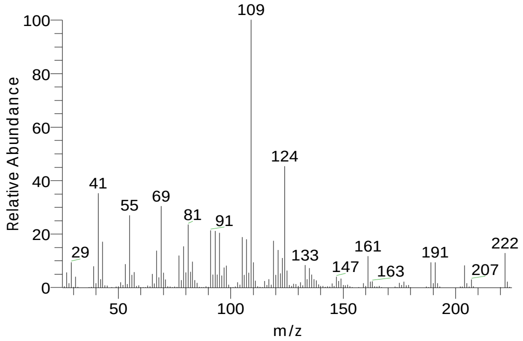

1-[3,3-Dimethyl-2-(3-methyl-buta-1,3-dienyl)-cyclopentyl]-2-hydroxy-ethanone
Formula C14H22O2, MW 222, CAS# NA, Entry# 68396
1-(3,3-Dimethyl-2-[(1E)-3-methyl-1,3-butadienyl]cyclopentyl)-2-hydroxyethanone #

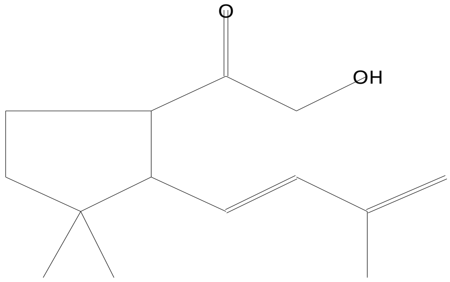

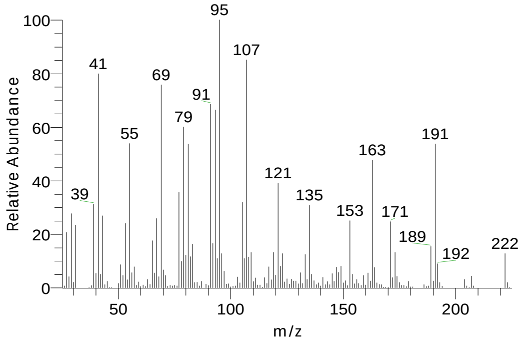

1-[3,3-DIMETHYL-2-(3-METHYL-BUTA-1,3-DIENYL)-CYCLOPENTYL]-2-HYDROXY-ETHANONE
Formula C14H22O2, MW 222, CAS# NA, Entry# 372587
1-{3,3-DIMETHYL-2-[3-METHYL-1,3-BUTADIENYL]CYCLOPENTYL}-2-HYDROXYETHANONE

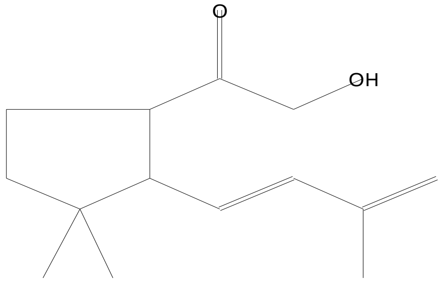

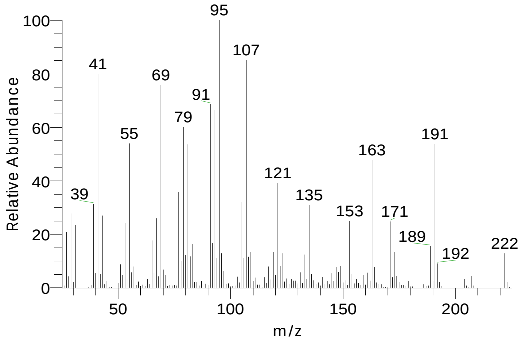

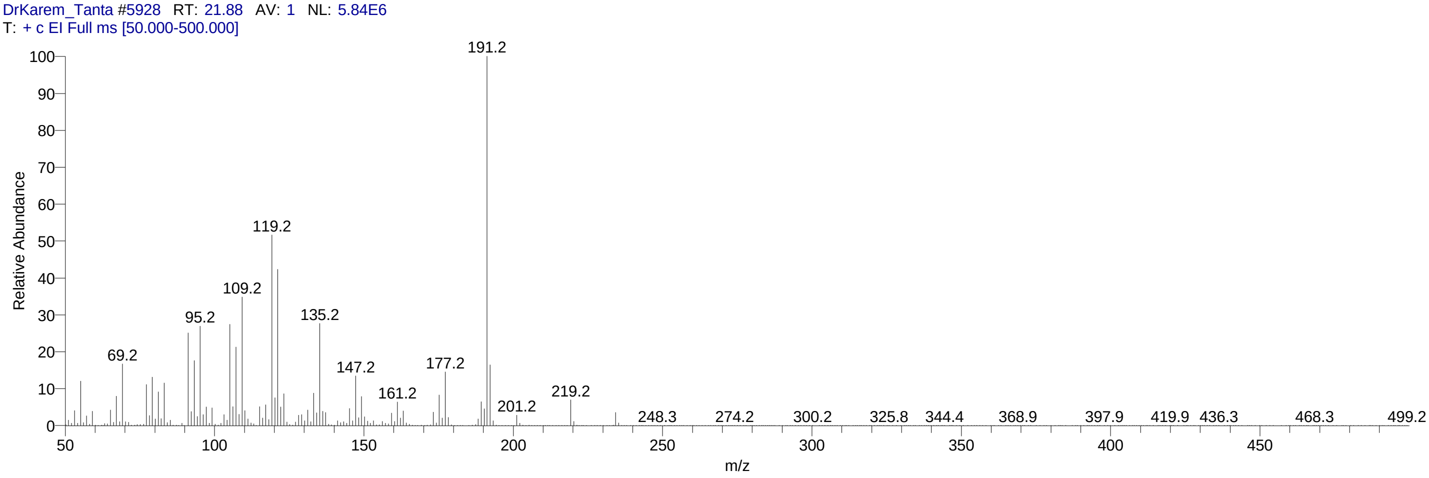

RT	Compound Name	Area %	MF	Molecular Formula	Molecular Weight	Cas #	Library
21.88	1-(4-ISOPROPYLPHENYL)-2-METHYLPROPYL ACETATE	26.87	910	C15H22O2	234	NA	WileyRegistry8e
21.88	(1E)-1-(2,6,6-TRIMETHYL-1-CYCLOHEXEN-1-YL)-1-PENTEN-3-ONE #	26.87	805	C14H22O	206	127-43-5	WileyRegistry8e

My GC-MS Report

RT	Compound Name	Area %	MF	Molecular Formula	Molecular Weight	Cas #	Library
21.88	5á-GUAIA-7(11),10(14)-DIEN-8à-OL, 5,8-EPOXY-, (+)-	26.87	777	C15H22O2	234	24063-71-6	WileyRegistry8e
21.88	(7a-Isopropenyl-4,5-dimethyloctahydroinden-4-yl)methanol	26.87	794	C15H26O	222	NA	mainlib
21.88	(7A-ISOPROPENYL-4,5-DIMETHYL-OCTAHYDRO-INDEN-4-YL)-METHANOL	26.87	794	C15H26O	222	NA	WileyRegistry8e

Compound Structure	Hit Spectrum

1-(4-ISOPROPYLPHENYL)-2-METHYLPROPYL ACETATE
Formula C15H22O2, MW 234, CAS# NA, Entry# 311526
ACETIC ACID, [1-(4-ISOPROPYLPHENYL)-2-METHYL]PROPYL ESTER

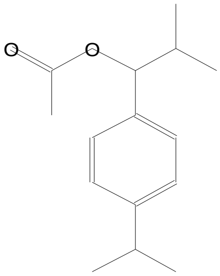

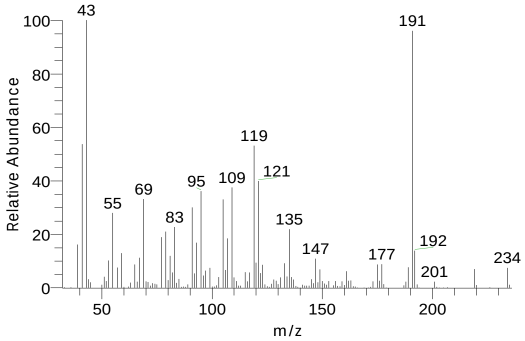

(1E)-1-(2,6,6-TRIMETHYL-1-CYCLOHEXEN-1-YL)-1-PENTEN-3-ONE #
Formula C14H22O, MW 206, CAS# 127-43-5, Entry# 91733
(1E)-1-(2,6,6-TRIMETHYL-1-CYCLOHEXEN-1-YL)-1-PENTEN-3-ONE

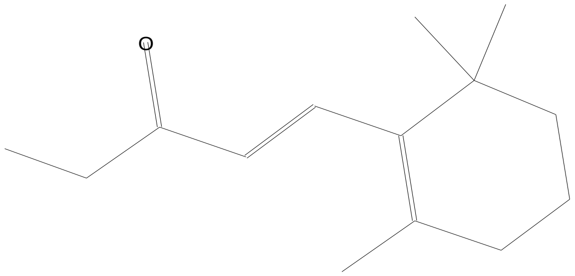

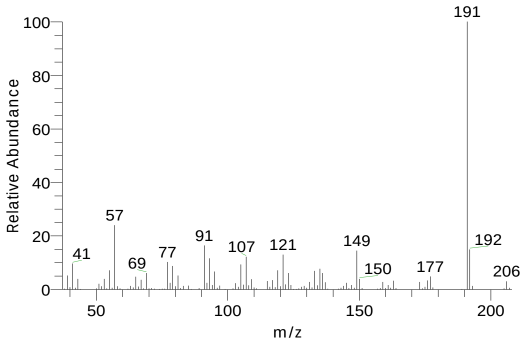

5á-GUAIA-7(11),10(14)-DIEN-8à-OL, 5,8-EPOXY-, (+)-
Formula C15H22O2, MW 234, CAS# 24063-71-6, Entry# 122932

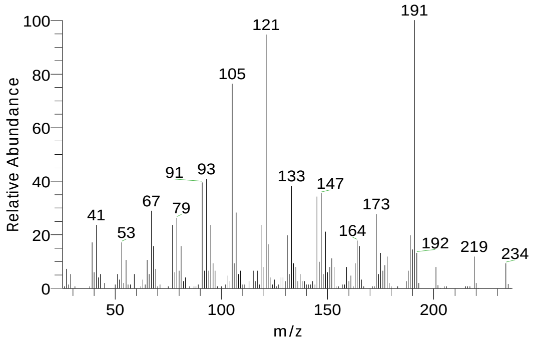

(7a-Isopropenyl-4,5-dimethyloctahydroinden-4-yl)methanol
Formula C15H26O, MW 222, CAS# NA, Entry# 177311
(7a-Isopropenyl-4,5-dimethyloctahydro-1H-inden-4-yl)methanol #

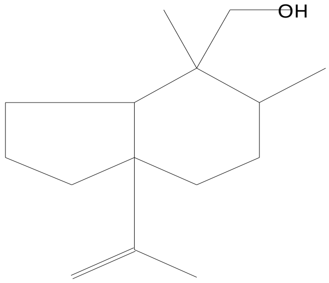

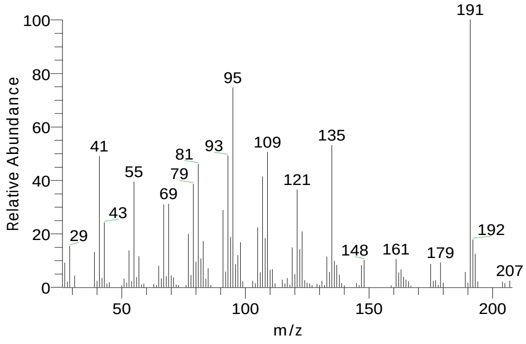

My GC-MS Report

Compound Structure | Hit Spectrum

(7A-ISOPROPENYL-4,5-DIMETHYL-OCTAHYDRO-INDEN-4-YL)-METHANOL
Formula C15H26O, MW 222, CAS# NA, Entry# 374663
(7A-ISOPROPENYL-4,5-DIMETHYLOCTAHYDRO-1H-INDEN-4-YL)METHANOL

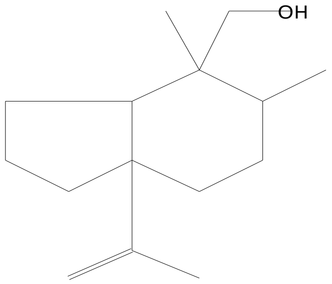

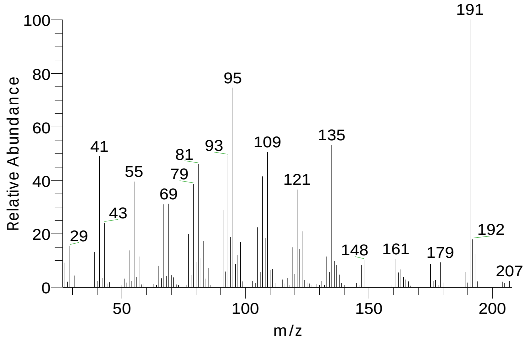

DrKarem_Tanta #5965 RT: 22.00 AV: 1 NL: 1.39E6
T: + c EI Full ms [50.000-500.000]

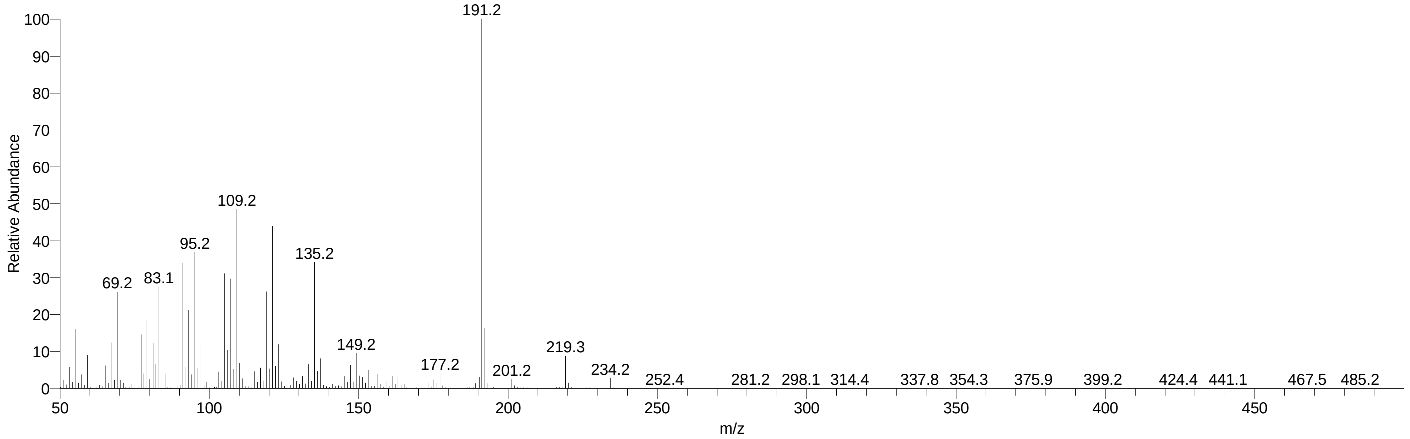

RT	Compound Name	Area %	MF	Molecular Formula	Molecular Weight	Cas #	Library
22.00	1-(4-ISOPROPYLPHENYL)-2-METHYLPROPYL ACETATE	4.92	889	C15H22O2	234	NA	WileyRegistry8e
22.00	(7A-ISOPROPENYL-4,5-DIMETHYL-OCTAHYDRO-INDEN-4-YL)-METHANOL	4.92	814	C15H26O	222	NA	WileyRegistry8e
22.00	(7a-Isopropenyl-4,5-dimethyloctahydroinden-4-yl)methanol	4.92	814	C15H26O	222	NA	mainlib
22.00	(1E)-1-(2,6,6-TRIMETHYL-1-CYCLOHEXEN-1-YL)-1-PENTEN-3-ONE #	4.92	791	C14H22O	206	127-43-5	WileyRegistry8e
22.00	1H-INDENE, 2,3,3A,4,7,7A-HEXAHYDRO-2,2,4,4,7,7-HEXAMETHYL-, TRANS-	4.92	786	C15H26	206	54832-81-4	WileyRegistry8e

Compound Structure | Hit Spectrum

1-(4-ISOPROPYLPHENYL)-2-METHYLPROPYL ACETATE
Formula C15H22O2, MW 234, CAS# NA, Entry# 311526
ACETIC ACID, [1-(4-ISOPROPYLPHENYL)-2-METHYL]PROPYL ESTER

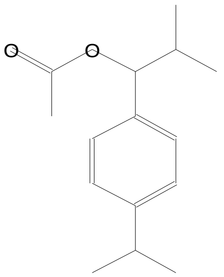

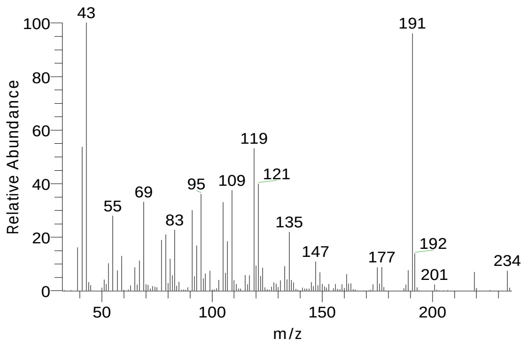

My GC-MS Report

Compound Structure | Hit Spectrum

(7A-ISOPROPENYL-4,5-DIMETHYL-OCTAHYDRO-INDEN-4-YL)-METHANOL
Formula C15H26O, MW 222, CAS# NA, Entry# 374663
(7A-ISOPROPENYL-4,5-DIMETHYLOCTAHYDRO-1H-INDEN-4-YL)METHANOL

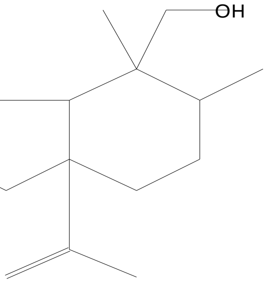

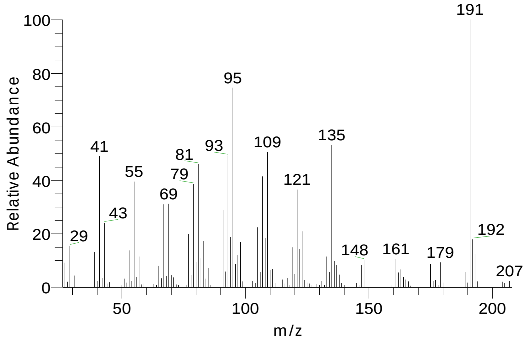

(7a-Isopropenyl-4,5-dimethyloctahydroinden-4-yl)methanol
Formula C15H26O, MW 222, CAS# NA, Entry# 177311
(7a-Isopropenyl-4,5-dimethyloctahydro-1H-inden-4-yl)methanol #

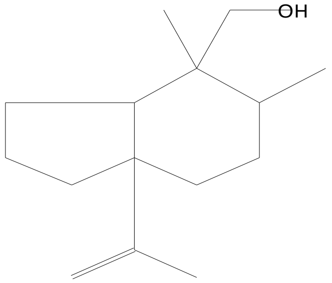

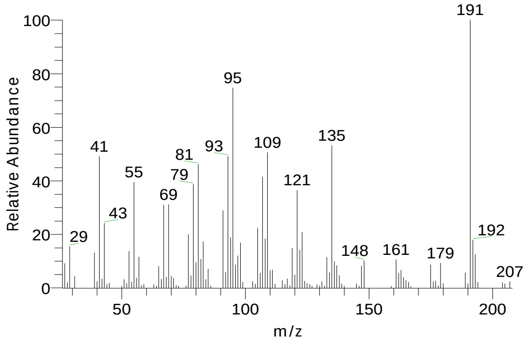

(1E)-1-(2,6,6-TRIMETHYL-1-CYCLOHEXEN-1-YL)-1-PENTEN-3-ONE #
Formula C14H22O, MW 206, CAS# 127-43-5, Entry# 91733
(1E)-1-(2,6,6-TRIMETHYL-1-CYCLOHEXEN-1-YL)-1-PENTEN-3-ONE

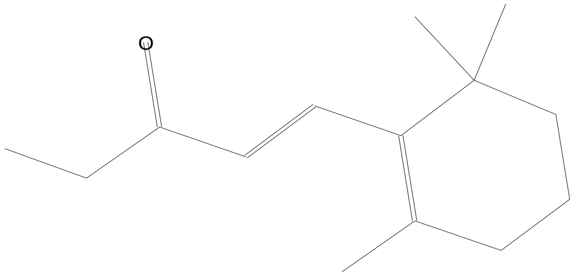

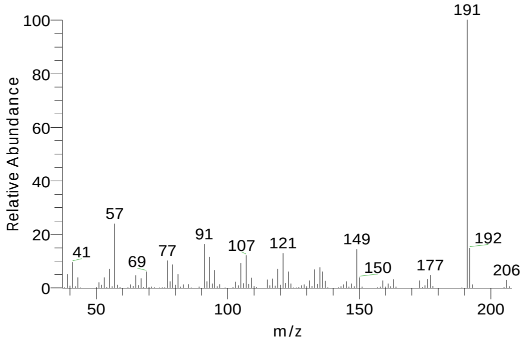

1H-INDENE, 2,3,3A,4,7,7A-HEXAHYDRO-2,2,4,4,7,7-HEXAMETHYL-, TRANS-
Formula C15H26, MW 206, CAS# 54832-81-4, Entry# 91916
2,2,4,4,7,7-HEXAMETHYL-2,3,3A,4,7,7A-HEXAHYDRO-1H-INDENE

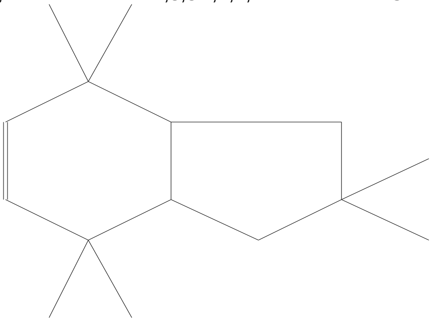

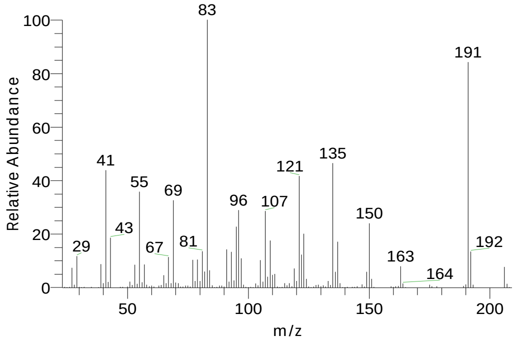

My GC-MS Report

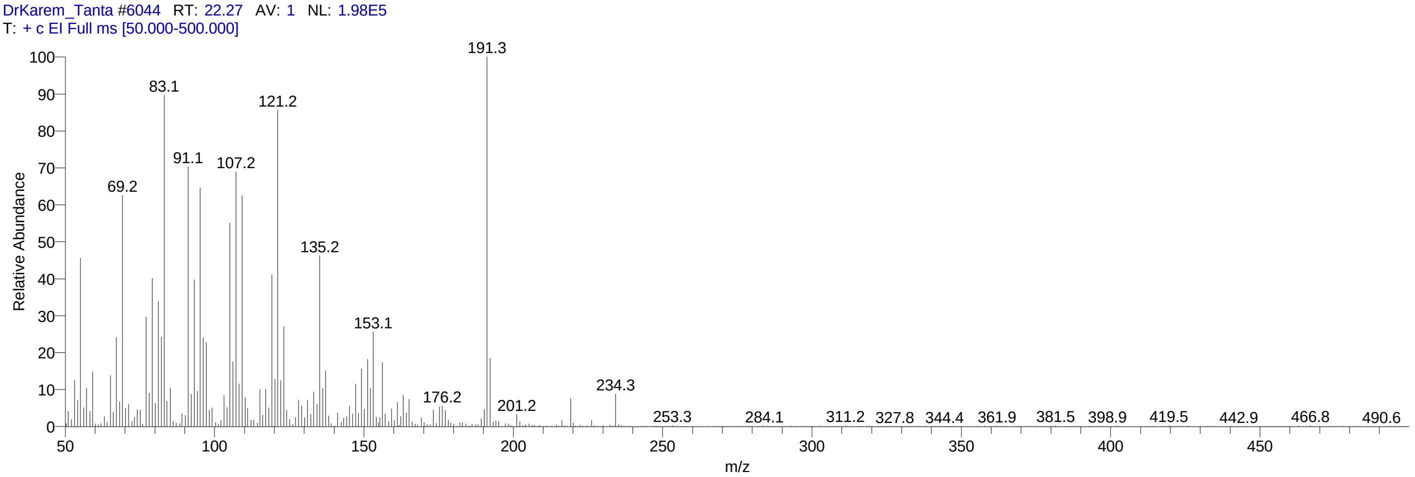

RT	Compound Name	Area %	MF	Molecular Formula	Molecular Weight	Cas #	Library
22.27	1-(4-ISOPROPYLPHENYL)-2-METHYLPROPYL ACETATE	1.33	862	C15H22O2	234	NA	WileyRegistry8e
22.27	2-(4a,8-Dimethyl-6-oxo-1,2,3,4,4a,5,6,8a-octahydro-naphthalen-2-yl)-propionaldehyde	1.33	764	C15H22O2	234	NA	mainlib
22.27	2-(4A,8-DIMETHYL-6-OXO-1,2,3,4,4A,5,6,8A-OCTAHYDRO-2-NAPHTHALENYL)PROPANAL	1.33	764	C15H22O2	234	NA	WileyRegistry8e
22.27	1-[3,3-DIMETHYL-2-(3-METHYL-BUTA-1,3-DIENYL)-CYCLOPENTYL]-2-HYDROXY-ETHANONE	1.33	764	C14H22O2	222	NA	WileyRegistry8e
22.27	1-[3,3-Dimethyl-2-(3-methyl-buta-1,3-dienyl)-cyclopentyl]-2-hydroxy-ethanone	1.33	763	C14H22O2	222	NA	mainlib

Compound Structure | Hit Spectrum

1-(4-ISOPROPYLPHENYL)-2-METHYLPROPYL ACETATE
Formula C15H22O2, MW 234, CAS# NA, Entry# 311526
ACETIC ACID, [1-(4-ISOPROPYLPHENYL)-2-METHYL]PROPYL ESTER

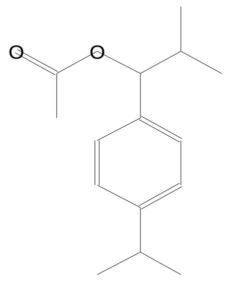

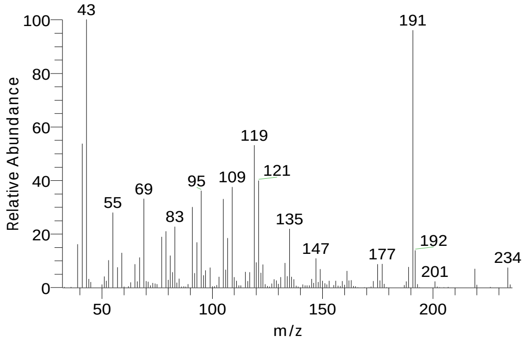

2-(4a,8-Dimethyl-6-oxo-1,2,3,4,4a,5,6,8a-octahydro-naphthalen-2-yl)-propionaldehyde
Formula C15H22O2, MW 234, CAS# NA, Entry# 67765
2-(4a,8-Dimethyl-6-oxo-1,2,3,4,4a,5,6,8a-octahydro-2-naphthalenyl)propanal #

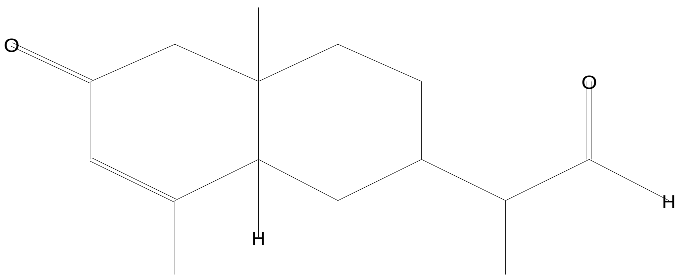

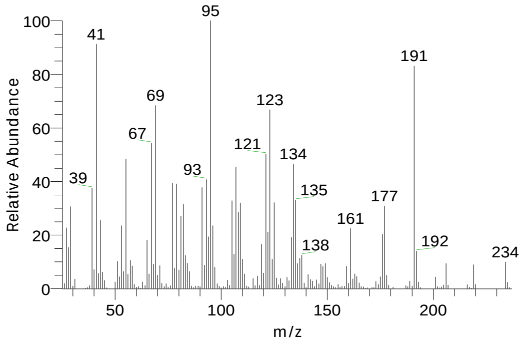

My GC-MS Report

Compound Structure | Hit Spectrum

2-(4A,8-DIMETHYL-6-OXO-1,2,3,4,4A,5,6,8A-OCTAHYDRO-2-NAPHTHALENYL)PROPANAL
Formula C15H22O2, MW 234, CAS# NA, Entry# 371504

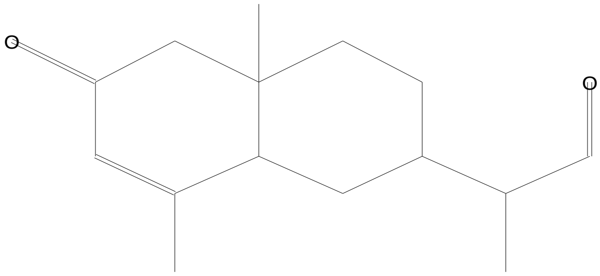

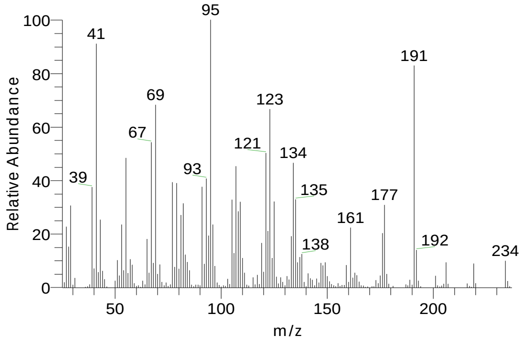

1-[3,3-DIMETHYL-2-(3-METHYL-BUTA-1,3-DIENYL)-CYCLOPENTYL]-2-HYDROXY-ETHANONE
Formula C14H22O2, MW 222, CAS# NA, Entry# 372587
1-{3,3-DIMETHYL-2-[3-METHYL-1,3-BUTADIENYL]CYCLOPENTYL}-2-HYDROXYETHANONE

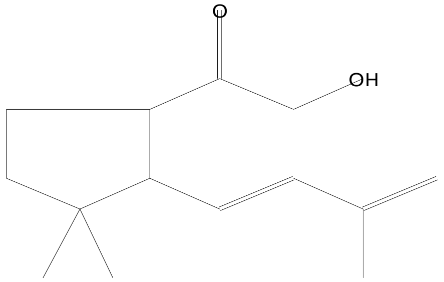

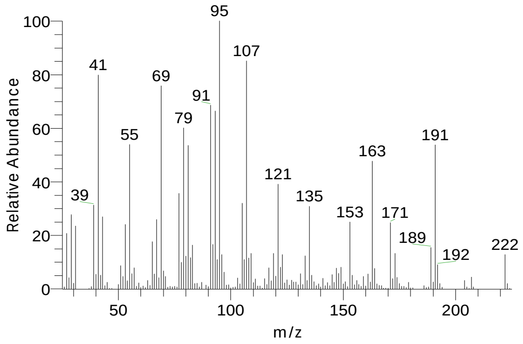

1-[3,3-Dimethyl-2-(3-methyl-buta-1,3-dienyl)-cyclopentyl]-2-hydroxy-ethanone
Formula C14H22O2, MW 222, CAS# NA, Entry# 68396
1-(3,3-Dimethyl-2-[(1E)-3-methyl-1,3-butadienyl]cyclopentyl)-2-hydroxyethanone #

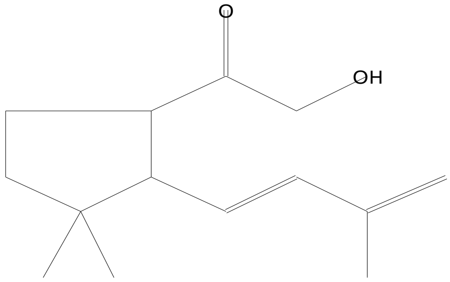

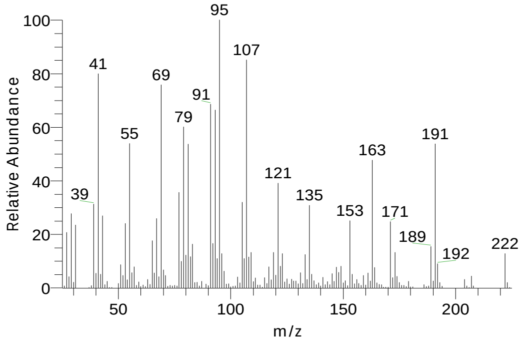

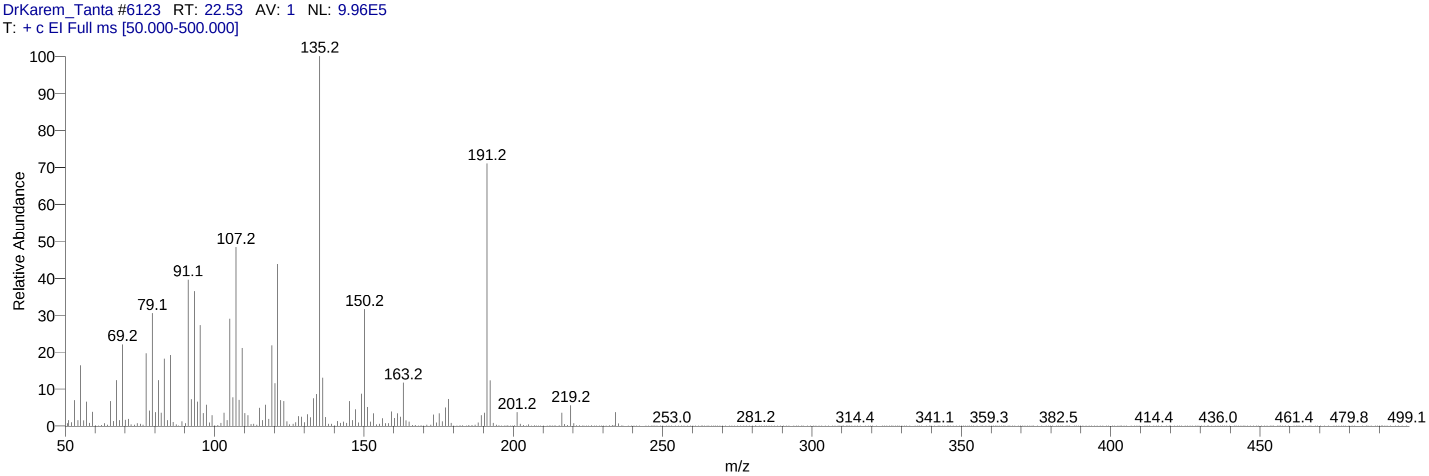

RT	Compound Name	Area %	MF	Molecular Formula	Molecular Weight	Cas #	Library
22.53	1-(4-ISOPROPYLPHENYL)-2-METHYLPROPYL ACETATE	7.43	834	C15H22O2	234	NA	WileyRegistry8e
22.53	3-METHYL-4-(2,6,6-TRIMETHYL-2-CYCLOHEXEN-1-YL)-3-BUTEN-2-ONE	7.43	827	C14H22O	206	127-51-5	WileyRegistry8e

My GC-MS Report

RT	Compound Name	Area %	MF	Molecular Formula	Molecular Weight	Cas #	Library
22.53	3-METHYL-4-(2,6,6-TRIMETHYL-1-CYCLOHEXEN-1-YL)-3-BUTEN-2-ONE	7.43	793	C14H22O	206	79-89-0	WileyRegistry8e
22.53	3-METHYL-4-(2,6,6-TRIMETHYL-1-CYCLOHEXEN-1-YL)-3-BUTEN-2-ONE	7.43	793	C14H22O	206	79-89-0	WileyRegistry8e
22.53	1H-Indene, 2,3,3a,4,7,7a-hexahydro-2,2,4,4,7,7-hexamethyl-, trans-	7.43	781	C15H26	206	54832-81-4	mainlib

Compound Structure	Hit Spectrum

1-(4-ISOPROPYLPHENYL)-2-METHYLPROPYL ACETATE
Formula C15H22O2, MW 234, CAS# NA, Entry# 311526
ACETIC ACID, [1-(4-ISOPROPYLPHENYL)-2-METHYL]PROPYL ESTER

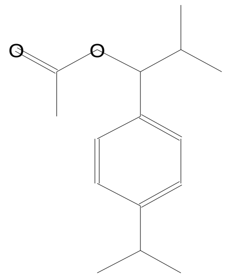

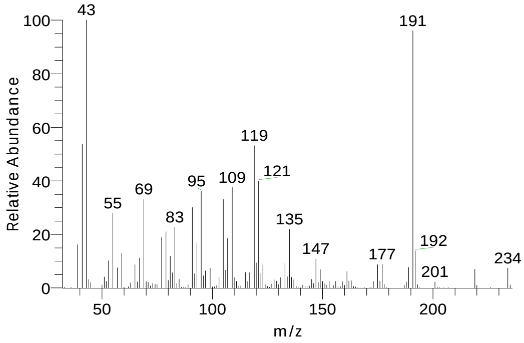

3-METHYL-4-(2,6,6-TRIMETHYL-2-CYCLOHEXEN-1-YL)-3-BUTEN-2-ONE
Formula C14H22O, MW 206, CAS# 127-51-5, Entry# 365083

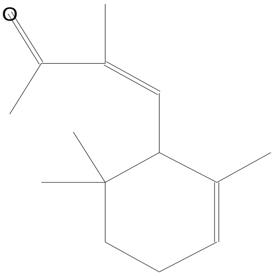

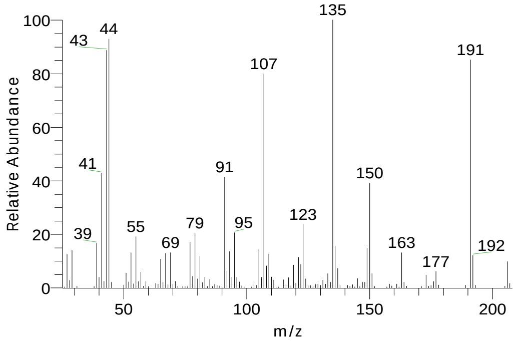

3-METHYL-4-(2,6,6-TRIMETHYL-1-CYCLOHEXEN-1-YL)-3-BUTEN-2-ONE
Formula C14H22O, MW 206, CAS# 79-89-0, Entry# 364092

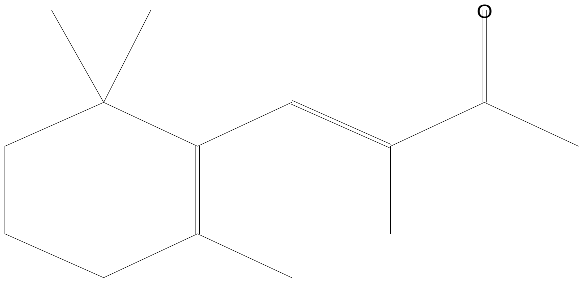

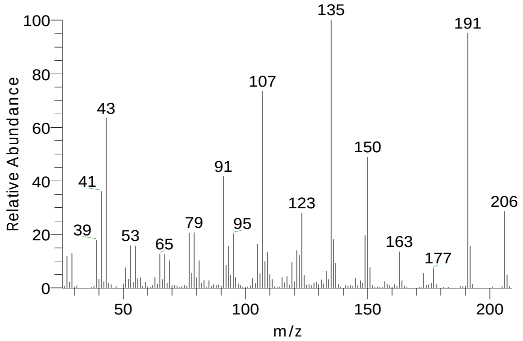

3-METHYL-4-(2,6,6-TRIMETHYL-1-CYCLOHEXEN-1-YL)-3-BUTEN-2-ONE
Formula C14H22O, MW 206, CAS# 79-89-0, Entry# 366469
3-METHYL-4-(2,6,6-TRIMETHYL-CYCLOHEX-1-ENYL)-BUT-3-EN-2-ONE

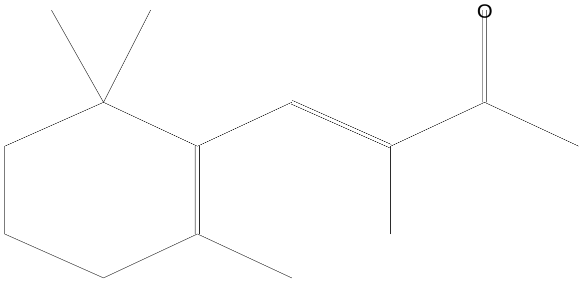

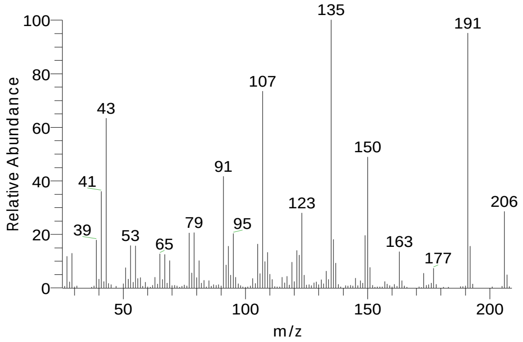

My GC-MS Report

Compound Structure | Hit Spectrum

1H-Indene, 2,3,3a,4,7,7a-hexahydro-2,2,4,4,7,7-hexamethyl-, trans-
Formula C15H26, MW 206, CAS# 54832-81-4, Entry# 53333
2,2,4,4,7,7-Hexamethyl-2,3,3a,4,7,7a-hexahydro-1H-indene #

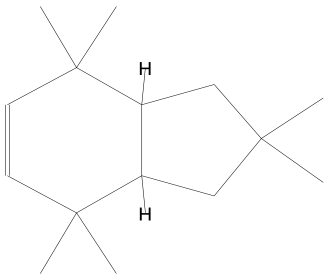

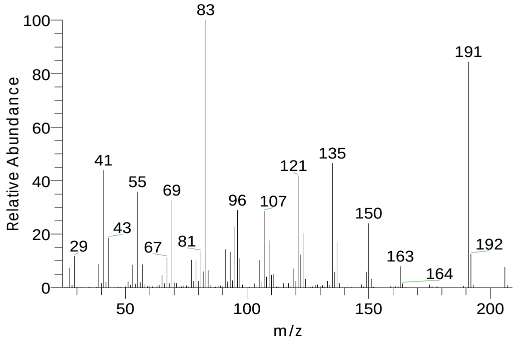

DrKarem_Tanta #6502 RT: 23.80 AV: 1 NL: 3.29E5
T: + c EI Full ms [50.000-500.000]

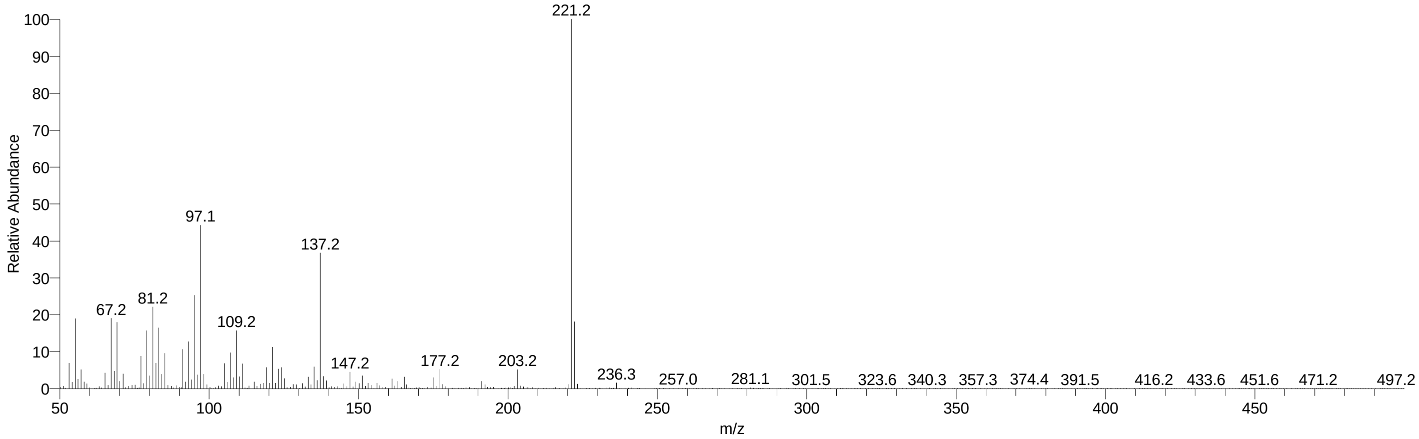

RT	Compound Name	Area %	MF	Molecular Formula	Molecular Weight	Cas #	Library
23.80	Naphtho[2,1-b]furan, dodecahydro-3a,6,6,9a-tetramethyl-	1.19	914	C16H28O	236	3738-00-9	mainlib
23.80	Ambrox	1.19	862	C16H28O	236	100679-85-4	mainlib
23.80	AMBROX	1.19	861	C16H28O	236	65588-69-4	WileyRegistry8e
23.80	(4AR,9AS,9BR)-4A,6,6,9A-TETRAMETHYL-TRANS-PERHYDROINDANO[2,1-C]PYRAN	1.19	759	C16H22O	230	NA	WileyRegistry8e
23.80	NAPHTHO[2,1-B]FURAN, DODECAHYDRO-3A,6,6,9A-TETRAMETHYL-, (3Aà,5Aá,9Aà,9Bá)-(.+-.)-	1.19	972	C16H28O	236	100679-85-4	WileyRegistry8e

Compound Structure | Hit Spectrum

Naphtho[2,1-b]furan, dodecahydro-3a,6,6,9a-tetramethyl-
Formula C16H28O, MW 236, CAS# 3738-00-9, Entry# 196473
Dodecahydro-3a,6,6,9a-tetramethylnaphtho[2,1-á]furan

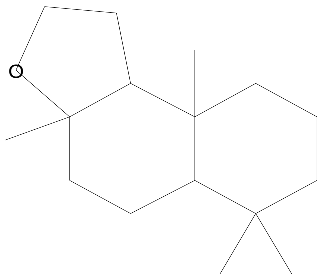

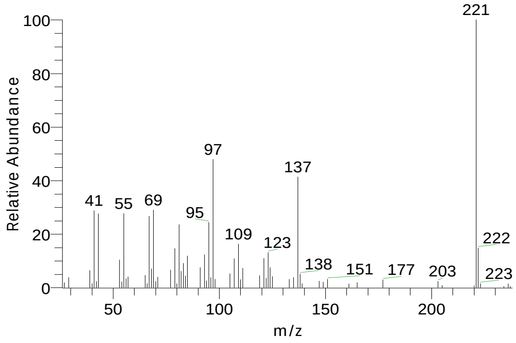

My GC-MS Report

Compound Structure | Hit Spectrum

Ambrox
Formula C16H28O, MW 236, CAS# 100679-85-4, Entry# 196474
3a,6,6,9a-Tetramethyldodecahydronaphtho[2,1-b]furan #

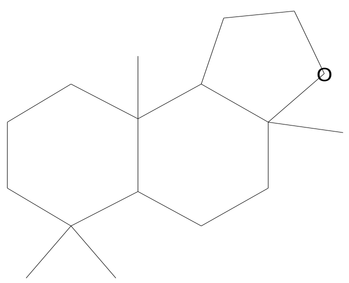

SI 836, RSI 862, mainlib, Entry# 196474, CAS# 100679-85-4, Ambrox

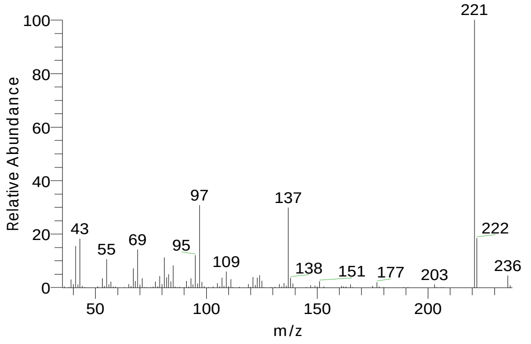

AMBROX
Formula C16H28O, MW 236, CAS# 65588-69-4, Entry# 125644

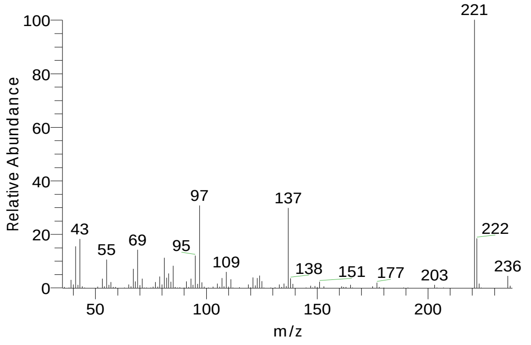

(4AR,9AS,9BR)-4A,6,6,9A-TETRAMETHYL-TRANS-PERHYDROINDANO[2,1-C]PYRAN
Formula C16H22O, MW 230, CAS# NA, Entry# 118906

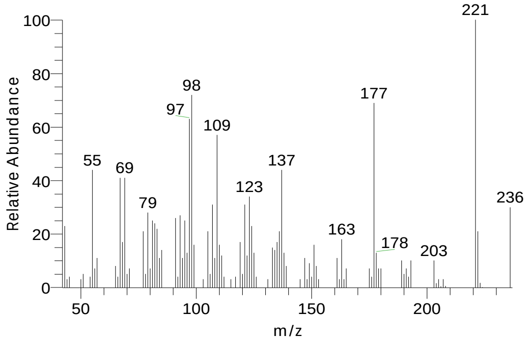

NAPHTHO[2,1-B]FURAN, DODECAHYDRO-3A,6,6,9A-TETRAMETHYL-, (3Aà,5Aá,9Aà,9Bá)-(.+-.)-
Formula C16H28O, MW 236, CAS# 100679-85-4, Entry# 125614
3A,6,6,9A-TETRAMETHYLDODECAHYDRONAPHTHO[2,1-B]FURAN #

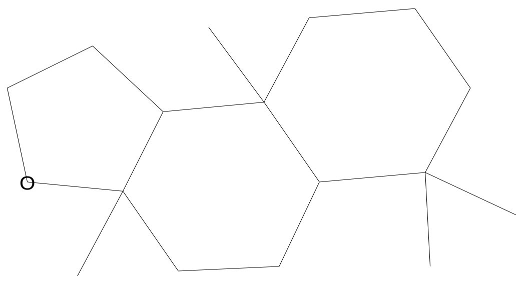

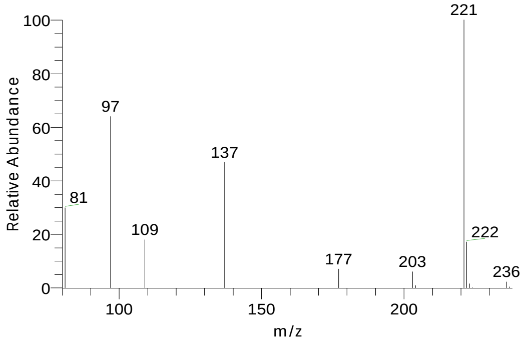

My GC-MS Report

DrKarem_Tanta #6849 RT: 24.97 AV: 1 NL: 4.64E5
T: + c EI Full ms [50.000-500.000]

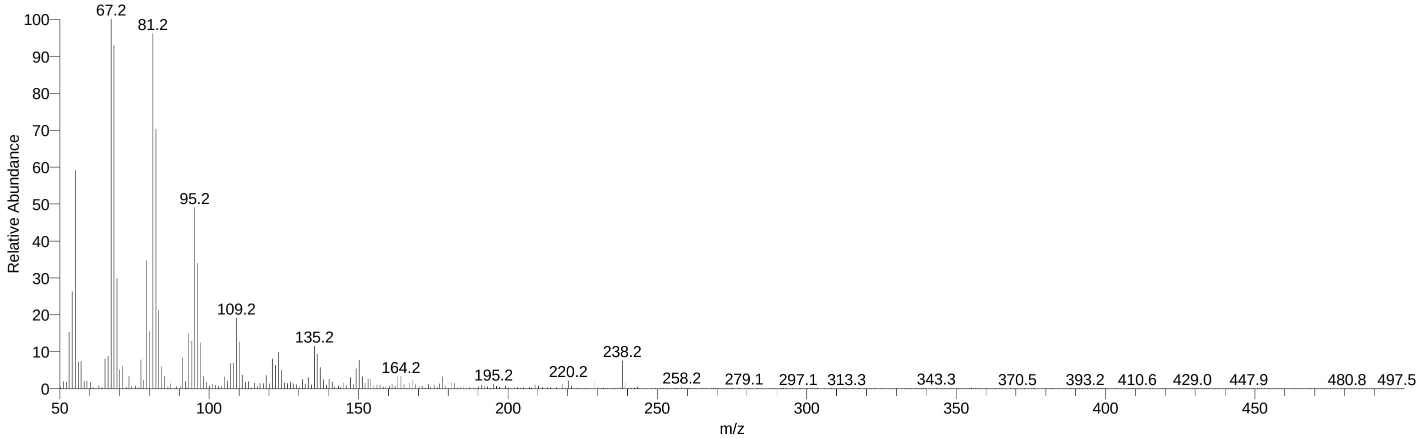

RT	Compound Name	Area %	MF	Molecular Formula	Molecular Weight	Cas #	Library
24.97	9,12-Octadecadienoic acid (Z,Z)-	2.92	871	C18H32O2	280	60-33-3	replib
24.97	Oxacycloheptadec-8-en-2-one, (8Z)-	2.92	854	C16H28O2	252	123-69-3	mainlib
24.97	9,12-Octadecadienoic acid (Z,Z)-	2.92	838	C18H32O2	280	60-33-3	replib
24.97	ETHYL (9Z,12Z)-9,12-OCTADECADIENOATE #	2.92	858	C20H36O2	308	544-35-4	WileyRegistry8e
24.97	Linoelaidic acid	2.92	897	C18H32O2	280	506-21-8	mainlib

Compound Structure | Hit Spectrum

9,12-Octadecadienoic acid (Z,Z)-
Formula C18H32O2, MW 280, CAS# 60-33-3, Entry# 8129
cis-9,cis-12-Octadecadienoic acid

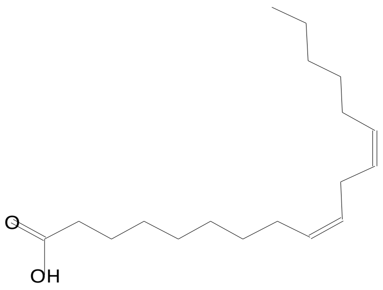

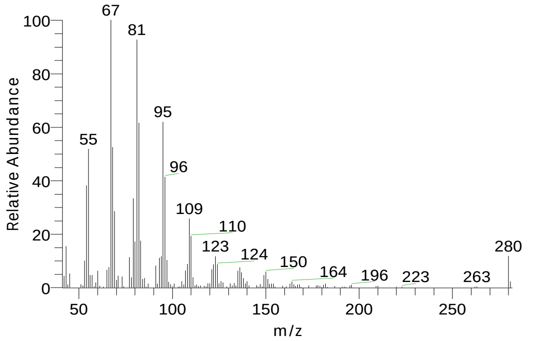

Oxacycloheptadec-8-en-2-one, (8Z)-
Formula C16H28O2, MW 252, CAS# 123-69-3, Entry# 32965
Musk ambrette

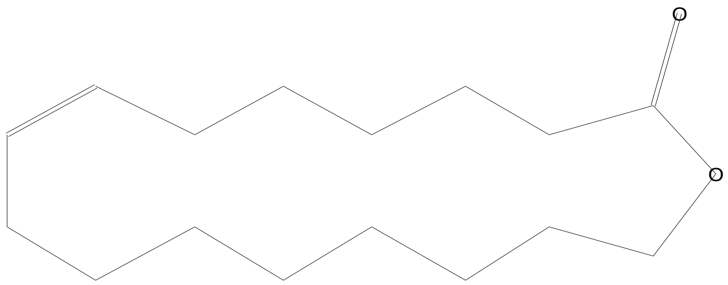

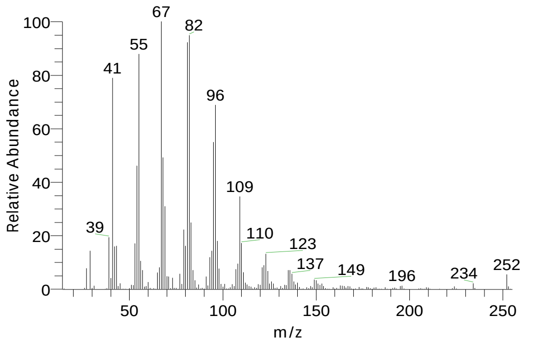

My GC-MS Report

Compound Structure | Hit Spectrum

9,12-Octadecadienoic acid (Z,Z)-
Formula $C_{18}H_{32}O_2$, MW 280, CAS# 60-33-3, Entry# 8112
cis-9,cis-12-Octadecadienoic acid

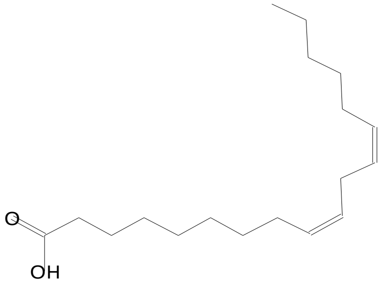

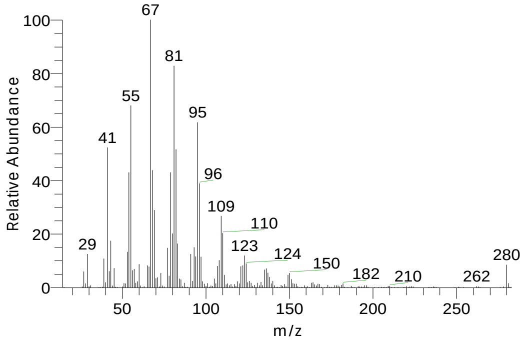

ETHYL (9Z,12Z)-9,12-OCTADECADIENOATE #
Formula $C_{20}H_{36}O_2$, MW 308, CAS# 544-35-4, Entry# 196852
9,12-OCTADECADIENOIC ACID (9Z,12Z)-, ETHYL ESTER

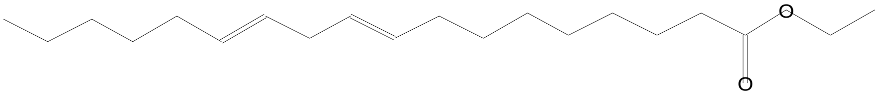

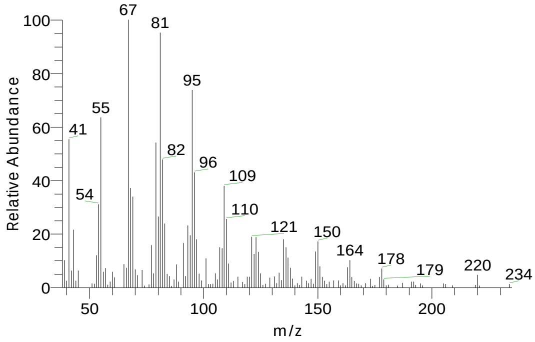

Linoelaidic acid
Formula $C_{18}H_{32}O_2$, MW 280, CAS# 506-21-8, Entry# 32814
$:28OYHQOLUKZRVURQ-AVQMFFATSA-N

SI 821, RSI 897, mainlib, Entry# 32814, CAS# 506-21-8, Linoelaidic acid

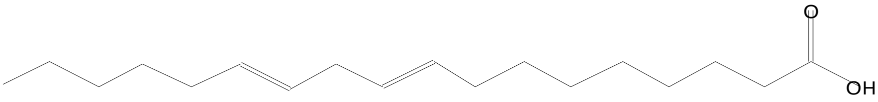

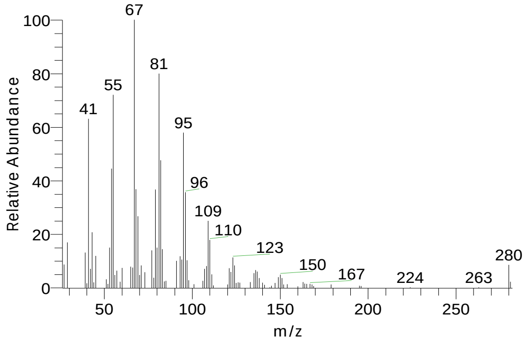

DrKarem_Tanta #6884 RT: 25.08 AV: 1 NL: 3.38E5
T: + c EI Full ms [50.000-500.000]

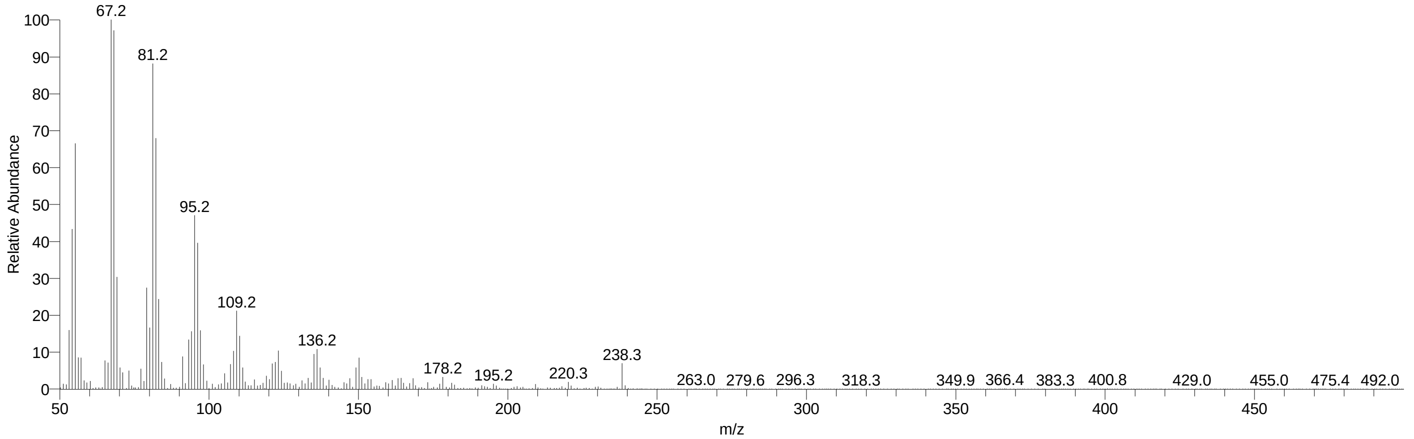

RT	Compound Name	Area %	MF	Molecular Formula	Molecular Weight	Cas #	Library
25.08	9,12-Octadecadienoic acid (Z,Z)-	2.07	874	C18H32O2	280	60-33-3	replib
25.08	Oxacycloheptadec-8-en-2-one, (8Z)-	2.07	856	C16H28O2	252	123-69-3	mainlib
25.08	9,12-Octadecadienoic acid (Z,Z)-	2.07	835	C18H32O2	280	60-33-3	replib

My GC-MS Report

RT	Compound Name	Area %	MF	Molecular Formula	Molecular Weight	Cas #	Library
25.08	ETHYL (9Z,12Z)-9,12-OCTADECADIENOATE #	2.07	850	C20H36O2	308	544-35-4	WileyRegistry8e
25.08	OXACYCLOHEPTADEC-8-EN-2-ONE	2.07	860	C16H28O2	252	123-69-3	WileyRegistry8e

Compound Structure | Hit Spectrum

9,12-Octadecadienoic acid (Z,Z)-
Formula C18H32O2, MW 280, CAS# 60-33-3, Entry# 8129
cis-9,cis-12-Octadecadienoic acid

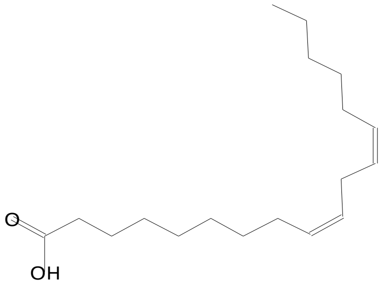

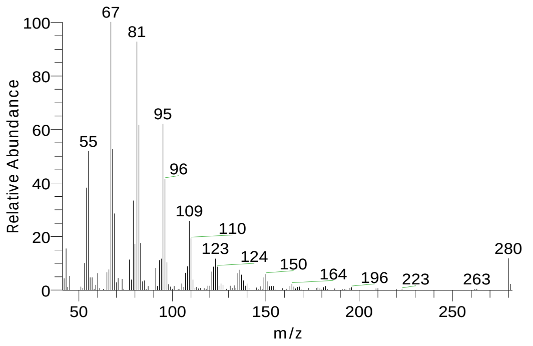

Oxacycloheptadec-8-en-2-one, (8Z)-
Formula C16H28O2, MW 252, CAS# 123-69-3, Entry# 32965
Musk ambrette

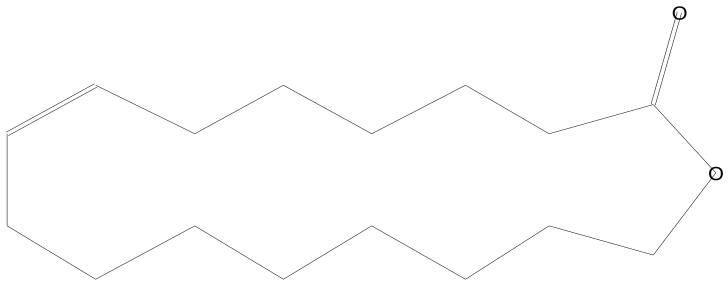

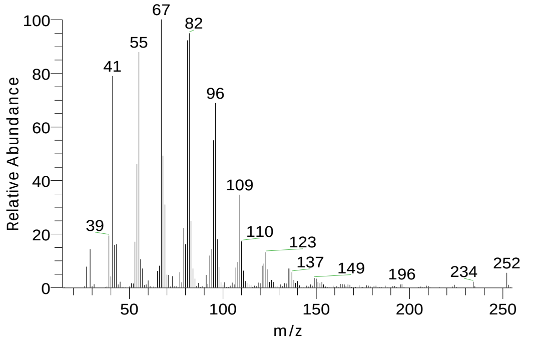

9,12-Octadecadienoic acid (Z,Z)-
Formula C18H32O2, MW 280, CAS# 60-33-3, Entry# 8112
cis-9,cis-12-Octadecadienoic acid

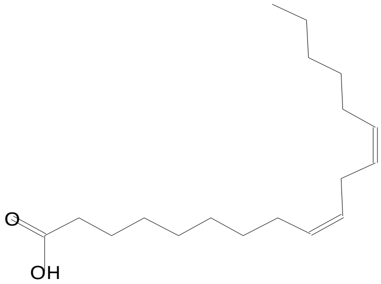

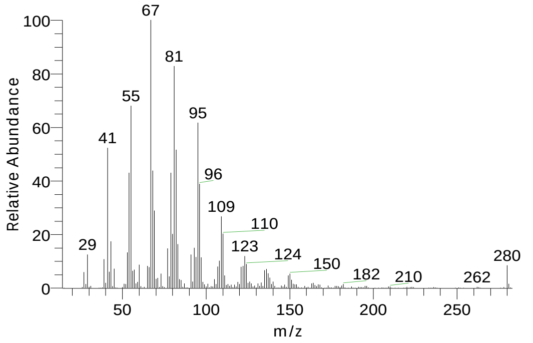

ETHYL (9Z,12Z)-9,12-OCTADECADIENOATE #
Formula C20H36O2, MW 308, CAS# 544-35-4, Entry# 196852
9,12-OCTADECADIENOIC ACID (9Z,12Z)-, ETHYL ESTER

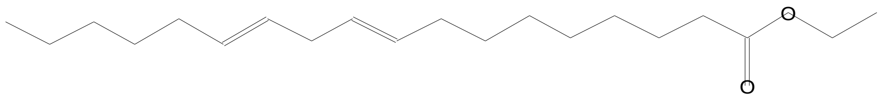

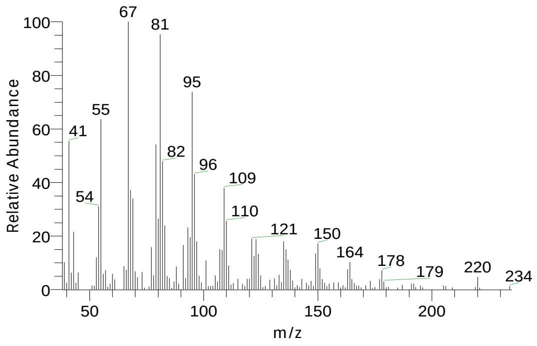

My GC-MS Report

Compound Structure | Hit Spectrum

OXACYCLOHEPTADEC-8-EN-2-ONE
Formula C16H28O2, MW 252, CAS# 123-69-3, Entry# 142708
16-HYDROXY-7-HEXADECENOIC ACID LACTONE

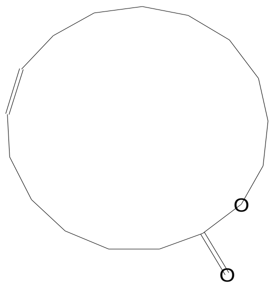

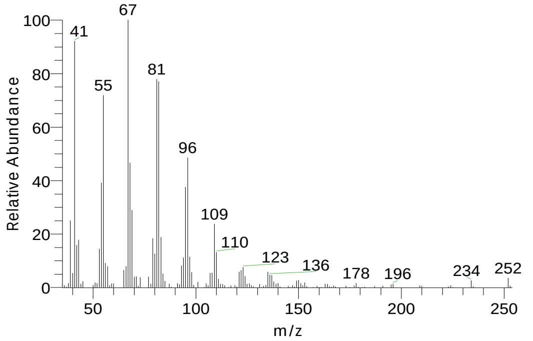

DrKarem_Tanta #6943 RT: 25.28 AV: 1 NL: 1.05E6
T: + c EI Full ms [50.000-500.000]

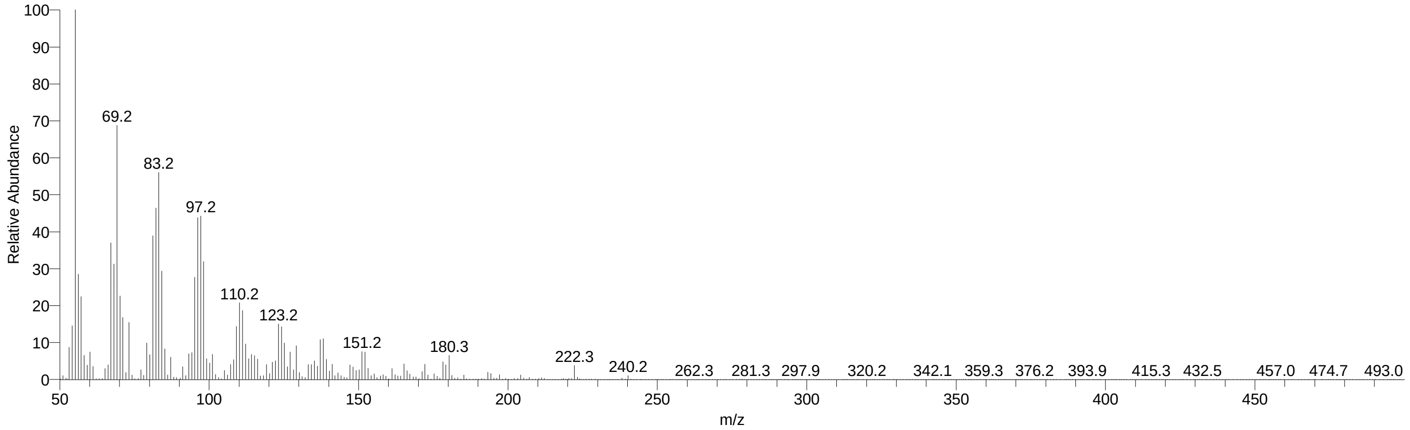

RT	Compound Name	Area %	MF	Molecular Formula	Molecular Weight	Cas #	Library
25.28	Cyclopentadecanone, 2-hydroxy-	8.08	926	C15H28O2	240	4727-18-8	mainlib
25.28	2-HYDROXYCYCLOPENTADECANONE #	8.08	926	C15H28O2	240	4727-18-8	WileyRegistry8e
25.28	Cyclopentadecanone, 2-hydroxy-	8.08	934	C15H28O2	240	4727-18-8	replib
25.28	Oxacyclotetradecane-2,11-dione, 13-methyl-	8.08	916	C14H24O3	240	74685-36-2	mainlib
25.28	OXACYCLOTETRADECANE-2,11-DIONE, 13-METHYL-	8.08	916	C14H24O3	240	74685-36-2	WileyRegistry8e

Compound Structure | Hit Spectrum

Cyclopentadecanone, 2-hydroxy-
Formula C15H28O2, MW 240, CAS# 4727-18-8, Entry# 19282
2-Hydroxycyclopentadecanone #

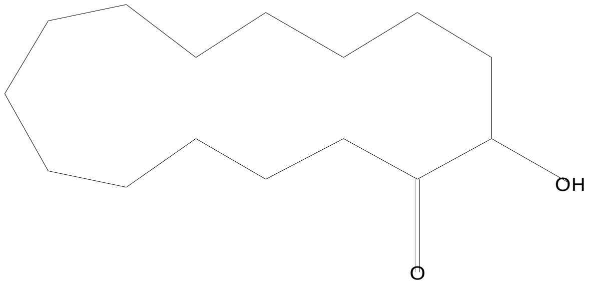

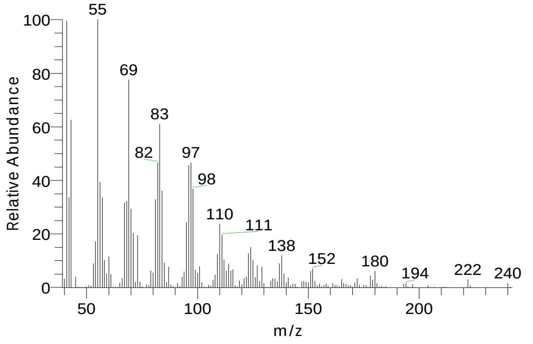

My GC-MS Report

Compound Structure | Hit Spectrum

2-HYDROXYCYCLOPENTADECANONE #
Formula C15H28O2, MW 240, CAS# 4727-18-8, Entry# 129954
2-HYDROXYCYCLOPENTADECANONE

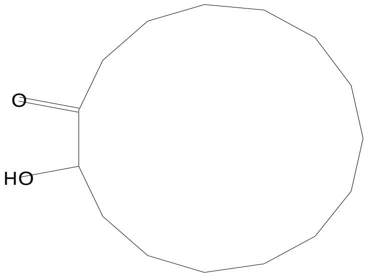

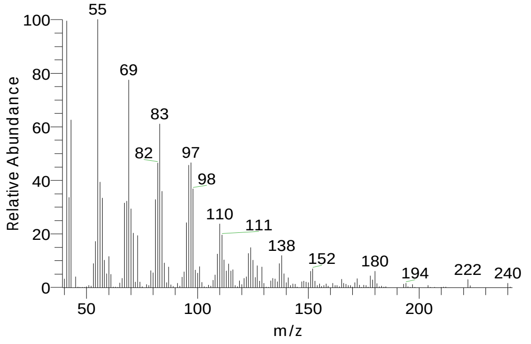

Cyclopentadecanone, 2-hydroxy-
Formula C15H28O2, MW 240, CAS# 4727-18-8, Entry# 4767
2-Hydroxycyclopentadecanone #

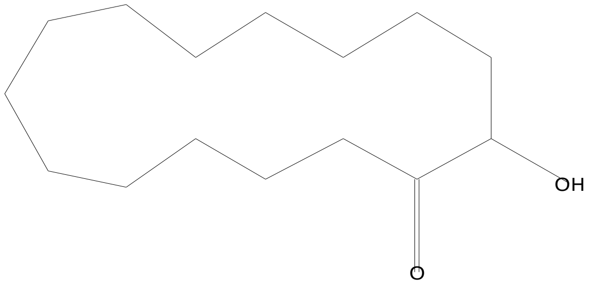

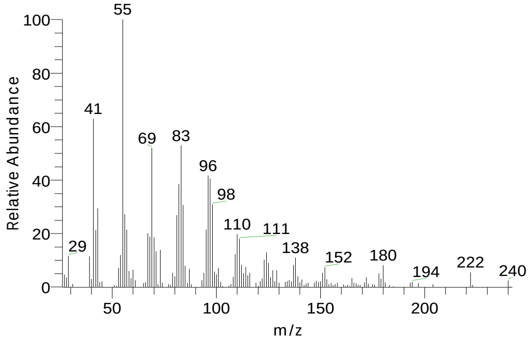

Oxacyclotetradecane-2,11-dione, 13-methyl-
Formula C14H24O3, MW 240, CAS# 74685-36-2, Entry# 19298
13-Methyloxacyclotetradecane-2,11-dione #

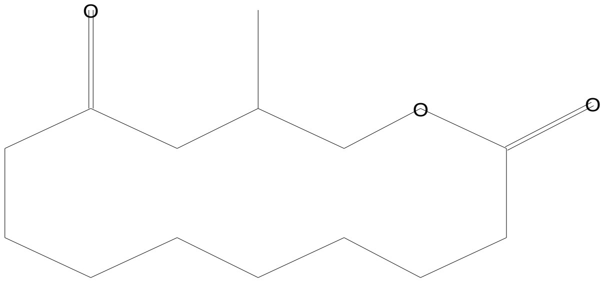

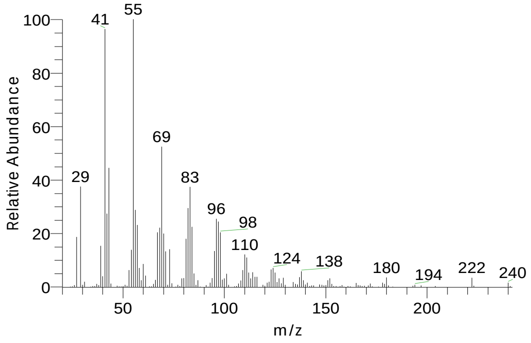

OXACYCLOTETRADECANE-2,11-DIONE, 13-METHYL-
Formula C14H24O3, MW 240, CAS# 74685-36-2, Entry# 129676
13-METHYLOXACYCLOTETRADECANE-2,11-DIONE #

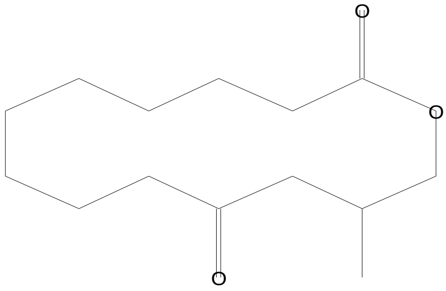

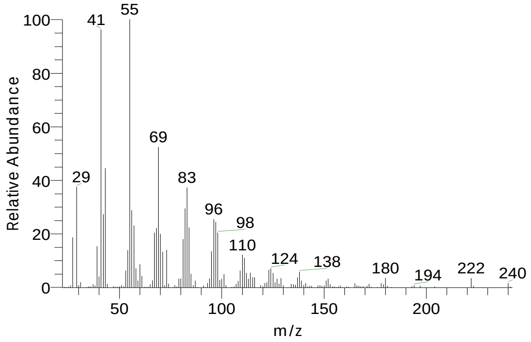

My GC-MS Report

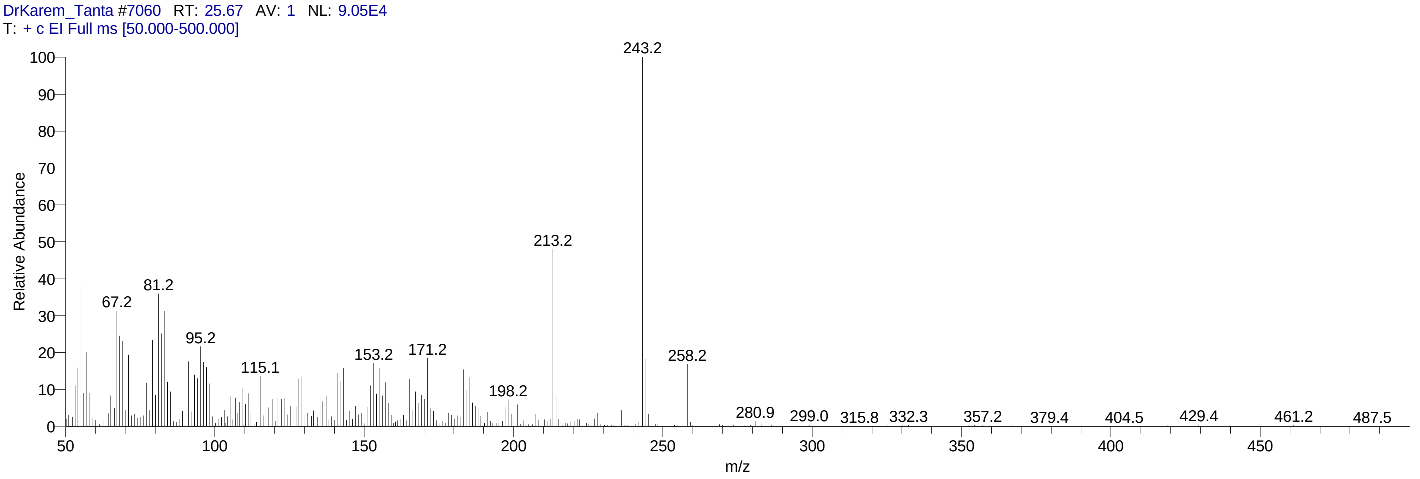

RT	Compound Name	Area %	MF	Molecular Formula	Molecular Weight	Cas #	Library
25.67	[1,2,4]TRIAZOLO[1,5-A][1,3,5]TRIAZIN-7-AMINE, 1,2-DIHYDRO-2-(4-METHOXYPHENYL)-2-METHYL-	0.70	855	C12H14N6O	258	132685-81-5	WileyRegistry8e
25.67	5-AMINO-2-(P-METHOXYMETHYL)-2-METHYL-2H-[1,2,4]TRIAZOLO[1,5-A][1,3,5]TRIAZINE	0.70	855	C12H14N6O	258	NA	WileyRegistry8e
25.67	19-NORKAUR-16-ENE, (4á)-	0.70	793	C19H30	258	76235-86-4	WileyRegistry8e
25.67	7-Acetyl-6-ethyl-1,1,4,4-tetramethyltetralin	0.70	797	C18H26O	258	88-29-9	replib
25.67	1-(3-ETHYL-5,5,8,8-TETRAMETHYL-5,6,7,8-TETRAHYDRO-2-NAPHTHALENYL)ETHANONE #	0.70	797	C18H26O	258	88-29-9	WileyRegistry8e

Compound Structure | Hit Spectrum

Formula C12H14N6O, MW 258, CAS# 132685-81-5, Entry# 147956
5-AMINO-2-(P-METHOXYPHENYL)-2-METHYL-2H-[1,2,4]TRIAZOLO[1,5-A][1,3,5]TRIAZINE

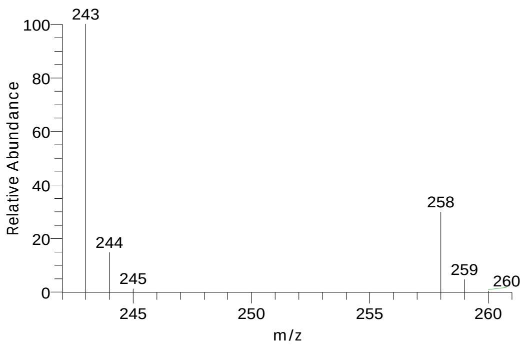

5-AMINO-2-(P-METHOXYMETHYL)-2-METHYL-2H-[1,2,4]TRIAZOLO[1,5-A][1,3,5]TRIAZINE
Formula C12H14N6O, MW 258, CAS# NA, Entry# 147957

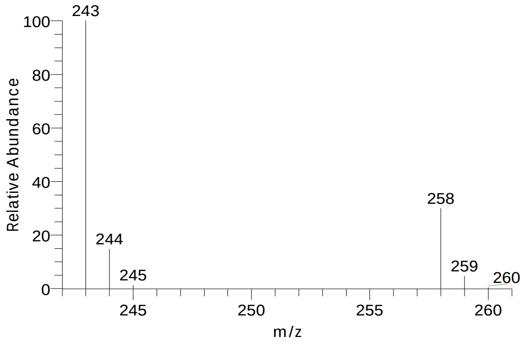

My GC-MS Report

Compound Structure | Hit Spectrum

19-NORKAUR-16-ENE, (4á)-
Formula C19H30, MW 258, CAS# 76235-86-4, Entry# 148957
(4á)-18-NORKAUR-16-ENE

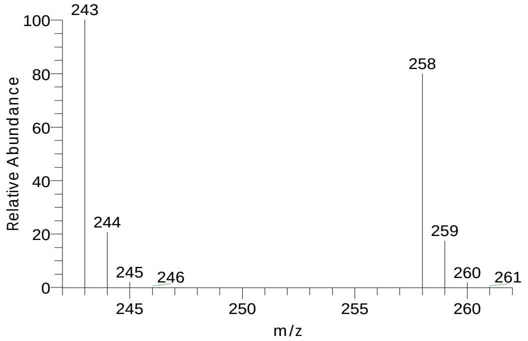

7-Acetyl-6-ethyl-1,1,4,4-tetramethyltetralin
Formula C18H26O, MW 258, CAS# 88-29-9, Entry# 30941
Versalide

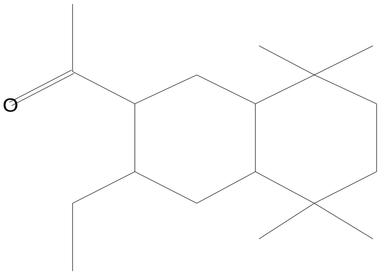

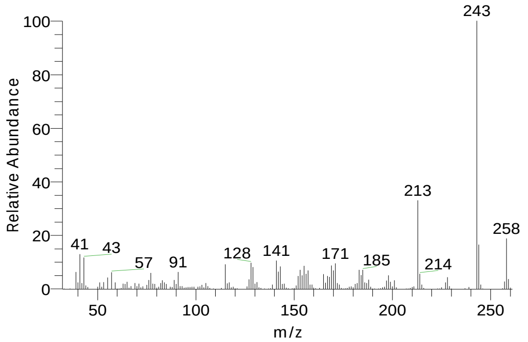

1-(3-ETHYL-5,5,8,8-TETRAMETHYL-5,6,7,8-TETRAHYDRO-2-NAPHTHALENYL)ETHANONE #
Formula C18H26O, MW 258, CAS# 88-29-9, Entry# 309414
1,1,4,4-TETRAMETHYL-6-ETHYL-7-ACETYL-1,2,3, 4-TETRAHYDRONAPHTHALENE

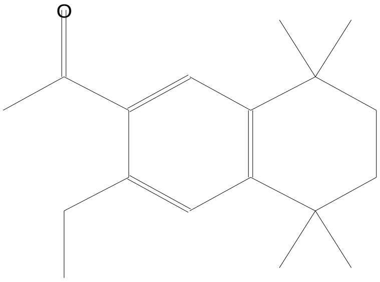

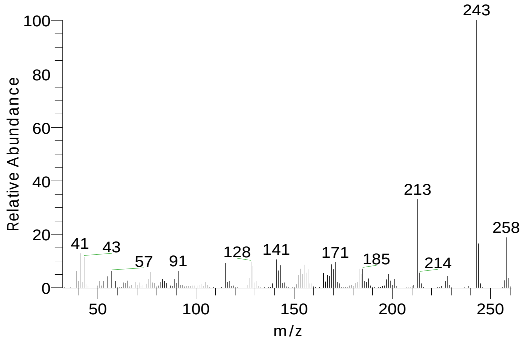

Supplement: Supplementary file 1 [file Supplementaryfile1.zip › GCMS Report Form.pdf]
